# Supplementary material for: Media content analysis of general practitioners’ reactions to care.data expressed in the media: what lessons can be learned for future NHS data-sharing initiatives?
Source: BMJ Open. 2020 Sep 10;10(9):e038006. doi: 10.1136/bmjopen-2020-038006 (PMC7485233; doi:10.1136/bmjopen-2020-038006)

Newspaper articles extracted from Nexis newspaper database:

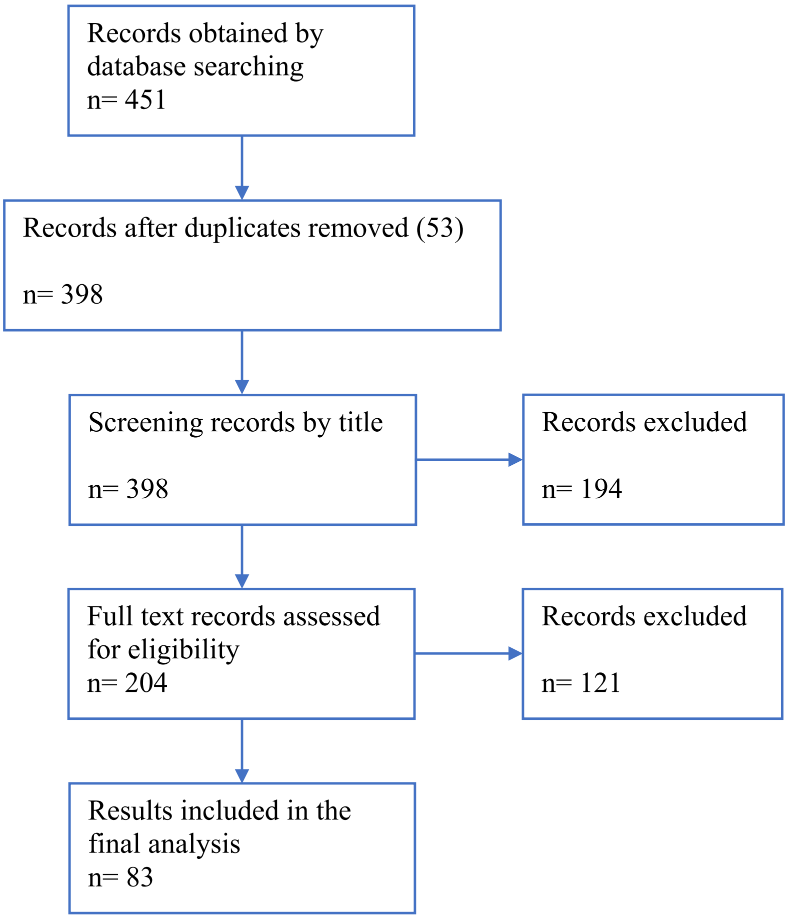

Web-based publications extracted from Nexis newspaper database:

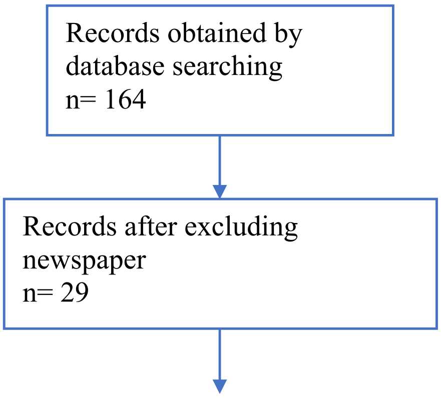

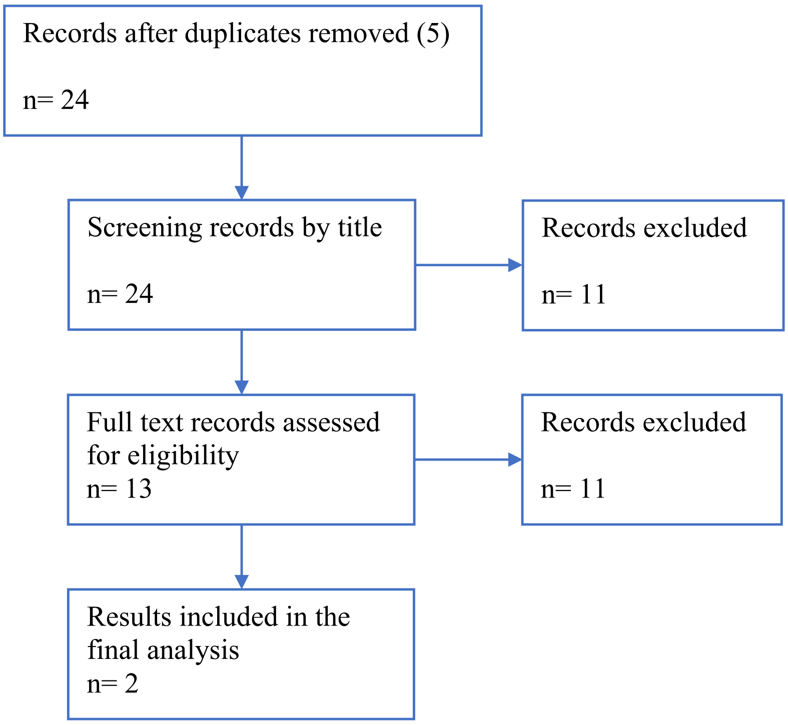

BBC news articles:

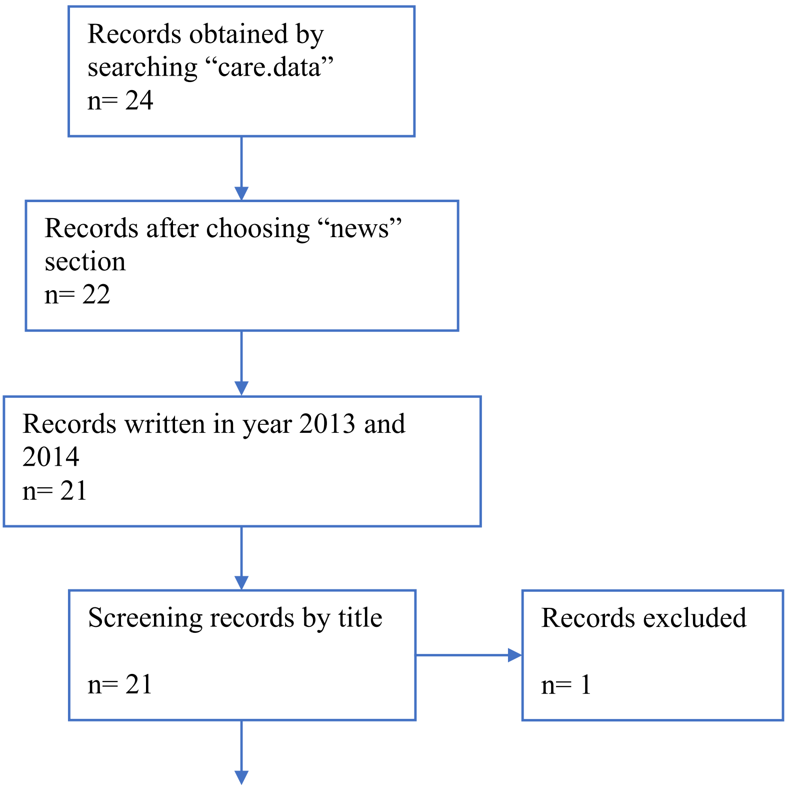

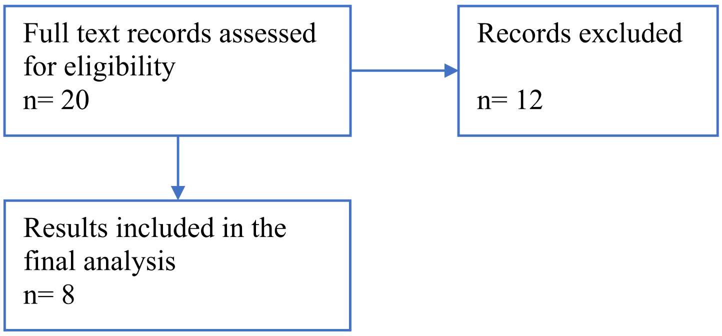

Pulse articles:

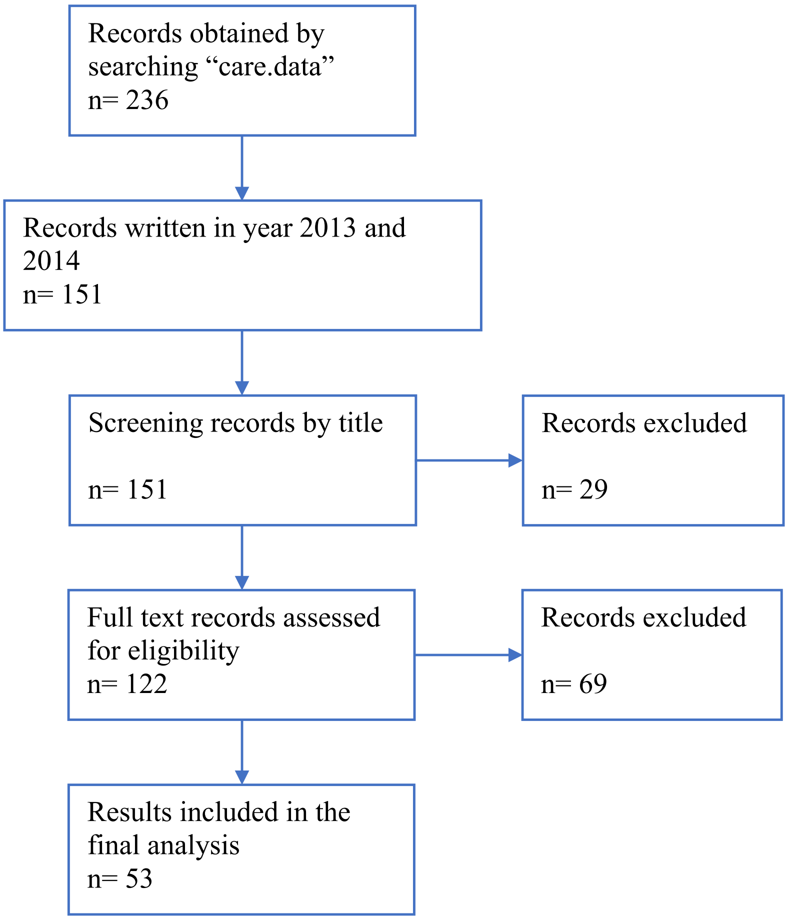

BMA articles:

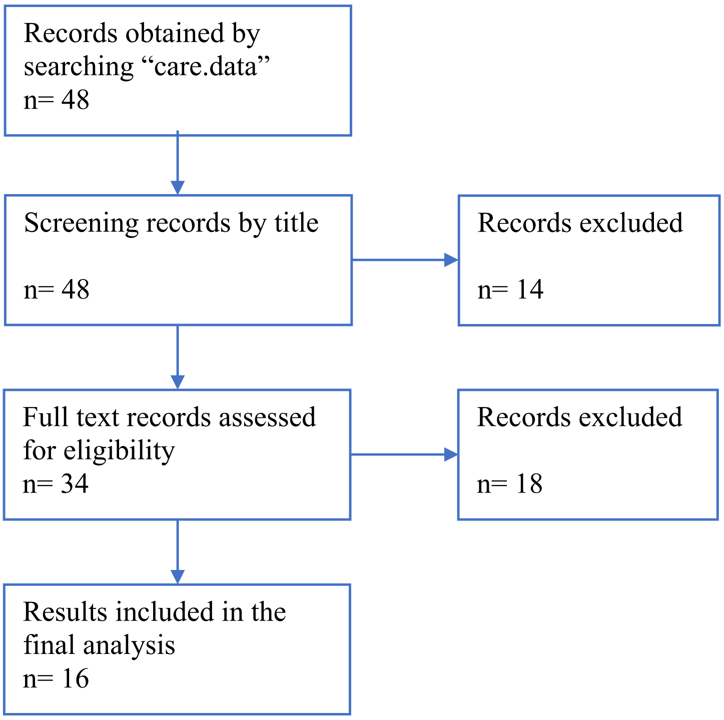

Supplement: Supplementary data [file bmjopen-2020-038006supp001.pdf]
