# Supplementary material for: Media content analysis of general practitioners’ reactions to care.data expressed in the media: what lessons can be learned for future NHS data-sharing initiatives?
Source: BMJ Open. 2020 Sep 10;10(9):e038006. doi: 10.1136/bmjopen-2020-038006 (PMC7485233; doi:10.1136/bmjopen-2020-038006)
Supplement: Supplementary data [file bmjopen-2020-038006supp002.pdf]

Appendix 1: All articles used in analysis

| Article number | Source type | Source name | Article title | URL | Author's name | Date |
|---|---|---|---|---|---|---|
| 1 | GP source | Pulse Today | NHS to link up data from GP records and secondary care | http://www.pulsetoday.co.uk/news/gp-topics/it/nhs-to-link-up-data-from-gp-records-and-secondary-care/20002260.article | Madlen Davies | 15/03/2013 |
| 2 | GP source | Pulse Today | Risk patients will be identified by anonymised GP record data, NHS England admits | http://www.pulsetoday.co.uk/news/gp-topics/it/risk-patients-will-be-identified-by-anonymised-gp-record-data-nhs-england-admits/20002593.article | Madlen Davies | 12/04/2013 |
| 3 | GP source | Pulse Today | Patients to be given 'veto' over their data being shared from GP records | http://www.pulsetoday.co.uk/news/gp-topics/it/patients-to-be-given-veto-over-their-data-being-shared-from-gp-records/20002754.article | Madlen Davies | 26/04/2013 |
| 4 | Newspaper | The Telegraph | Database of patient information takes first steps;<br>Confidential information from medical records held by family GPs will begin to be entered into a central database from next month. | https://www.telegraph.co.uk/news/health/news/10087957/Database-of-patient-information-takes-first-steps.html | Laura Donnelly | 29/05/2013 |
| 5 | GP source | BMA | Information sharing guidance offered to GPs | https://www.bma.org.uk/news/2013/may/information-sharing-guidance-offered-to-gps | Not stated | 29/05/2013 |
| 6 | GP source | Pulse Today | GPs face prosecution unless they inform patients their data could be used outside the NHS | http://www.pulsetoday.co.uk/news/gp-topics/it/gps-face-prosecution-unless-they-inform-patients-their-data-could-be-used-outside-the-nhs/20003860.article | Madlen Davies | 05/08/2013 |

| | | | | | | |
|---|---|---|---|---|---|---|
| 7 | GP source | Pulse Today | Private companies set for access to patient data for just £1 | http://www.pulsetoday.co.uk/news/gp-topics/it/private-companies-set-for-access-to-patient-data-for-just-1/20003879.article | Madlen Davies | 09/08/2013 |
| 8 | GP source | BMA | New rules for doctors ensuring data confidentiality | https://www.bma.org.uk/news/2013/september/new-rules-for-doctors-ensuring-data-confidentiality | Not stated | 13/09/2013 |
| 9 | GP source | Pulse Today | GP leaders consider boycott of NHS England's data extraction programme | http://www.pulsetoday.co.uk/home/finance-and-practice-life-news/gp-leaders-consider-boycott-of-nhs-englands-data-extraction-programme/20004354.article | Madlen Davies | 19/09/2013 |
| 10 | Newspaper | Mail Online | GPs threaten to boycott 'Big Brother' NHS database which would force them to send confidential patient records to private firms | https://www.dailymail.co.uk/news/article-2428211/GPs-threaten-boycott-Big-Brother-NHS-database.html | Stephen Adams | 22/09/2013 |
| 11 | GP source | Pulse Today | GPs unable to object to patient data being shared, warns GPC | http://www.pulsetoday.co.uk/news/gp-topics/it/gps-unable-to-object-to-patient-data-being-shared-warns-gpc/20004458.article | Madlen Davies | 26/09/2013 |
| 12 | GP source | Pulse Today | NHS data extraction programme poses 'enormous threat' to privacy | http://www.pulsetoday.co.uk/news/gp-topics/it/nhs-data-extraction-programme-poses-enormous-threat-to-privacy/20004575.article | Madlen Davies | 01/10/2013 |
| 13 | GP source | Pulse Today | Eight weeks to inform patients their data is going to be harvested, GPs warned | http://www.pulsetoday.co.uk/news/gp-topics/it/eight-weeks-to-inform-patients-their-data-is-going-to-be-harvested-gps-warned/20004562.article | Madlen Davies | 04/10/2013 |

| | | | | | | |
|---|---|---|---|---|---|---|
| 14 | Newspaper | The Independent | Care.data project to collate all NHS patients' records | https://www.independent.co.uk/life-style/health-and-families/health-news/caredata-project-to-collate-all-nhs-patients-records-8882379.html | Charlie Cooper | 16/10/2013 |
| 15 | GP source | Pulse Today | NHS England bows to confidentiality concerns and launches £2m national publicity campaign on care.data | http://www.pulsetoday.co.uk/nhs-england-bows-to-confidentiality-concerns-and-launches-2m-national-publicity-campaign-on-caredata/20004748.article | Sofia Lind | 16/10/2013 |
| 16 | GP source | BMA | GPs back patient-data awareness campaign | https://www.bma.org.uk/news/2013/october/gps-back-patient-data-awareness-campaign | Not stated | 16/10/2013 |
| 17 | Newspaper | Mail Online | NHS climbdown over 'big brother' database after the Mail on Sunday highlighted serious concerns over the plan | https://www.dailymail.co.uk/news/article-2468175/NHS-climbdown-big-brother-database-Mail-Sunday-highlighted-concerns-plan.html | Stephen Adams | 19/10/2013 |
| 18 | GP source | Pulse Today | Big data = big deal | http://www.pulsetoday.co.uk/views/blogs/big-data-big-deal/20004796.blog | Dr Hadrian Moss | 23/10/2013 |
| 19 | GP source | Pulse Today | Why I'm opting patients out of the care.data scheme | http://www.pulsetoday.co.uk/your-practice/comment/why-im-opting-patients-out-of-the-caredata-scheme/20005022.article | By one anonymous doctor | 12/11/2013 |
| 20 | GP source | Pulse Today | GP takes 'unlawful' decision to opt patients out of care.data programme | http://www.pulsetoday.co.uk/news/gp-topics/it/gp-takes-unlawful-decision-to-opt-patients-out-of-caredata-programme/20005018.article | Alex Matthews-King | 12/11/2013 |

| | | | | | | |
|---|---|---|---|---|---|---|
| 21 | GP source | Pulse Today | Second GP decides to opt all patients out of records extraction as care.data rebellion grows | http://www.pulsetoday.co.uk/news/gp-topics/it/second-gp-decides-to-opt-all-patients-out-of-records-extraction-as-caredata-rebellion-grows/20005097.article | One anonymous doctor | 19/11/2013 |
| 22 | Newspaper | The Observer | Fears grow over open access to patient records: Confidential information could be sold to the private sector | https://www.theguardian.com/society/2013/nov/24/fears-grow-open-access-patient-records | Paul Dinsdale | 24/11/2013 |
| 23 | GP source | Pulse Today | Biggest risk to care.data scheme is potential loss of GP 'confidence' in benefits of scheme | http://www.pulsetoday.co.uk/news/gp-topics/it/biggest-risk-to-caredata-scheme-is-potential-loss-of-gp-confidence-in-benefits-of-scheme/20005223.article | Not stated | 13/12/2013 |
| 24 | GP source | BMA | Awareness of patient data sharing drive begins | https://www.bma.org.uk/news/2014/january/awareness-of-patient-data-sharing-drive-begins | Not stated | 06/01/2014 |
| 25 | Newspaper | The Independent | Your life in their hands: is the care.data NHS database a healthy step or a gross invasion of patient privacy?; Doctors are worried that centralising our most personal records will break the bond of trust that underpins the NHS | https://www.independent.co.uk/life-style/health-and-families/health-news/your-life-in-their-hands-is-the-caredata-nhs-database-a-healthy-step-or-a-gross-invasion-of-patient-9042564.html | Charlie Cooper | 06/01/2014 |
| 26 | GP source | Pulse Today | Care.data - every little helps | http://www.pulsetoday.co.uk/views/blogs/caredata-every-little-helps/20005487.blog | Dr Hadrian Moss | 08/01/2014 |
| 27 | GP source | Pulse Today | GPs held responsible for patient complaints over NHS data-sharing project, says ICO | http://www.pulsetoday.co.uk/news/gp-topics/it/gps-held-responsible-for-patient-complaints-over-nhs-data-sharing-project-says-ico/20005505.article | Alex Matthews-King | 10/01/2014 |

| | | | | | | |
|---|---|---|---|---|---|---|
| 28 | Newspaper | The Daily Telegraph | Patients urged not to opt out of health database | Not available to the public | Sarah Knapton | 13/01/2014 |
| 29 | Newspaper | London Evening Standard | NHS data move opt-out 'damaging' | Not available to the public | Not stated | 13/01/2014 |
| 30 | Newspaper | The Telegraph | Health records of every NHS patient to be shared in vast database;<br>The health records of everyone in the NHS will be pooled in a vast database which can be accessed by researchers and pharmaceutical companies. But campaigners warn it could breach privacy. | https://www.telegraph.co.uk/news/10565160/Health-records-of-every-NHS-patient-to-be-shared-in-vast-database.html | Sarah Knapton | 13/01/2014 |
| 31 | Web publication | Sky News | NHS Data-Sharing Scheme 'Vital To Service' | https://news.sky.com/story/nhs-data-sharing-scheme-vital-to-service-10421691 | Not stated | 13/01/2014 |
| 32 | GP source | Pulse Today | Scientists launch campaign to discourage patients from opting out of care.data | http://www.pulsetoday.co.uk/news/scientists-launch-campaign-to-discourage-patients-from-opting-out-of-caredata/20005523.article | Alex Matthews-King | 13/01/2014 |
| 33 | GP source | Pulse Today | Revealed: Independent experts overseeing care.data have approved 31 releases of identifiable patient data since April | http://www.pulsetoday.co.uk/news/gp-topics/it/revealed-independent-experts-overseeing-caredata-have-approved-31-releases-of-identifiable-patient-data-since-april/20005572.article | Alex Matthews-King | 17/01/2014 |
| 34 | Newspaper | The Guardian | Patient records to be sold from NHS database: Fears anonymous health data could be matched to insurers' medical files | Not available to the public | Randeep Rames | 20/01/2014 |
| 35 | Newspaper | The Daily Telegraph | Don't mislead us about our medical records;<br>The NHS is asking us to hand over our private data - yet it cannot guarantee anonymity | https://www.telegraph.co.uk/news/nhs/10584402/Dont-mislead-us-about-our-NHS-medical-records.html | Philip Johnston | 21/01/2014 |

| | | | | | | |
|---|---|---|---|---|---|---|
| 36 | GP source | Pulse Today | MP tables motion to halt care.data rollout as 2,400 patients call helpline | http://www.pulsetoday.co.uk/news/gp-topics/it/mp-tables-motion-to-halt-caredata-rollout-as-2400-patients-call-helpline/20005621.article | Alex Matthews-King | 22/01/2014 |
| 37 | Newspaper | The Independent | 40 per cent of GPs plan to opt out of the NHS big data sweep, due to a lack of confidence in the project | https://www.independent.co.uk/life-style/health-and-families/health-news/40-per-cent-of-gps-plan-to-opt-out-of-the-nhs-big-data-sweep-due-to-a-lack-of-confidence-in-the-9083806.html | Charlie Cooper | 24/01/2014 |
| 38 | Newspaper | The Telegraph | Four in 10 GPs to opt out of NHS database; Family doctors raise concerns about new plans to share patients data | https://www.telegraph.co.uk/news/health/news/10595743/Four-in-10-GPs-to-opt-out-of-NHS-database.html | Laura Donnelly | 24/01/2014 |
| 39 | GP source | Pulse Today | Over 40% of GPs intend to opt themselves out of care.data scheme | http://www.pulsetoday.co.uk/news/gp-topics/it/over-40-of-gps-intend-to-opt-themselves-out-of-caredata-scheme/20005648.article | Nigel Praities | 24/01/2014 |
| 40 | Newspaper | i-Independent Print Ltd | GPs worried about confidentiality of patient data scheme;<br>HEALTH | Not available to the public | Charlie Cooper | 25/01/2014 |
| 41 | Media | BBC | Are your medical records in danger? | https://www.bbc.co.uk/news/health-25919399 | Nick Triggle | 28/01/2014 |
| 42 | GP source | Pulse Today | Dr Amir Hannan: 'This goes to the very heart of the doctor-patient relationship' | http://www.pulsetoday.co.uk/news/gp-topics/it/dr-amir-hannan-this-goes-to-the-very-heart-of-the-doctor-patient-relationship/20005677.article | Dr Amir Hannan | 28/01/2014 |
| 43 | GP source | Pulse Today | Professor Roger Jones: 'I strongly support the care.data initiative' | http://www.pulsetoday.co.uk/news/gp-topics/it/professor-roger-jones-i-strongly-support-the-caredata-initiative/20005693.article | Professor Roger Jones | 29/01/2014 |

| | | | | | | |
|---|---|---|---|---|---|---|
| | | | | | | |
| 44 | GP source | Pulse Today | GP survey reveals extent of care.data ignorance among patients and staff | http://www.pulsetoday.co.uk/news/gp-topics/it/gp-survey-reveals-extent-of-caredata-ignorance-among-patients-and-staff/20005713.article | Not stated | 31/01/2014 |
| 45 | GP source | Pulse Today | Analysis: GPs bear brunt of care.data confusion | http://www.pulsetoday.co.uk/views/analysis/analysis-gps-bear-brunt-of-caredata-confusion/20005720.article | Not stated | 31/01/2014 |
| 46 | GP source | Pulse Today | No mandate for care.data grab | Not available to the public | Not stated | 31/01/2014 |
| 47 | Newspaper | The Guardian | We trust no one with our data - not even our doctors: The Snowden revelations could prove bad for our health, thwarting a vital NHS scheme to gather clinical information | https://www.theguardian.com/commentisfree/2014/jan/31/nhs-medical-data-trust-doctors-edward-snowden | Jonathan Freedland | 01/02/2014 |
| 48 | Newspaper | The Guardian | Reply: Letter: What price our medical records? | https://www.theguardian.com/society/2014/feb/02/nhs-medical-records-care-data | Dr Alex May | 03/02/2014 |
| 49 | GP source | Pulse Today | What's so interesting? | http://www.pulsetoday.co.uk/views/blogs/whats-so-interesting/20005728.blog | Dr Hadrian Moss | 03/02/2014 |
| 50 | GP source | Pulse Today | GP hit with contract notice over plan to opt all patients out of care.data | http://www.pulsetoday.co.uk/news/gp-topics/it/gp-hit-with-contract-notice-over-plan-to-opt-all-patients-out-of-caredata/20005749.article | Alex Matthews-King | 04/02/2014 |

| | | | | | | |
|---|---|---|---|---|---|---|
| 51 | Newspaper | Daily Mail | HE OPTS HIS ENTIRE PRACTICE OUT OF SCHEME TO HARVEST MEDICAL DATA | Not available to the public | Andy Dolan And Sophie Borland | 05/02/2014 |
| 52 | Newspaper | Mail Online | NHS 'bullies' threaten to axe GP for keeping his patients' records private: He opts his entire practice out of scheme to harvest medical data | https://www.dailymail.co.uk/news/article-2551900/NHS-bullies-threaten-axe-GP-keeping-patients-records-private.html | Andy Dolan And Sophie Borland | 05/02/2014 |
| 53 | Newspaper | The Telegraph | GP fears losing job over pledge to keep patient data private;<br>A GP who pledged to keep his patients' confidential medical information private fears he could be ousted from his practice after NHS bosses said he could be found in breach of contract if he opts his patients out of the scheme | https://www.telegraph.co.uk/news/nhs/10618814/GP-fears-losing-job-over-pledge-to-keep-patient-data-private.html | Claire Carter | 05/02/2014 |
| 54 | Newspaper | Daily Mail | I won't give in to the NHS thought police who want to sell your private medical records | https://www.dailymail.co.uk/debate/article-2552717/I-wont-NHS-Thought-Police-want-sell-private-medical-records-GP-threatened-health-chiefs-hits-back.html | Dr Gordon Gancz | 06/02/2014 |
| 55 | GP source | Pulse Today | Saying no to care.data = saying no to care? | http://www.pulsetoday.co.uk/saying-no-to-caredata-saying-no-to-care/20005801.blog | Dr Tony Copperfield | 07/02/2014 |
| 56 | Newspaper | Daily Mail | INSURERS COULD USE NEW NHS DATABASE TO TRACK YOU DOWN WITHIN 2 HOURS' | https://www.dailymail.co.uk/news/article-2554437/Insurers-use-new-NHS-database-track-two-hours.html | Sophie Borland | 08/02/2014 |
| 57 | Newspaper | The Times | Share your data with the NHS. It's safe and beneficial;<br>Patient information will be secure in a confidential system and will be used only appropriately | https://www.thetimes.co.uk/article/share-your-data-with-the-nhs-its-safe-and-beneficial-cjmzw36s0jp | Dr Clare Gerada | 08/02/2014 |

| | | | | | | |
|---|---|---|---|---|---|---|
| 58 | Newspaper | Mail Online | Big Brother' database will grab children's health records but parents are being kept in the dark | https://www.dailymail.co.uk/news/article-2554959/Big-Brother-database-grab-childrens-health-records-parents-kept-dark.html | Martin Beckford | 09/02/2014 |
| 59 | Newspaper | The Telegraph | The brave doctor taking on Big Brother;<br>We owe a debt to the family GP who refuses to give up his patients' medical records to the Health and Social Care Information Centre | https://www.telegraph.co.uk/news/nhs/10627492/The-brave-doctor-taking-on-Big-Brother.html | Max Pemberton | 10/02/2014 |
| 60 | Newspaper | The Telegraph | Crisis of confidence' in NHS database, warn GPs;<br>Stall NHS data-sharing amid 'crisis of confidence' GPs say | https://www.telegraph.co.uk/news/health/news/10634539/Crisis-of-confidence-in-NHS-database-warn-GPs.html | Laura Donnelly | 12/02/2014 |
| 61 | Media | BBC | GPs voice fears over giant patient records database | https://www.bbc.co.uk/news/health-26151458 | Nick Triggle | 12/02/2014 |
| 62 | GP source | Pulse Today | RCGP demands new publicity campaign to address 'crisis in confidence' over care.data scheme | http://www.pulsetoday.co.uk/news/gp-topics/it/rcgp-demands-new-publicity-campaign-to-address-crisis-in-confidence-over-caredata-scheme/20005847.article | Alex Matthews-King | 12/02/2014 |
| 63 | Newspaper | Daily Mail | GPS WARN OF CRISIS IN PUBLIC CONFIDENCE OVER NHS DATABASE | https://www.dailymail.co.uk/news/article-2558135/GPs-warn-crisis-public-confidence-NHS-database-Royal-Collage-warns-health-service-failing-inform-patients-data-sharing.html | Sophie Borland | 13/02/2014 |
| 64 | Newspaper | The Times | GPs fear failure of patient data scheme | https://www.thetimes.co.uk/article/gps-fear-failure-of-patient-data-scheme-6csvc9wj3l2 | Chris Smyth | 13/02/2014 |

| | | | | | | |
|---|---|---|---|---|---|---|
| 65 | GP source | Pulse Today | Two-thirds of public 'don't recall receiving care.data information leaflet' | http://www.pulsetoday.co.uk/news/gp-topics/it/two-thirds-of-public-dont-recall-receiving-caredata-information-leaflet/20005874.article | Alex Matthews-King | 14/02/2014 |
| 66 | Newspaper | Mail Online | Two-thirds oppose plans for new NHS database that will see confidential medical records sold to private firms | https://www.dailymail.co.uk/news/article-2560335/Two-thirds-oppose-plans-new-NHS-database-confidential-medical-records-sold-private-firms.html | Not stated | 15/02/2014 |
| 67 | Newspaper | The Daily Telegraph | NHS admits database could pose privacy risk; NHS database could be disaster, say campaingers | https://www.telegraph.co.uk/news/nhs/10642740/NHS-admits-new-medical-records-database-could-pose-privacy-risk.html | Laura Donnelly | 17/02/2014 |
| 68 | Newspaper | The Independent | Your life in their hands: is the care.data NHS database a healthy step or a gross invasion of patient privacy?;<br>Doctors are worried that centralising our most personal records will break the bond of trust that underpins the NHS | https://www.independent.co.uk/life-style/health-and-families/health-news/your-life-in-their-hands-is-the-caredata-nhs-database-a-healthy-step-or-a-gross-invasion-of-patient-9042564.html | Charlie Cooper | 17/02/2014 |
| 69 | Newspaper | The Independent | Doctors raise fears over sharing NHS patient records;<br>BMA 'deeply concerned' with Government's public information campaign for care.data project | https://www.independent.co.uk/life-style/health-and-families/health-news/doctors-raise-fears-over-sharing-nhs-patient-records-9133807.html | Jane Kirby, Ella Pickover | 17/02/2014 |
| 70 | Newspaper | Mail Online | Now doctors' union has 'grave doubts' over plan to harvest patient data: British Medical Association warns public has been left in the dark over the scheme | https://www.dailymail.co.uk/news/article-2561662/Now-doctors-union-grave-doubts-plan-harvest-patient-data-British-Medical-Association-warns-public-left-dark-scheme.html | Sophie Borland | 17/02/2014 |
| 71 | Newspaper | London Evening Standard | Concern at medical records sharing | Not available to the public | Not stated | 17/02/2014 |

| | | | | | | |
|---|---|---|---|---|---|---|
| 72 | GP source | Pulse Today | GPC calls for urgent talks over public awareness of care.data scheme | http://www.pulsetoday.co.uk/news/gp-topics/it/gpc-calls-for-urgent-talks-over-public-awareness-of-caredata-scheme/20005884.article | Alex Matthews-King | 17/02/2014 |
| 73 | GP source | BMA | Call to boost public awareness of data sharing | https://www.bma.org.uk/news/2014/february/call-to-boost-public-awareness-of-data-sharing | Not stated | 17/02/2014 |
| 74 | Newspaper | The Daily Telegraph | Delay NHS database until public is dear, say experts | Not available to the public | Laura Donnelly | 18/02/2014 |
| 75 | Newspaper | Daily Mail | NOW DOCTORS' UNION HAS GRAVE DOUBTS' OVER PLAN TO HARVEST PATIENT DATA | https://www.dailymail.co.uk/news/article-2561662/Now-doctors-union-grave-doubts-plan-harvest-patient-data-British-Medical-Association-warns-public-left-dark-scheme.html | Sophie Borland | 18/02/2014 |
| 76 | Newspaper | The Guardian | NHS in England delays sharing of medical records | https://www.theguardian.com/society/2014/feb/18/nhs-delays-sharing-medical-records-care-data | Peter Walker | 18/02/2014 |
| 77 | Newspaper | The Independent | Victory for privacy as NHS database is delayed;<br>Collection of confidential medical records put on hold after backlash. Critics feared personal information would be vulnerable to hackers | https://www.independent.co.uk/life-style/health-and-families/health-news/victory-for-privacy-as-nhs-database-is-delayed-9137136.html | Charlie Cooper | 18/02/2014 |
| 78 | Newspaper | The Independent | Doctors raise fears over sharing NHS patient medical records;<br>BMA 'deeply concerned' with Government's public information campaign for care.data project | https://www.independent.co.uk/life-style/health-and-families/health-news/doctors-raise-fears-over-sharing-nhs-patient-records-9133807.html | Jane Kirby, Ella Pickover | 18/02/2014 |
| 79 | Newspaper | Mail Online | Controversial plan to share medical records across<br>NHS is put on hold for six months | Not available to public | Sam Webb | 18/02/2014 |

| | | | | | | |
|---|---|---|---|---|---|---|
| 80 | Newspaper | London Evening Standard | Medical records share plan delayed | https://www.standard.co.uk/panewsfeeds/medical-records-share-plan-delayed-9136528.html | Not stated | 18/02/2014 |
| 81 | Newspaper | London Evening Standard | Records plan clarification urged | Not available to the public | Not stated | 18/02/2014 |
| 82 | Newspaper | The Telegraph | Patients should be warned before NHS shares medical records, doctors say;<br>Growing pressure on ministers to go back to drawing board on NHS medical records | https://www.telegraph.co.uk/news/health/news/10646151/Patients-should-be-warned-before-NHS-shares-medical-records-doctors-say.html | Laura Donnelly | 18/02/2014 |
| 83 | Newspaper | The Times | Health chiefs delay data-sharing plan in big climbdown | https://www.thetimes.co.uk/article/nhs-chiefs-in-climbdown-over-sharing-patient-data-66396lf825q | Chris Smyth | 18/02/2014 |
| 84 | Web publication | Sky News | NHS Records Database Postponed Amid Concerns | https://news.sky.com/story/nhs-records-database-postponed-amid-concerns-10416834 | Not stated | 18/02/2014 |
| 85 | Media | BBC | Giant NHS database rollout delayed | https://www.bbc.co.uk/news/health-26239532 | Nick Triggle | 18/02/2014 |
| 86 | GP source | Pulse Today | NHS England delays care.data scheme to 'build understanding' of benefits | http://www.pulsetoday.co.uk/news/gp-topics/it/nhs-england-delays-caredata-scheme-to-build-understanding-of-benefits/20005896.article | Alex Matthews-King | 18/02/2014 |
| 87 | Newspaper | The Daily Telegraph | NHS database put on hold as officials accept privacy fears | Not available to the public | Laura Donnelly | 19/02/2014 |

| | | | | | | |
|---|---|---|---|---|---|---|
| 88 | Newspaper | Daily Mail | HOW CONCERNS GREW | Not available to the public | Sophie Borland | 19/02/2014 |
| 89 | Newspaper | The Guardian | NHS health record database delayed after public outcry: New system put on hold for at least six months: Critics say NHS England has failed to put case for it | Not available to the public | Peter Walker, James Meikle and Randeep Ramesh | 19/02/2014 |
| 90 | Newspaper | The Independent | Victory for privacy as NHS database delayed; * Collection of confidential medical records put on hold after backlash * Critics feared personal information would be vulnerable to hackers Data-sharing delay gives NHS 'time to get it right' | https://www.independent.co.uk/life-style/health-and-families/health-news/victory-for-privacy-as-nhs-database-is-delayed-9137136.html | Charlie Cooper | 19/02/2014 |
| 91 | Newspaper | Mail Online | NHS delays plan to harvest your details: Victory for the Mail as database is shelved for six months | https://www.dailymail.co.uk/news/article-2562296/Controversial-plan-share-medical-records-NHS-hold-six-months.html | Sophie Borland | 19/02/2014 |
| 92 | Newspaper | The Telegraph | NHS database 'vital' to improve cancer research;<br>Medical records database 'vital' for improving the country's poor cancer survival record, NHS chief says | https://www.telegraph.co.uk/news/health/news/10649786/NHS-database-vital-to-improve-cancer-research.html | Laura Donnelly | 19/02/2014 |
| 93 | Newspaper | The Times | NHS chiefs in climbdown over sharing patient data | https://www.thetimes.co.uk/article/nhs-chiefs-in-climbdown-over-sharing-patient-data-66396lf825q | Chris Smyth | 19/02/2014 |
| 94 | GP source | Pulse Today | Rebel GP told contract notice over care.data opt-outs was a 'misunderstanding' | http://www.pulsetoday.co.uk/news/gp-topics/it/rebel-gp-told-contract-notice-over-caredata-opt-outs-was-a-misunderstanding/20005908.article | Alex Matthews-King | 19/02/2014 |

| | | | | | | |
|---|---|---|---|---|---|---|
| 95 | GP source | BMA | Doctors' concerns prompt data sharing plan delay | https://www.bma.org.uk/news/2014/february/doctors-concerns-prompt-data-sharing-plan-delay | Not stated | 19/02/2014 |
| 96 | Newspaper | The Daily Telegraph | Database a fundamental part of the fight against cancer, says NHS chief;<br>Director defends scheme as crucial to future of the health service | Not available to the public | Laura Donnelly | 20/02/2014 |
| 97 | Newspaper | The Times | NHS must quell fears over data or lives will be lost, says charity | https://www.thetimes.co.uk/article/nhs-must-quell-fears-over-data-or-lives-will-be-lost-says-charity-z5rj2m7qwhc | Michael Savage and Chris Smyth | 20/02/2014 |
| 98 | Media | BBC | Critics of giant NHS database 'are scaremongering' | https://www.bbc.co.uk/news/health-26277866 | Nick Triggle and Adam Brimelow | 21/02/2014 |
| 99 | GP source | BMA | GPC Newsletter | https://www.bma.org.uk/connecting-doctors/the_practice/m/mediagallery/24 | Not stated | 21/02/2014 |
| 100 | Newspaper | The Daily Telegraph | Data don't help cancer;<br>Letters to the Editor | Not available to public | Dr Neil Bhatia | 22/02/2014 |
| 101 | Newspaper | The Telegraph | NHS medical records database could help prevent disease, senior doctors say;<br>Lord Darzi and Lord Crisp have joined a group of health professionals to defend the NHS medical records database, days after it was put on hold following concerns about data sharing | https://www.telegraph.co.uk/news/health/news/10657580/NHS-medical-records-database-could-help-prevent-disease-senior-doctors-say.html | Claire Carter | 24/02/2014 |
| 102 | Newspaper | The Times | Healthcare data;<br>If data linkage in the UK had not been undertaken in the past we would know less about the causes of disease and be ignorant of inequities in access to care | Not available to the public | Not stated | 24/02/2014 |

| | | | | | | |
|---|---|---|---|---|---|---|
| 103 | Newspaper | The Times | Medical advances need sharing of data, say doctors | https://www.thetimes.co.uk/article/medical-advances-need-sharing-of-data-say-doctors-s6flk6l6dn2 | Oliver Moody | 24/02/2014 |
| 104 | Media | BBC | NHS data-sharing project at risk, say MPs | https://www.bbc.co.uk/news/health-26347026 | Nick Triggle | 25/02/2014 |
| 105 | Media | BBC | NHS data-sharing will have 'enormous benefits', say peers | http://www.bbc.co.uk/democracylive/house-of-lords-26343037 | Not stated | 25/02/2014 |
| 106 | GP source | Pulse Today | Debate: Should GPs back care.data? | http://www.pulsetoday.co.uk/news/gp-topics/it/debate-should-gps-back-caredata/20005959.article | Not stated | 25/02/2014 |
| 107 | Newspaper | The Independent | Atos to manage NHS care.data project despite ongoing 'mess' over disability benefit assessments; Despite the Disability Minister Mike Penning admitting the reassessments were in 'chaos' Atos will roll out the controversial care.data project | https://www.independent.co.uk/news/atos-to-work-on-nhs-care-data-project-despite-ongoing-mess-over-disability-benefit-assessments-9153885.html | Felicity Morse | 26/02/2014 |
| 108 | Newspaper | The Times | GPs force rethink on data-sharing plan; | https://www.thetimes.co.uk/article/gps-force-rethink-on-data-sharing-plan-hlkzfj96qzs | Michael Savage | 26/02/2014 |
| 109 | Newspaper | The Times | The pitfalls of the NHS central database plan; Letters to the Editor | https://www.thetimes.co.uk/article/the-pitfalls-of-the-nhs-central-database-plan-qts55x7pdjq | Not stated | 26/02/2014 |
| 110 | Newspaper | The Times | Opt-out easier in GP data-sharing rethink | Not available to the public | Michael Savage | 26/02/2014 |

| | | | | | | |
|---|---|---|---|---|---|---|
| 111 | GP source | Pulse Today | Three in four GPs believe care.data should be 'opt in' | http://www.pulsetoday.co.uk/news/gp-topics/it/three-in-four-gps-believe-caredata-should-be-opt-in/20005954.article | Alex Matthews-King | 26/02/2014 |
| 112 | GP source | BMA | Patient trust threatened by data-sharing concerns | https://www.bma.org.uk/news/2014/february/patient-trust-threatened-by-data-sharing-concerns | Not stated | 26/02/2014 |
| 113 | Newspaper | The Daily Telegraph | GPs back 'opt-in' NHS database | Not available to the public | Not stated | 27/02/2014 |
| 114 | Newspaper | The Independent | Atos to work on NHS care data project despite ongoing 'mess' over disability benefit assessments;<br>Despite the Disability Minister Mike Penning admitting the reassessments were in 'chaos' Atos will roll out the controversial care.data project | https://www.independent.co.uk/news/atos-to-work-on-nhs-care-data-project-despite-ongoing-mess-over-disability-benefit-assessments-9153885.html | Felicity Morse | 27/02/2014 |
| 115 | Newspaper | Mail Online | Cashing in on patient records to be banned: But you'll still have to opt out to keep private details off database | https://www.dailymail.co.uk/news/article-2570567/Cashing-patient-records-banned-But-youll-opt-private-details-database.html | James Chapman and Andy Dolan | 28/02/2014 |
| 116 | GP source | Pulse Today | Update: Care.data put on hold | http://www.pulsetoday.co.uk/views/analysis/update-caredata-put-on-hold/20005997.article | Nigel Praities | 28/02/2014 |
| 117 | Newspaper | The Daily Telegraph | NHS data for sale: will your premiums rise?; Insurance Nicole Blackmore examines the Government's plans for a new database and how it could be exploited by insurers | Not available to the public | Nicole Blackmore | 01/03/2014 |
| 118 | Newspaper | Daily Mail | CASHING IN ON PATIENT RECORDS TO BE BANNED | https://www.dailymail.co.uk/news/article-2570567/Cashing-patient-records-banned-But-youll-opt-private-details-database.html | James Chapman And Andy Dolan | 01/03/2014 |

| | | | | | | |
|---|---|---|---|---|---|---|
| 119 | Newspaper | London Evening Standard | NHS data will not be sold: Hunt | https://www.standard.co.uk/panewsfeeds/nhs-data-will-not-be-sold-hunt-9161694.html | Not stated | 01/03/2014 |
| 120 | Newspaper | The Times | NHS patient data to be made confidential | https://www.thetimes.co.uk/article/nhs-patient-data-to-be-made-confidential-gdxmctdnh56 | Tom Whipple | 01/03/2014 |
| 121 | GP source | Pulse Today | Legislation will prevent patient data being sold outside the health service, says Hunt | http://www.pulsetoday.co.uk/news/gp-topics/it/legislation-will-prevent-patient-data-being-sold-outside-the-health-service-says-hunt/20006014.article | Alex Matthews-King | 03/03/2014 |
| 122 | Newspaper | The Daily Telegraph | Hospital records used by private marketing firms;<br>NHS data helps firms 'tailor media campaigns' | https://www.telegraph.co.uk/news/nhs/10674639/NHS-hospital-records-used-by-private-marketing-firms.html | Laura Donnelly | 04/03/2014 |
| 123 | Newspaper | Mail Online | BY THE WAY . . . GP RECORDS DON'T TELL THE WHOLE STORY | Not available to the public | Dr Martin Scurr | 04/03/2014 |
| 124 | Newspaper | The Guardian | Reply: Letter: Taking better care of health data | https://www.theguardian.com/society/2014/mar/04/taking-better-care-health-data | Various<br>Prof Harvey Goldstein University of Bristol, Prof Ruth Gilbert University College London, Dr Katie Harron UCL, Dr Gareth Hagger-Johnson UCL, Dr Mario Cortina UCL , | 05/03/2014 |

| | | | | | | |
|---|---|---|---|---|---|---|
| | | | | | Dr Nirupa Dattani City University | |
| 125 | Newspaper | The Guardian | Reply: Letter: Taking better care of health data | https://www.theguardian.com/society/2014/mar/04/taking-better-care-health-data | Professor Robert Boyd | 05/03/2014 |
| 126 | Newspaper | The Telegraph | NHS database: will it push up your insurance premiums?;<br>Experts fear insurers will buy medical information about customers and use it to price policies | https://www.telegraph.co.uk/finance/personalfinance/insurance/10667245/NHS-database-will-it-push-up-your-insurance-premiums.html | Nicole Blackmore | 05/03/2014 |
| 127 | GP source | Pulse Today | Falling off the care.data fence | http://www.pulsetoday.co.uk/views/blogs/falling-off-the-caredata-fence/20006012.blog | Not stated | 05/03/2014 |
| 128 | GP source | BMA | GPC Newsletter | Not available to the public<br><br>file:///Users/yaldakazempour/Downloads/11%20GPC%20Newsletter%2006%2003%2014%20(3).pdf | Not stated | 06/03/2014 |
| 129 | Newspaper | The Times | Health data centre pledges more honesty | https://www.thetimes.co.uk/article/health-data-centre-pledges-more-honesty-m7qpfbtx8qf | Chris Smyth | 07/03/2014 |
| 130 | GP source | BMA | BMA remains concerned over special measures safeguards | https://www.bma.org.uk/news/2014/march/bma-remains-concerned-over-special-measures-safeguards | Not stated | 07/03/2014 |
| 131 | Media | BBC | Government outlines data-sharing safeguards | http://www.bbc.co.uk/democracylive/house-of-commons-26518046 | Not stated | 10/03/2014 |

| | | | | | | |
|---|---|---|---|---|---|---|
| 132 | Media | BBC | MPs agree to data-sharing safeguards | http://www.bbc.co.uk/democracylive/house-of-commons-26532173 | Not stated | 11/03/2014 |
| 133 | Newspaper | The Times | Sharing medical data would turn UK into 'best clinical laboratory in world' | https://www.thetimes.co.uk/article/sharing-medical-data-would-turn-uk-into-best-clinical-laboratory-in-world-sg639w8m5cj | Chris Smyth | 21/03/2014 |
| 134 | GP source | Pulse Today | Patients would 'embrace' care.data if they understood it, jet lag cure, crackdown on slum landlords and exploding cells to fight cancer | http://www.pulsetoday.co.uk/patients-would-embrace-caredata-if-they-understood-it-jet-lag-cure-crackdown-on-slum-landlords-and-exploding-cells-to-fight-cancer/20006171.article | Christian Duffin | 21/03/2014 |
| 135 | GP source | BMA | Care.data confidentiality concerns cannot be ignored, say doctors | https://www.bma.org.uk/news/2014/march/caredata-confidentiality-concerns-cannot-be-ignored-say-doctors | Dr Lucy-Jane Davis | 21/03/2014 |
| 136 | GP source | BMA | GPC Newsletter | Not available to the public | Not stated | 21/03/2014 |
| 137 | Newspaper | The Independent | Medical chief defends NHS data-sharing; HEALTH | Not available to the public | Paul Bignell | 22/03/2014 |
| 138 | Newspaper | Daily Mail | MORE DELAYS FOR NHS DATA HARVESTING PROGRAMME | https://www.dailymail.co.uk/news/article-2608134/More-delays-controversial-NHS-data-harvesting-programme-Government-adviser-says-scheme-mishandled.html | Not stated | 19/04/2014 |

| | | | | | | |
|---|---|---|---|---|---|---|
| 139 | GP source | BMA | GPC Newsletter | https://www.bma.org.uk/connecting-doctors/the_practice/m/mediagallery/48 | | 22/04/2014 |
| 140 | Newspaper | The Daily Telegraph | Patient data in peril;<br>Letters to the Editor | Not available to the public | Professor John R Ashton | 07/05/2014 |
| 141 | GP source | Pulse Today | Practice opts all patients out of care.data despite delay to roll-out | http://www.pulsetoday.co.uk/news/gp-topics/it/practice-opts-all-patients-out-of-caredata-despite-delay-to-roll-out/20006682.article | Not stated | 12/05/2014 |
| 142 | Newspaper | The Telegraph | Britons 'trust banks more than government' to protect their data;<br>The government has much work to do reassuring the public of its data security measures, according to Unisys | https://www.telegraph.co.uk/technology/internet-security/10846656/Britons-trust-banks-more-than-government-to-protect-their-data.html | Sophie Curtis | 21/05/2014 |
| 143 | GP source | Pulse Today | GPs vote in favour of an opt-in system for care.data | http://www.pulsetoday.co.uk/news/gp-topics/it/gps-vote-in-favour-of-an-opt-in-system-for-caredata/20006796.article | Alex Matthews-King | 23/05/2014 |
| 144 | GP source | BMA | Patient data extraction requires explicit consent, say GPs | https://www.bma.org.uk/news/2014/may/patient-data-extraction-requires-explicit-consent-say-gps | Not stated | 27/05/2014 |
| 145 | Newspaper | The Times | GPs force rethink on data-sharing plan | https://www.thetimes.co.uk/article/gps-force-rethink-on-data-sharing-plan-hlkzfj96qzs | Michael Savage | 17/06/2014 |
| 146 | Newspaper | Daily Mail | AND OFFICIALS DON'T EVEN KNOW WHERE DETAILS OF 1.3M PATIENTS ENDED UP | Not available to the public | Sophie Borland | 18/06/2014 |

| | | | | | | |
|---|---|---|---|---|---|---|
| 147 | GP source | Pulse Today | Parts of care.data should be 'opt-in' only, says NHS England director | http://www.pulsetoday.co.uk/news/gp-topics/it/parts-of-caredata-should-be-opt-in-only-says-nhs-england-director/20007039.article | Sofia Lind | 20/06/2014 |
| 148 | Newspaper | Mail Online | CONSENT CONCERNS ON MEDICAL RECORDS | Not available to the public | Not stated | 25/06/2014 |
| 149 | GP source | Pulse Today | Over half the public still unaware of care.data | http://www.pulsetoday.co.uk/news/gp-topics/it/over-half-the-public-still-unaware-of-caredata/20007087.article | Alex Matthews-King | 25/06/2014 |
| 150 | GP source | BMA | Patients' medical data sacrosanct, declares BMA | https://www.bma.org.uk/news/2014/june/patients-medical-data-sacrosanct-declares--bma | Not stated | 25/06/2014 |
| 151 | GP source | Pulse Today | GPs to send patient letters explaining care.data 'opt-out' | http://www.pulsetoday.co.uk/news/gp-topics/it/gps-to-send-patient-letters-explaining-caredata-opt-out/20007191.article | Alex Matthews-King | 03/07/2014 |
| 152 | GP source | BMA | Call to involve patients in how data is shared | https://www.bma.org.uk/news/2014/july/call-to-involve-patients-in-how-data-is-shared | Not stated | 03/07/2014 |
| 153 | GP source | Pulse Today | Make care.data opt in or we will opt patients out, say GPs | http://www.pulsetoday.co.uk/news/gp-topics/it/make-caredata-opt-in-or-we-will-opt-patients-out-say-gps/20007472.article | Alex Matthews-King | 05/08/2014 |
| 154 | GP source | Pulse Today | GPs' care.data responsibilities amount to 'good customer service', says information commissioner | http://www.pulsetoday.co.uk/news/gp-topics/it/gps-caredata-responsibilities-amount-to-good-customer-service-says-information-commissioner/20007450.article | Alex Matthews-King | 05/08/2014 |

| | | | | | | |
|---|---|---|---|---|---|---|
| 155 | GP source | Pulse Today | Police should have greater access to medical records, says chief constable | http://www.pulsetoday.co.uk/news/gp-topics/it/police-should-have-greater-access-to-medical-records-says-chief-constable/20007529.article | Alex Matthews-King | 11/08/2014 |
| 156 | GP source | Pulse Today | NHS England looks at expanding care.data extractions to include 'sensitive' patient information | http://www.pulsetoday.co.uk/news/gp-topics/it/nhs-england-looks-at-expanding-caredata-extractions-to-include-sensitive-patient-information/20007611.article | Alex Matthews-King | 19/08/2014 |
| 157 | GP source | Pulse Today | Selling patient data poses risks to GPs and patients alike | http://www.pulsetoday.co.uk/views/blogs/inside-information/selling-patient-data-poses-risks-to-gps-and-patients-alike/20007916.blog | Dr Hadrian Moss | 17/09/2014 |
| 158 | GP source | Pulse Today | GPs could opt all patients out of care.data opt under data protection laws, information office says | http://www.pulsetoday.co.uk/news/gp-topics/it/gps-could-opt-all-patients-out-of-caredata-opt-under-data-protection-laws-information-office-says/20008114.article | Alex Matthews-King | 06/10/2014 |
| 159 | Newspaper | Mail Online | Storm as NHS gives go-ahead to patient database despite concerns: Pilot scheme will involve 1.7million people unless an individual specifically ops out | https://www.dailymail.co.uk/news/article-2784269/Storm-NHS-gives-ahead-patient-database-despite-concerns-Pilot-scheme-involve-1-7million-people-unless-individual-specifically-ops-out.html | Sophie Borland | 07/10/2014 |
| 160 | GP source | Pulse Today | GPs to contact patients individually to explain care.data opt-out | http://www.pulsetoday.co.uk/news/gp-topics/it/gps-to-contact-patients-individually-to-explain-caredata-opt-out/20008134.article | Alex Matthews-King | 07/10/2014 |

| | | | | | | |
|---|---|---|---|---|---|---|
| 161 | Newspaper | Mail Online | NHS data is snooped on six times every day: Staff caught looking at records of friends, family and even love rivals | https://www.dailymail.co.uk/news/article-2833960/NHS-data-snooped-six-times-day-Staff-caught-looking-records-friends-family-love-rivals.html?ITO=1490&ns_mchannel=rss&ns_campaign=1490 | Sophie Borland | 14/11/2014 |
| 162 | GP source | Pulse Today | Care.data extractions on hold until NHS England satisfies GP concerns | http://www.pulsetoday.co.uk/news/gp-topics/it/caredata-extractions-on-hold-until-nhs-england-satisfies-gp-concerns/20008789.article | Alex Matthews-King | 18/12/2014 |
