# Supplementary material for: Media content analysis of general practitioners’ reactions to care.data expressed in the media: what lessons can be learned for future NHS data-sharing initiatives?
Source: BMJ Open. 2020 Sep 10;10(9):e038006. doi: 10.1136/bmjopen-2020-038006 (PMC7485233; doi:10.1136/bmjopen-2020-038006)
Supplement: Author's manuscript [file bmjopen-2020-038006.draft_revisions.pdf]

BMJ Open is committed to open peer review. As part of this commitment we make the peer review history of every article we publish publicly available.

When an article is published we post the peer reviewers' comments and the authors' responses online. We also post the versions of the paper that were used during peer review. These are the versions that the peer review comments apply to.

The versions of the paper that follow are the versions that were submitted during the peer review process. They are not the versions of record or the final published versions. They should not be cited or distributed as the published version of this manuscript.

BMJ Open is an open access journal and the full, final, typeset and author-corrected version of record of the manuscript is available on our site with no access controls, subscription charges or pay-per-view fees (http://bmjopen.bmj.com).

If you have any questions on BMJ Open's open peer review process please email info.bmjopen@bmj.com

BMJ Open

Analysis of general practitioners' reactions to care.data expressed in the media: what lessons can be learned for future NHS data sharing initiatives?

Journal:	*BMJ Open*
Manuscript ID	bmjopen-2020-038006
Article Type:	Original research
Date Submitted by the Author:	25-Feb-2020

Complete List of Authors:	Ford, Elizabeth; Brighton & Sussex Medical School,<br>Kazempour, Yalda; Brighton & Sussex Medical School, Primary Care and Public Health<br>Cooper, Maxwell; Brighton & Sussex Medical School<br>Katikireddi, Srinivasa; University of Glasgow School of Life Sciences, MRC/CSO Social & Public Health Sciences Unit<br>Boyd, Andy; University of Bristol, School of Social and Community Medicine

Keywords:	Health informatics < BIOTECHNOLOGY & BIOINFORMATICS, EPIDEMIOLOGY, MEDICAL ETHICS, PRIMARY CARE, PUBLIC HEALTH

SCHOLARONE™
Manuscripts

I, the Submitting Author has the right to grant and does grant on behalf of all authors of the Work (as defined in the below author licence), an exclusive licence and/or a non-exclusive licence for contributions from authors who are: i) UK Crown employees; ii) where BMJ has agreed a CC-BY licence shall apply, and/or iii) in accordance with the terms applicable for US Federal Government officers or employees acting as part of their official duties; on a worldwide, perpetual, irrevocable, royalty-free basis to BMJ Publishing Group Ltd ("BMJ") its licensees and where the relevant Journal is co-owned by BMJ to the co-owners of the Journal, to publish the Work in this journal and any other BMJ products and to exploit all rights, as set out in our licence.

The Submitting Author accepts and understands that any supply made under these terms is made by BMJ to the Submitting Author unless you are acting as an employee on behalf of your employer or a postgraduate student of an affiliated institution which is paying any applicable article publishing charge ("APC") for Open Access articles. Where the Submitting Author wishes to make the Work available on an Open Access basis (and intends to pay the relevant APC), the terms of reuse of such Open Access shall be governed by a Creative Commons licence – details of these licences and which Creative Commons licence will apply to this Work are set out in our licence referred to above.

Other than as permitted in any relevant BMJ Author's Self Archiving Policies, I confirm this Work has not been accepted for publication elsewhere, is not being considered for publication elsewhere and does not duplicate material already published. I confirm all authors consent to publication of this Work and authorise the granting of this licence.

For peer review only

Analysis of general practitioners' reactions to care.data expressed in the media: what lessons can be learned for future NHS data sharing initiatives?

Elizabeth Ford[1]*, Yalda Kazempour[1], Max Cooper[1], Srinivasa Vittal Katikireddi[2], Andy Boyd[3]

1: Department of Primary Care and Public Health, Brighton and Sussex Medical School, Brighton UK.

2: MRC/CSO Social & Public Health Sciences Unit, University of Glasgow

3: Population Health Sciences, Bristol Medical School, University of Bristol, Bristol, UK.

*Corresponding Author:

Dr Elizabeth Ford
Senior Lecturer in Primary Care Research
Department of Primary Care and Public Health
Brighton and Sussex Medical School
Room 322 Watson Building
Village Way,
Falmer
BRIGHTON
BN1 9PH
+44 (0) 1273 641974
e.m.ford@bsms.ac.uk

For peer review only

For peer review only - http://bmjopen.bmj.com/site/about/guidelines.xhtml

Abstract

Objectives: Care.data was a 2013 UK government initiative to extract patient data from general practices in England to form a centralised whole-population database for service planning and health research. After a public outcry, the scheme was postponed and cancelled. Public views of care.data have previously been analysed; this study aimed to understand contemporary general practitioners' (GPs) views of the scheme, which may have been influential in its downfall.

Design: Systematic search of media articles, followed by media content analysis.

Setting: UK-based mainstream and GP-facing media in 2013 and 2014.

Participants: Articles were eligible if they focused on care.data, and GPs were quoted, authored the article, or if articles were written for a majority GP audience.

Interventions: N/A

Primary and Secondary outcome measures: Themes which explained GPs reactions to care.data and which could explain support for or opposition to the scheme.

Results: 162 media articles met inclusion criteria and were drawn from newspapers, news websites and GP facing websites. GPs recognised care.data's potential value for research and improving care, but had grave concerns about the scheme's implementation. These centred the lack of safeguards and purpose around the scheme which meant patients were not able to make informed decisions about opt-out. GPs perceived they were poorly resourced to meet competing demands to both share patients' data and protect confidentiality. They distrusted the government's likely uses of the data and perceived a risk of patient re-identification if the data were sold onto to commercial entities.

Conclusions: Findings show specific concerns which GPs had about care.data which led to the withdrawal of support. Future NHS patient data-sharing schemes should engage with GPs and other clinicians as key stakeholders from the earliest moments of planning, so that their views and needs are incorporated into the design of such schemes.

Strengths and Limitations of this Study:

- We systematically and comprehensively searched mainstream and GP facing media published during the unfolding care.data events, to access the voice of doctors as events unfolded, thus minimising recall bias in accounts of events.

For peer review only - http://bmjopen.bmj.com/site/about/guidelines.xhtml

- By following a systematic approach, we minimised bias in the selection of articles for the study. However, other sources of GP opinions (such as social media discussions) escaped our analysis.

- Doctors contributing to mainstream news articles may not be representative of GPs in general, but instead can be viewed as key contributors to the prominent circulating discourses at the time.

- GPs views expressed at the time of the debate may not represent their current views on the care.data scheme, its subsequent failure, or on future schemes with similar aims.

Funding Statement:

This research received no specific grant from any funding agency in the public, commercial or not-for-profit sectors.

SVK acknowledges funding from a NRS Senior Clinical Fellowship (SCAF/15/02), the Medical Research Council (MRC) (MC_UU_12017/13 & MC_UU_12017/15) and the Scottish Government Chief Scientist Office (SPHSU13 & SPHSU15).

AB acknowledges funding from the Avon Longitudinal Study of Parents and Children: MRC and Wellcome (Grant ref: 102215/2/13/2) and the CLOSER longitudinal research consortium: Economic and Social Research Council (ES/K000357/).

Competing Interest:

The authors declare no competing interests.

For peer review only

For peer review only - http://bmjopen.bmj.com/site/about/guidelines.xhtml

Introduction

Care.data

In 2012, laws were passed within the United Kingdom Health and Social Care Act (HSCA) to require all general practitioners (GPs; family doctors) in England to upload their electronic patient records (EPRs) to a central database which could then be linked with additional NHS datasets and used for research, audit, and planning health services. This programme of data collation and re-use was called care.data and was due to be initiated in Spring 2014 [1]. It expanded upon previous data collections which were limited to hospital records or were opt-in at the GP practice level [2-5]. For care.data and previous schemes, provisions were made so that individual patients who did not wish their data to be used for quality improvement and research purposes could opt-out [1].

The care.data scheme was advertised to patients in the form of a leaflet "*Better information means better care*" [6] which was purportedly delivered to all households in England by Royal Mail in January 2014, although few people reported receiving it [7]. There was no opt-out form on the leaflet and no clear instructions on how to register an opt-out from the scheme [8]. A survey of 1400 members of public showed that only 23% remembered receiving the leaflets and 45% did not understand the care.data scheme [9]. Furthermore, a survey of 600 GPs showed that 80% of them also felt that they did not have adequate knowledge of the scheme [9].

In early 2014 the programme faced critical reception from patients and other stakeholders, which was widely reported in the media. This adverse media coverage, and the rise of campaign groups [e.g. 10], prompted a series of damaging disclosures by the NHS relating to past sharing of centralised health records for purposes beyond those relating to improving the public good [11]. The high media profile and widespread criticism of care.data led to a review of the scheme [12], and the UK government postponed the care.data programme in 2014, finally cancelling it in 2016 [13]. A full timeline is shown in Figure 1.

Figure 1: Summary of key events regarding unfolding of care.data throughout 2013-2014 (drawn from Presser et al., 2015 [11]).

<Figure 1 about here>

Following a lengthy consultation [14], a new national strategy for an opt-out model for re-use of NHS patient data was rolled out in May 2018 as the "National Data Opt-Out" [15]. At the current time, future universal data sharing schemes for primary care records under this new strategy are not clear. Given the high likelihood of similar schemes being launched in the near future, it is imperative that

For peer review only

For peer review only - http://bmjopen.bmj.com/site/about/guidelines.xhtml

as many lessons as possible are learned from care.data to promote transparency of such schemes and inclusivity of all stakeholders [16].

Stakeholder reviews on why care.data went wrong have focused on patient and public views

A body of literature has built up which has looked at themes in the public's objections to care.data and why it became so unpopular. These studies have included analyses of citizens' comments on the care.data website and the Guardian newspaper comments section [17], and public views posted on Twitter [8, 18]. Commentators have drawn lessons from public reactions to the care.data scheme to suggest recommendations for future schemes [11], to suggest a theoretical framework for explaining public concern about the scheme [19], and to guide interpretation of new data protection legislation [20].

Why focus on General Practitioners?

Any patient data-sharing scheme requires the support of clinicians as central stakeholders in the project, and as the creators and custodians of patient data. In the United Kingdom, GPs tend to be the legal *owners* of the medical information recorded in their electronic patient records, and in turn are responsible for the way in which these data are processed and used. These responsibilities are enshrined in UK Data Protection Act (the Data Protection Act 1998 at the time of the care.data programme) and the common law duty of confidentiality, and are regulated by the Information Commissioner's Office (ICO).

While the British Medical Association (BMA) was initially in favour of the care.data scheme [21], doctors later wrote publicly about their concerns about the scheme, both before [22] and after it was postponed [23, 24]. It is not clear if opposition to the scheme from GPs was one of the main drivers in the postponement and final failure of the scheme, or if GPs' reservations followed the patient, public and media outcry. To explore this gap in understanding, we conducted a media content analysis of contemporaneous GP reactions to the scheme expressed in the media. The aims of this study were to systematically and comprehensively identify, and qualitatively analyse, newspaper and news website coverage of the care.data debate within the UK, focussing specifically on articles which quoted GPs or other doctors, or were authored by GPs or other doctors, or were written for a solely GP audience. We aimed to describe themes and learning points in doctors' views of the scheme and to look for turning points in opinion during the unfolding events.

For peer review only - http://bmjopen.bmj.com/site/about/guidelines.xhtml

Methods

Selection of sources

A systematic search strategy, following principles outlined in PRISMA reporting [25], was used to identify online media about care.data, which quoted, was written by, or was directed towards UK GPs and other medical doctors during the care.data era. Comments posted by readers in response to articles were not used for analysis.

Mainstream newspapers were searched using the database Nexis; newspapers were included if they had a circulation in print of 100,000 per day or more. The BBC news website was also searched. Through consultation with GPs (n=4) we identified and searched four GP-facing news websites: Pulse Today, GP online, RCGP website and BMA website. The search was constrained to 01/01/2013 to 31/12/2014.

The search terms used in the Nexis database were: “care.data” AND (“doctor” OR “dr” OR “general practitioner” OR “GP” OR “clinician” OR “physician” OR “specialist” OR “professor”).

The search term used in BBC news and on the GP facing news sites was: care.data.

Inclusion and Exclusion Criteria

All articles were screened in full by author YK to identify if they met eligibility criteria (Table 1). For any ambiguity in whether articles met inclusion criteria, authors EF and YK worked together to reach a consensus on inclusion.

Table 1: Inclusion and Exclusion Criteria for Articles

Inclusion Criteria	Exclusion Criteria
Article focussed mainly (>50%) on the care.data project	Less than 50% of the content related to care.data;
And article quoted a GP or doctor expressing an opinion of care.data;	OR they did not include any quotes or discussion about GPs’ or doctors’ views;
OR was authored by a GP or any doctor	OR the article was a short lead-in to a main story elsewhere in the same edition (which also appears in the sample);
OR was written for a majority GP audience	OR it was a summary of, or link to, formal documentation and surveys;
OR discussed the opinions, attitudes or views of doctors about care.data	OR it was not a news article in its own right.

For peer review only

For peer review only - http://bmjopen.bmj.com/site/about/guidelines.xhtml

Data Analysis

A thematic analysis was conducted according to the principles of Braun and Clarke [26] in an iterative process. NVivo v12 software was used which facilitated reading articles and coding themes as they emerged. Key themes were determined according to their relevance to the research questions. Two authors (YK and EF) discussed initial themes, and consolidated emerging and disparate themes into a smaller number of nested themes. The articles were then read again and all quotes linked to a final list of themes. Given the large volume of data, themes are represented using indicative quotes that were the most illustrative for that theme, with attention paid to discordant data.

Results

Search Results

The search returned 923 articles from the Nexis newspaper database (newspapers and web-based publications), British Medical Association (BMA), Pulse and BBC, and after screening against eligibility criteria, 162 were found to meet the inclusion criteria. No relevant articles were returned from the search of 'GPonline' or from the Royal College of General Practitioner's website ('RCGP'). The spread of articles is shown in Table 2.

Table 2: Search Results

Database	No. articles returned by search	No. articles included
Nexis newspaper print articles	451	83
Nexis web-based articles	164	2
BMA	48	16
BBC news	24	8
Pulse	236	53
Total	923	162

Article Characteristics

Characteristics of the articles included for the analysis are summarised in Table 3 and full information given in Appendix 1.

Table 3 Sources of the articles

Source	N Articles	N different authors	N doctors quotes
The Guardian (including the Observer)	8	8	16
The Times	14	6	13

For peer review only

The Telegraph (including Daily Telegraph)	22	8	29
Daily Mail (including Mail Online)	21	8	22
The Independent	12	5	14
London Evening Standard	5	Unknown	9
Pulse Today	53	11	73
BMA	16	Unknown	12
BBC	8	2	8

Thematic Analysis

Thematic analysis revealed five themes regarding GPs' views on care.data. These were:

1. Support for the scheme: care.data would give value for research and may improve care.
2. Concerns about patients lacking informed choice through proposed opt out system.
3. Concerns about legal responsibilities regarding patient data.
4. Concerns about key safeguards – GPs were unable to reassure patients that they would not be re-identified and their data would not be sold onwards.
5. Concerns about trust between patients and doctors.

Quotes presented below can be cross-referenced with Supplementary File 1 .

1. Support for the scheme: Care.data would give value for research and may improve care.

Early in the timeline, many GPs were positive about the potential benefits of care.data. The scheme was supported by the RCGP with Dr Clare Gerada, former president of the College, expressing support and committing to work with the BMA and NHS England on the care.data project (5, BMA, May 2013). Dr David Davies, a GP and the HSCIC's executive medical director, believed use of linked data would help improve services and allow targeted interventions, by determining patients at higher risks of a particular disease (1, Pulse Today, Mar 2013). Dr Davies explained:

> *'We'll be linking data, doing risk profiles on the data then feeding it back to GPs to adapt their care. That'll be OK because we're feeding data back to the people who gave it to us for it to be used in direct patient care' (1, Pulse Today, Mar 2013).*

Professor Liam Smeeth, a practising GP and professor in London highlighted the necessity of using patients' data to run the NHS (32, Pulse Today, Jan 2014):

> *'These are data that are absolutely necessary to keep the NHS up with the times, to develop new inventions, or to be safe and effective, so we know what's going on in the health service. …There are people worried about confidentiality but I am hoping there aren't people sitting*

For peer review only - http://bmjopen.bmj.com/site/about/guidelines.xhtml

around saying we don't need to do this because we don't need this data' (32, Pulse Today, Jan 2014).

Dr Francesca Lasman, a GP in Cambridgeshire believed the care.data scheme would lead to comprehensive and meaningful analysis leading to insights on managing comorbidities in general practice (39, Pulse Today, Jan 2014). She said:

'I think it is vital to develop an understanding of how best to manage complex co-morbidities which exist in general practice. Drug companies and studies focus on one problem but real data from people with multiple conditions and on many medications gives at least a chance of some meaningful analysis and a start for the best approach to tackling prevention and treatments' (39, Pulse Today, Jan 2014).

A number of medical charities expressed support for the care.data scheme in line with NHS England's public awareness campaign. Doctors working for these charities warned the public that if too many people opted out of the scheme, it would impact on science which could lead to increased numbers of deaths in the population (32, Pulse Today, Jan 2014). Professor Peter Weissberg of the British Heart Foundation explained how lack of data could lead to delayed discoveries and more deaths:

'We can carry on making [discoveries] without [it], but we will make them extraordinarily slowly and people will die in the interim. Not only will they die as a consequence of habits and behaviours they have, which we haven't understood properly, [but] they will die as a consequence of well-meaning attempts by doctors… [who are] treating patients on a daily basis on what we call the evidence base' (32, Pulse Today, Jan 2014).

This generalised support for the scheme was not without qualification, however. The BMA greatly emphasised the importance of gaining public trust and confidence in the scheme (72, Pulse Today, Jan 2014). Dr Chaand Nagpaul, the chair of the BMA said:

'The BMA continues to support the principle of using anonymised data to plan and improve the quality of NHS care for patients. However, this must be done with the support and consent of the public, therefore patients must be made aware of what is happening to their personal information, what the proposals mean and what their rights are if they do not wish their data to be extracted' (72, Pulse Today, Jan 2014).

For peer review only - http://bmjopen.bmj.com/site/about/guidelines.xhtml

2. Concerns about patients lacking informed choice with the proposed opt out system

Doctors were concerned about the care.data opt-out mechanism, and that patients were not well informed enough to make a choice. In August 2013, GPs were given the responsibility of informing their patients of their medical data extraction for care.data during the 8 weeks before the extraction started. However, GPs were not provided extra funding to carry out this task, leading to frustration (154, Pulse Today, Aug 2014). Dr Grant Ingrams, a practising GP in Warwickshire and deputy chair of the General Practitioners Committee's (GPC's) IT subcommittee, told Pulse:

'…I don't think any GP is going to pay hundreds or thousands out of their own pocket to run a campaign' (154, Pulse today, Aug 2014).

A survey of 427 GPs carried out by Pulse revealed that only 19% agreed with the opt-out system with 75% supporting a change to an opt-in system. Ninety percent of GPs stated that if an opt-out system were used, NHS England should provide an opt-out form with the "Better Information Means Better Care" leaflets. The GPs expressed concerns that having to opt out could be challenging for particular groups within society, for example migrants (111, Pulse Today, Feb 2014).

RCGP honorary secretary, Professor Nigel Mathers was outspoken about the public's lack of awareness of the scheme and of their right to opt out. He criticised the NHS awareness campaign by stating:

"Crucially, where a scheme is based on an opt-out approach, such as in the case of care.data, we believe that it is vital that the NHS is able to show that it is beyond reproach in having done everything practically possible to ensure that patients and the public know about their right to opt out prior to it going ahead" (82, The Telegraph, Feb 2014).

Prof Mathers also criticised the leaflet scheme, and announced that since it had not worked, all other possible means of communication such as radio, national TV and online adverts should be used. He recommended that personalised letters should be sent to all households in England (79, Mail Online, Feb 2014). Prof Mathers said:

'At present, we are concerned that levels of awareness concerning care.data are very low, and believe that there is a strong case for substantial additional activity over and above that already in place to tackle this' (79, Mail Online, Feb 2014).

For peer review only - http://bmjopen.bmj.com/site/about/guidelines.xhtml

Following the postponement of the scheme, GPs were surveyed on their opinions. Some GPs believed patients' data should only be extracted with their explicit consent and an opt-in system was the only right way it could be done (143, Pulse Today, May 2014). Some GPs went further and decided to opt out all their patients unless they specifically requested to opt in (141, Pulse Today, May 2014). An Oxfordshire GP who had been practising medicine for 40 years had decided to opt out his entire practice due to lack of information about the scheme. Dr Gancz felt "...people are being bulldozed into giving consent by default" (53, The Telegraph, Feb 2014).

A number of GPs who had been in favour of the care.data project initially, later stated that they did not approve of the way the government blamed the lack of public support on GPs (153, Pulse Today, Aug 2014). A GP from West Sussex, Dr Jeremy Luke, said:

> *'... [The government] has, as usual, tried to blame GPs for their own failure to engage with and listen to the views of patients' (153, Pulse Today, Aug 2014).*

In contrast, some GPs supported an opt-out system and believed an opt-in system would be a retrograde step in research. Dorset GP L-J Evans explained that an opt-in system would rely on every patient consenting for their medical data to be used, which did not work in her opinion. She explained that a disadvantage of the opt-in system would be skewed results and less robust research (150, BMA, Jun 2014).

3. Concerns about legal responsibilities regarding patient data

GPs were required to share their patients' data for care.data by the 2012 Health and Social Care Act (HSCA) which put them in a legally challenging situation, as this sharing might have breached the common law duty of confidentiality. Doctors' duty of confidence regarding the information they hold about a patient can only be lifted where the patient has an expectation of a secondary usage [27], in this case, to be shared to a centralised NHS data warehouse. GPs perceived that, legally, they could therefore only share data if they were sure that patients were aware of, and fully understood, the care.data scheme and their rights to opt out (111, Pulse Today, Feb 2014). One response to this concern was that some GPs opted out their entire registered list of patients, even though this led them to be in breach of contract with the 2012 HSCA. A number of doctors who opted out their entire practice believed that in allowing patients' data to be extracted without patients being fully informed, the doctors would not be doing their duty to protect patients' data and would be in breach of the Data Protection Act (19, Pulse Today, Nov 2013).

For peer review only - http://bmjopen.bmj.com/site/about/guidelines.xhtml

The GPC deputy chair warned GPs that opting out entire practices would be ‘breaking the law’, as GPs had a ‘statutory duty’ according to the Health and Social Care Act to share their patients’ data (21, Pulse Today, Nov 2013). However, GPs believed that if patients complained about not being aware of their data being shared, GPs would be held accountable (as they were data controllers) and not the government (33, Pulse Today, Jan 2014). Dr Grant Ingrams, who was a former chair of the GPC’s IT subcommittee and a GP in Warwickshire, explained how he thought GPs were ‘confounded’ between these conflicting Acts (6, Pulse Today, Aug 2013):

> *‘…Practices have a lawful obligation under the Health and Social Care Act to send the data to the HSCIC. But an obligation under the Data Protection Act to protect patients’ data. It’s leaving practices confounded between two rights. If practices aren’t sued by one, they’ll be sued by another’ (6, Pulse Today, Aug 2013).*

Later on, a survey indicated that 8% of GPs would opt out all their patients, despite being aware they would face a legal challenge and the possibility of their contract being terminated (111, Pulse Today, Feb 2014).

4. Concerns about key safeguards – GPs were unable to reassure patients that they would not be re-identified and their data would not be sold onwards.

From the launch of care.data, there had been issues about a lack of transparency and lack of assurance about the confidentiality of data held by HSCIC (139, BMA, Apr 2014; 150, BMA, Jun 2014). One of doctors’ main concerns was the possibility of patients being re-identified from their pseudonymised records, this was expressed before the project was even launched (9, Pulse Today, Sep 2013). Data extracted would contain information about the patients’ prescriptions, investigation results and some diagnostic codes. However, more sensitive codes such as IVF, abuse, HIV, termination of pregnancy, convictions or STIs would not be extracted from surgeries. Patient identifiers such as name and date of birth would not be extracted but patients’ unique NHS identification number would be. GPs identified that there was a small risk of identification for patients with rare diseases (2, Pulse Today, Apr 2013).

NHS England stated that the data extracted would be crucial to integrate care and reduce treatment and care inequalities. Despite guarantees made by the government, doctors were not reassured (2, Pulse Today, Apr 2013). A GP working in Warwickshire, Dr Paul Thornton stated his concern very early in the conception of the scheme:

For peer review only - http://bmjopen.bmj.com/site/about/guidelines.xhtml

> *…'lots of people will want [the data, including] other Government bodies, the Department for Work and Pensions, financial institutions, pharmaceutical companies' (2, Pulse Today, Apr 2013).*

A survey carried out by Pulse revealed that approximately 40% of GPs wished to opt out of the system themselves, as they believed they could potentially be re-identified. An anonymous respondent stated they 'cannot see any clinical justification for the identifiable data extraction' (23, Pulse Today, Dec 2013).

Dr Marie-Louise Tidmarsh, a GP in Derbyshire, worried about being identified by her neighbours and as a result decided to opt out of the system (39, Pulse Today, Jan 2014). She said:

> *'I think patients have been misled about the "confidential" nature of the data extractions, and it is not clear to whom the data may be sold' (39, Pulse Today, Jan 2014).*

Moreover, worries about the risk of a substantial database like care.data being hacked was expressed by some doctors (54, Daily Mail, Feb 2014). This risk of hacking or re-identification was perceived to increase with the fact that unknown, and possibly profit-motivated, end-users would be provided with the data. GPs were concerned about likelihood of the data being sold to private companies (51, Daily Mail, Feb 2014).

Professor Sir Brian Jarman, a "*world specialist on hospital data*", claimed that insurance companies could identify patients in less than two hours. He explained that tracking down individuals and cross-referencing information could be fairly easy as many of these companies are already in possession of patients' names and home addresses (70, Mail Online, Feb 2014). Sir Brian said:

> *'I've spoken to analysts who say they can match individual people within a couple of hours. Many organisations, such as insurance companies, hold details of people that include not only name but also postcode, date of birth and gender, which would make it possible to identify named individuals in postcodes and thus have access to their confidential medical information in the care.data database. There is simply too much data and the risks that something leaks are too great' (70, Mail Online, Feb 2014).*

A GP in Kent, Dr Ian Williams, indicated he did not believe proposed measures would mask his identity in the data:

> *'I wish to opt out as I am concerned about identifiable data being moved around and passed on to third parties. I would have no objection if the data held on my records was*

For peer review only

"pseudonymised" before being extracted from the practice records' (39, Pulse Today, Jan 2014).

5. Concerns about trust between patients and doctors

GPs believed that historically, patients have trusted doctors to look after their personal data and make sure of their safety (71, London Evening Standard, Feb 2014). The doctors' relationship with their patients had always been based on trust and patients did not expect any information they share during consultations to end up in the government's database without their knowledge (25, The Independent, Jan 2014).

The fear of losing their patients' trust was one of GPs' main concerns about care.data. The BMA representatives explained that if patients were not assured of the security of their medical data, it would lead to mistrust and lack of communication by patients. This would mean doctors may not have all the information they need about the patient in the future, and patients would not share all the symptoms that are required to reach a correct diagnosis (112, BMA, Feb 2014; 100, The Daily Telegraph, Feb 2014). The Chair of the Family Doctor Association, Dr Peter Swinyard, explained:

> *"We have a very basic principle that whatever our patients tell us is confidential. We are completely hamstrung if patients feel they can't tell us something that - in many cases - they wouldn't tell anyone else. It is this threat to total confidentiality at consultations that gets up most GPs' noses" (37, The Independent, Jan 2014).*

Professor Sue White of Birmingham University stated this problem could affect particularly vulnerable groups within society, for example women who are experiencing domestic violence issues. She believed that these women are already reluctant to open up to their GPs about their difficulties and if they doubted their data was completely confidential it might make them avoid visiting their GPs (22, The Observer, Nov 2013).

Nested within this theme was the additional distrust between doctors and the government. Because GPs did not trust the government to use patients' data safely, they could not endorse the scheme and this compounded their fear of losing their patients' trust. Dr Philip Bolitho-Jones, a GP partner in Hertfordshire, stated he did not trust the politicians to decide what happened to his or his patients' medical data (141, Pulse Today, May 2014).

> *'We don't trust the politicians; they've got no idea what they're doing. They've already leaked before the system was even set up they were leaking data left right and centre. I*

For peer review only - http://bmjopen.bmj.com/site/about/guidelines.xhtml

wouldn't trust them with my notes; I wouldn't trust them with any of our patients' notes' (141, Pulse Today, May 2014).

Discussion

Our results showed that GPs supported the care.data scheme in theory at the outset, and agreed that data extraction and re-use was necessary or beneficial for good research, for improving care and for saving lives. However, as time went on, they increasingly opposed certain aspects of implementation of the programme and articulated a number of key concerns. GPs cared about their patients being properly informed about the scheme. They felt the government had not informed patients adequately and could not be trusted to keep patients' data safe. They perceived that the onus was being put on them to make sure their patients were well informed, but no financial or time resources were given to them to achieve this. They disliked being scapegoated for the project's failures in communication. They also perceived that the lack of communication and guidance left them conflicted by competing obligations to share data whilst maintaining their duty of confidentiality. GPs were fearful that they could end up being held accountable under one or other of these duties.

GPs articulated a conflict over the consent mechanism in the care.data scheme. They felt that an opt-out system would be challenging for some vulnerable groups (e.g. migrants, trans-people [28]), but understood that an opt-in system would introduce bias, and potentially reduce the quality of research. They also expressed distrust in the system proposed for protecting patient identities within the scheme, this distrust was particularly focussed on the government's history of mishandling sensitive records, and the possibility of them being sold to private companies such as insurance companies. A substantial proportion of GPs indicated they did not want their own data to be extracted, because they believed patients could be quite quickly re-identified by private companies with a profit motive. Finally, GPs feared that if patients felt their data were not confidential, patients would not tell them important medical issues, and this would impede GPs' ability to provide good clinical care, and risk harming the most vulnerable patient groups.

Many of the themes expressed by GPs at the time of the care.data scheme tally with those expressed by other stakeholders, about this scheme in particular, and about patient data sharing in general. Citizens writing comments on the care.data and Guardian websites, and publishing micro-blogs on Twitter, complained of a lack of transparency and poor communication about the scheme, a lack of respect for confidentiality, misgivings about the opt-out mechanism, and fears of erosion of

For peer review only - http://bmjopen.bmj.com/site/about/guidelines.xhtml

trust in GPs and the NHS [8, 17]. They were also concerned about commercial uses of data. Citizens could see the potential benefits of the scheme and felt that if the societal benefits and "common good" that could be derived from the scheme were made a central tenet of the scheme, [17], and meaningful public and patient involvement could be achieved [8], the scheme would have been more likely to have been accepted. Reviews of patient opinions on a range of data-sharing schemes [29, 30] have also identified similar themes underlying lay thinking on the topic. The majority of respondents to surveys and qualitative studies indicate high but qualified support of sharing patient data for research and other secondary purposes. Participants in these studies articulated fears that their privacy may be compromised and expressed distrust of particular organisations, particular those with a profit motive, which may have access to the data.

Our study additionally exposed some GP-specific issues around the implementation of care.data. GPs felt under-resourced to meet their competing duties under the HSCA 2012 and the DPA 1998. To meet their duties under these two acts, they needed to make sure patients were fully informed, but were unable to do this without any additional resource coming from the government. They also feared that data sharing schemes would detrimentally affect their relationships with their patients, leading to an erosion in trust, and leading to them being less able to care for their patients effectively.

Strengths and limitations of this study

Our approach of analysing contemporary GPs comments on care.data in published newspaper and online news articles made sure we accessed the voice of doctors during events of the unfolding crisis, and not with the hindsight of knowledge of how the situation has evolved since. The search strategy used was comprehensive and designed to include all the mainstream articles written by, quoting or written for doctors about care.data. By following a systematic approach, we minimised bias in the selection of articles for the study. However, it is possible that other sources of news, read by or authored by GPs, were not captured here, or that different opinions were being widely shared through informal GP networks and escaped our analysis. Additionally, those doctors contributing to mainstream news articles may not be representative of GPs in general, but instead can be viewed as key contributors to the prominent circulating discourses at the time. We acknowledge that the discourses of the quoted GPs, which were expressed in 2013-2014, may not represent their current views on the care.data scheme, its subsequent failure, or on future schemes with similar aims.

The thematic analysis was conducted by one researcher and checked carefully by a second, which may make the results more subjective and less reproducible than if a team approach had been taken. However, the themes found in our study selected map closely onto key themes found in other

For peer review only - http://bmjopen.bmj.com/site/about/guidelines.xhtml

work in this field, suggesting the results reported are at least consistent with stakeholder views from other sources.

Conclusions and recommendations

The study suggests that, should a similar programme be introduced in future, the support of GPs should be obtained before implementation in order to improve the success of the programme. GPs' concerns regarding confidentiality, patient information, informed consent, patient trust, need for resources, and conflicting legal obligations should be addressed during the planning phase of any data-sharing scheme, and reflected in the implementation of the programme. As key stakeholders in any primary care data re-use, GPs should be meaningfully involved at all aspects of such programmes, such as inviting GP representatives to sit within teams that design and implement such schemes. We would recommend a co-design approach which includes all stakeholders from the outset. This was the case, for example, in the development of the subsequent National Data Opt-Out where the opinions of key stakeholders and medical bodies including doctors were considered in planning the scheme. There was effective communication with the RCGP and the Royal Colleges of Nursing and Midwifery to ensure the NHS workforce is ready across the country to start the scheme. Examples of such good practice should be studied further, and can be combined with the results of our study, to ensure a social licence for patient data sharing is achieved with patient and clinicians, before the start of any future scheme.

Ethics approval and consent to participate

This study used publically available data from mainstream media. These data are already in the public domain. No ethical approvals were sought.

Consent for publication

This study used publically available data from mainstream media. These data are already in the public domain. No consent was sought.

Patient and Public Involvement

No patient involved.

Availability of data and materials'

This study used publically available data from main stream media. These data are already in the public domain.

For peer review only - http://bmjopen.bmj.com/site/about/guidelines.xhtml

Authors' contributions

EF, AB and SVK conceived of the presented idea. YK performed the searches, screening, data extraction and analysis with supervision from EF. MC aided interpretation of the results. EF and YK drafted the manuscript. All authors discussed results, contributed to the manuscript and approved the final version.

References

1. Hoeksma J: **The NHS's care.data scheme: what are the risks to privacy?** *BMJ : British Medical Journal* 2014, **348**:g1547.
2. **Hospital Episode Statistics (HES)** [https://digital.nhs.uk/data-and-information/data-tools-and-services/data-services/hospital-episode-statistics]
3. Herrett E, Gallagher AM, Bhaskaran K, Forbes H, Mathur R, van Staa T, Smeeth L: **Data resource profile: clinical practice research datalink (CPRD)**. *International Journal of Epidemiology* 2015, **44**(3):827-836.
4. Lewis JD, Schinnar R, Bilker WB, Wang X, Strom BL: **Validation studies of the health improvement network (THIN) database for pharmacoepidemiology research**. *Pharmacoepidemiology and Drug Safety* 2007, **16**(4):393-401.
5. Hippisley-Cox J, Stables D, Pringle M: **QRESEARCH: a new general practice database for research**. *Journal of Innovation in Health Informatics* 2004, **12**(1):49-50.
6. **Better information means better care** [https://www.england.nhs.uk/wp-content/uploads/2014/01/cd-leaflet-01-14.pdf]
7. **Care.data campaign leaflet slammed** [https://www.digitalhealth.net/2014/01/care-data-campaign-leaflet-slammed/]
8. Hays R, Daker-White G: **The care. data consensus? A qualitative analysis of opinions expressed on Twitter**. *BMC Public Health* 2015, **15**(1):838.
9. **MPS surveys of GPs and public reveal lack of information around care data** [https://www.medicalprotection.org/uk/articles/mps-surveys-of-gps-and-public-reveal-lack-of-information-around-care-data]
10. **Med Confidential** [https://medconfidential.org/about/]
11. Presser L, Hruskova M, Rowbottom H, Kancir J: **Care. data and access to UK health records: patient privacy and public trust**. *Technology Science* 2015, **2015081103**:1-35.
12. Partridge N: **Data Release Review**. In.: Health and Social Care Information Centre; 2014.
13. **NHS England to close care.data programme following Caldicott Review** [http://www.nationalhealthexecutive.com/Health-Care-News/nhs-england-to-close-caredata-programme-following-caldicott-review]
14. **Review of Data Security, Consent and Opt-Outs** [https://assets.publishing.service.gov.uk/government/uploads/system/uploads/attachment_data/file/535024/data-security-review.PDF]
15. **National data opt-out** [https://digital.nhs.uk/services/national-data-opt-out-programme]
16. Ford E, Boyd A, Bowles JKF, Havard A, Aldridge RW, Curcin V, Greiver M, Harron K, Katikireddi V, Rodgers SE *et al*: **Our data, our society, our health: A vision for inclusive and transparent health data science in the United Kingdom and beyond**. *Learning Health Systems* 2019, **3**(3):e10191.
17. Sterckx S, Rakic V, Cockbain J, Borry P: **"You hoped we would sleep walk into accepting the collection of our data": controversies surrounding the UK care. data scheme and their wider relevance for biomedical research**. *Medicine, Health Care and Philosophy* 2016, **19**(2):177-190.

For peer review only - http://bmjopen.bmj.com/site/about/guidelines.xhtml

18. Amor BR, Vuik SI, Callahan R, Darzi A, Yaliraki SN, Barahona M: **Community detection and role identification in directed networks: understanding the twitter network of the care. data debate**. In: *Dynamic Networks and Cyber-Security.* World Scientific; 2016: 111-136.

19. Carter P, Laurie GT, Dixon-Woods M: **The social licence for research: why care. data ran into trouble**. *Journal of Medical Ethics* 2015, **41**(5):404-409.

20. Sorbie A: **Sharing confidential health data for research purposes in the UK: where are 'publics' in the public interest?** *Evidence & Policy: A Journal of Research, Debate and Practice* 2019.

21. **Care.data joint statement** [https://www.nelsonmedicalpractice.co.uk/wp-content/uploads/2015/03/caredatajointstatement1.pdf]

22. **Care.data confidentiality concerns cannot be ignored, say doctors** [https://www.bma.org.uk/news/2014/march/caredata-confidentiality-concerns-cannot-be-ignored-say-doctors]

23. **Data-sharing programme must include 'the profession from the outset'** [https://www.bma.org.uk/news/2016/july/data-sharing-programme-must-include-the-profession-from-the-outset]

24. **Make care.data opt in or we will opt patients out, say GPs** [http://www.pulsetoday.co.uk/news/gp-topics/it/make-caredata-opt-in-or-we-will-opt-patients-out-say-gps/20007472.article]

25. Moher D, Liberati A, Tetzlaff J, Altman DG: **Preferred reporting items for systematic reviews and meta-analyses: the PRISMA statement**. *Annals of Internal Medicine* 2009, **151**(4):264-269.

26. Braun V, Clarke V: **Using thematic analysis in psychology**. *Qualitative Research in Psychology* 2006, **3**(2):77-101.

27. **MRC Ethics Series. Using Information about People in Health Research** [https://mrc.ukri.org/documents/pdf/personal-information-in-medical-research/]

28. Thomson RM, Katikireddi SV: **Improving the health of trans people: the need for good data**. *The Lancet Public Health* 2019, **4**(8):e369-e370.

29. Stockdale J, Cassell J, Ford E: **"Giving something back": A systematic review and ethical enquiry into public views on the use of patient data for research in the United Kingdom and the Republic of Ireland**. *Wellcome Open Res* 2019, **3**:6-6.

30. Aitken M, Jorre JdS, Pagliari C, Jepson R, Cunningham-Burley S: **Public responses to the sharing and linkage of health data for research purposes: a systematic review and thematic synthesis of qualitative studies**. *BMC Medical Ethics* 2016, **17**(1):73.

For peer review only

For peer review only - http://bmjopen.bmj.com/site/about/guidelines.xhtml

For peer review only

For peer review only - http://bmjopen.bmj.com/site/about/guidelines.xhtml

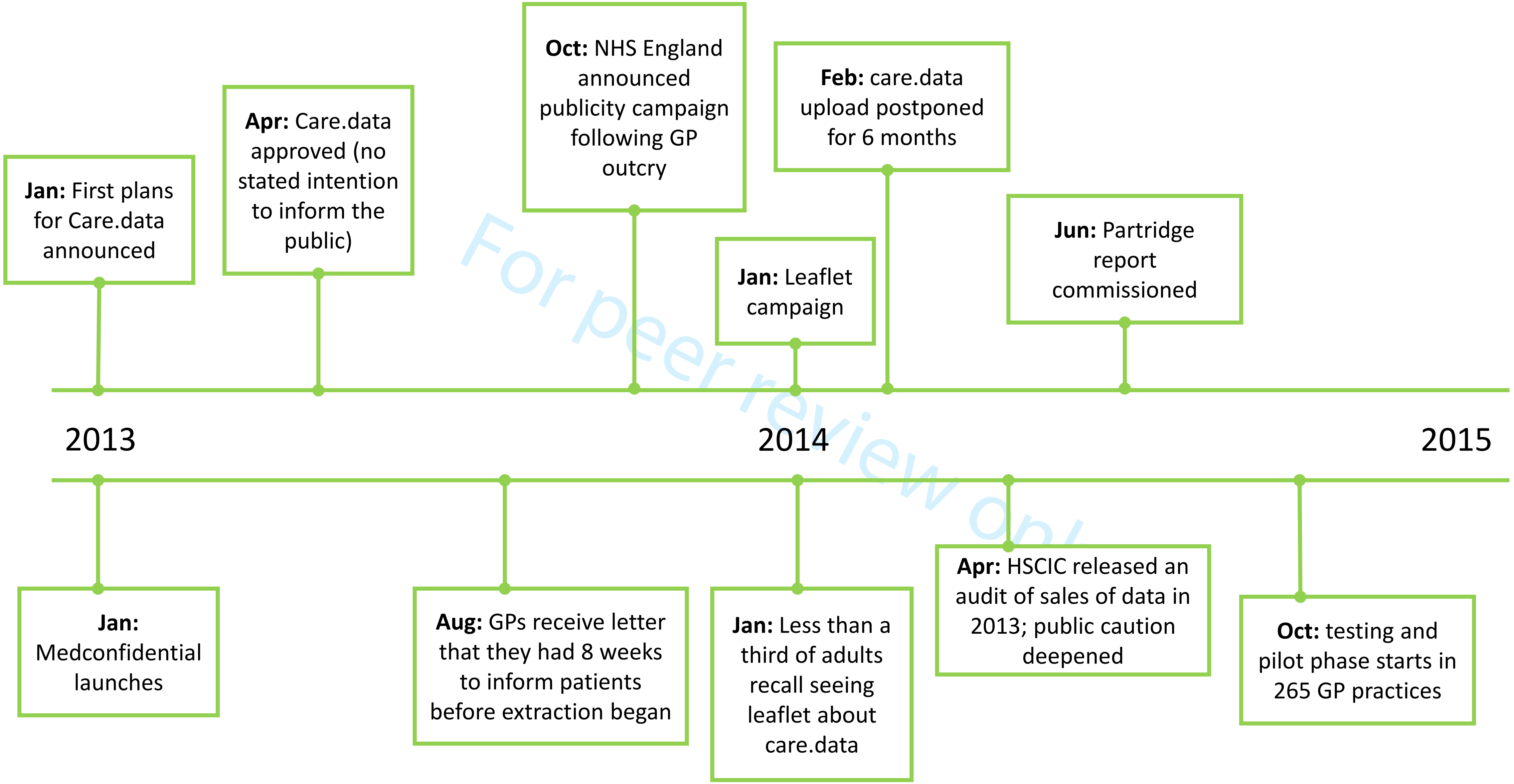

For peer review only - http://bmjopen.bmj.com/site/about/guidelines.xhtml

Appendix 1: All articles used in analysis

Article number	Source type	Source name	Article title	URL	Author's name	Date
1	GP source	Pulse Today	NHS to link up data from GP records and secondary care	http://www.pulsetoday.co.uk/news/gp-topics/it/nhs-to-link-up-data-from-gp-records-and-secondary-care/20002260.article	Madlen Davies	15/03/2013
2	GP source	Pulse Today	Risk patients will be identified by anonymised GP record data, NHS England admits	http://www.pulsetoday.co.uk/news/gp-topics/it/risk-patients-will-be-identified-by-anonymised-gp-record-data-nhs-england-admits/20002593.article	Madlen Davies	12/04/2013
3	GP source	Pulse Today	Patients to be given 'veto' over their data being shared from GP records	http://www.pulsetoday.co.uk/news/gp-topics/it/patients-to-be-given-veto-over-their-data-being-shared-from-gp-records/20002754.article	Madlen Davies	26/04/2013
4	Newspaper	The Telegraph	Database of patient information takes first steps;<br>Confidential information from medical records held by family GPs will begin to be entered into a central database from next month.	https://www.telegraph.co.uk/news/health/news/10087957/Database-of-patient-information-takes-first-steps.html	Laura Donnelly	29/05/2013
5	GP source	BMA	Information sharing guidance offered to GPs	https://www.bma.org.uk/news/2013/may/information-sharing-guidance-offered-to-gps	Not stated	29/05/2013
6	GP source	Pulse Today	GPs face prosecution unless they inform patients their data could be used outside the NHS	http://www.pulsetoday.co.uk/news/gp-topics/it/gps-face-prosecution-unless-they-inform-patients-their-data-could-be-used-outside-the-nhs/20003860.article	Madlen Davies	05/08/2013

7	GP source	Pulse Today	Private companies set for access to patient data for just £1	http://www.pulsetoday.co.uk/news/gp-topics/it/private-companies-set-for-access-to-patient-data-for-just-1/20003879.article	Madlen Davies	09/08/2013
8	GP source	BMA	New rules for doctors ensuring data confidentiality	https://www.bma.org.uk/news/2013/september/new-rules-for-doctors-ensuring-data-confidentiality	Not stated	13/09/2013
9	GP source	Pulse Today	GP leaders consider boycott of NHS England's data extraction programme	http://www.pulsetoday.co.uk/home/finance-and-practice-life-news/gp-leaders-consider-boycott-of-nhs-englands-data-extraction-programme/20004354.article	Madlen Davies	19/09/2013
10	Newspaper	Mail Online	GPs threaten to boycott 'Big Brother' NHS database which would force them to send confidential patient records to private firms	https://www.dailymail.co.uk/news/article-2428211/GPs-threaten-boycott-Big-Brother-NHS-database.html	Stephen Adams	22/09/2013
11	GP source	Pulse Today	GPs unable to object to patient data being shared, warns GPC	http://www.pulsetoday.co.uk/news/gp-topics/it/gps-unable-to-object-to-patient-data-being-shared-warns-gpc/20004458.article	Madlen Davies	26/09/2013
12	GP source	Pulse Today	NHS data extraction programme poses 'enormous threat' to privacy	http://www.pulsetoday.co.uk/news/gp-topics/it/nhs-data-extraction-programme-poses-enormous-threat-to-privacy/20004575.article	Madlen Davies	01/10/2013
13	GP source	Pulse Today	Eight weeks to inform patients their data is going to be harvested, GPs warned	http://www.pulsetoday.co.uk/news/gp-topics/it/eight-weeks-to-inform-patients-their-data-is-going-to-be-harvested-gps-warned/20004562.article	Madlen Davies	04/10/2013

14	Newspaper	The Independent	Care.data project to collate all NHS patients' records	https://www.independent.co.uk/life-style/health-and-families/health-news/caredata-project-to-collate-all-nhs-patients-records-8882379.html	Charlie Cooper	16/10/2013
15	GP source	Pulse Today	NHS England bows to confidentiality concerns and launches £2m national publicity campaign on care.data	http://www.pulsetoday.co.uk/nhs-england-bows-to-confidentiality-concerns-and-launches-2m-national-publicity-campaign-on-caredata/20004748.article	Sofia Lind	16/10/2013
16	GP source	BMA	GPs back patient-data awareness campaign	https://www.bma.org.uk/news/2013/october/gps-back-patient-data-awareness-campaign	Not stated	16/10/2013
17	Newspaper	Mail Online	NHS climbdown over 'big brother' database after the Mail on Sunday highlighted serious concerns over the plan	https://www.dailymail.co.uk/news/article-2468175/NHS-climbdown-big-brother-database-Mail-Sunday-highlighted-concerns-plan.html	Stephen Adams	19/10/2013
18	GP source	Pulse Today	Big data = big deal	http://www.pulsetoday.co.uk/views/blogs/big-data-big-deal/20004796.blog	Dr Hadrian Moss	23/10/2013
19	GP source	Pulse Today	Why I'm opting patients out of the care.data scheme	http://www.pulsetoday.co.uk/your-practice/comment/why-im-opting-patients-out-of-the-caredata-scheme/20005022.article	By one anonymous doctor	12/11/2013
20	GP source	Pulse Today	GP takes 'unlawful' decision to opt patients out of care.data programme	http://www.pulsetoday.co.uk/news/gp-topics/it/gp-takes-unlawful-decision-to-opt-patients-out-of-caredata-programme/20005018.article	Alex Matthews-King	12/11/2013

21	GP source	Pulse Today	Second GP decides to opt all patients out of records extraction as care.data rebellion grows	http://www.pulsetoday.co.uk/news/gp-topics/it/second-gp-decides-to-opt-all-patients-out-of-records-extraction-as-caredata-rebellion-grows/20005097.article	One anonymous doctor	19/11/2013
22	Newspaper	The Observer	Fears grow over open access to patient records: Confidential information could be sold to the private sector	https://www.theguardian.com/society/2013/nov/24/fears-grow-open-access-patient-records	Paul Dinsdale	24/11/2013
23	GP source	Pulse Today	Biggest risk to care.data scheme is potential loss of GP 'confidence' in benefits of scheme	http://www.pulsetoday.co.uk/news/gp-topics/it/biggest-risk-to-caredata-scheme-is-potential-loss-of-gp-confidence-in-benefits-of-scheme/20005223.article	Not stated	13/12/2013
24	GP source	BMA	Awareness of patient data sharing drive begins	https://www.bma.org.uk/news/2014/january/awareness-of-patient-data-sharing-drive-begins	Not stated	06/01/2014
25	Newspaper	The Independent	Your life in their hands: is the care.data NHS database a healthy step or a gross invasion of patient privacy?;<br>Doctors are worried that centralising our most personal records will break the bond of trust that underpins the NHS	https://www.independent.co.uk/life-style/health-and-families/health-news/your-life-in-their-hands-is-the-caredata-nhs-database-a-healthy-step-or-a-gross-invasion-of-patient-9042564.html	Charlie Cooper	06/01/2014
26	GP source	Pulse Today	Care.data - every little helps	http://www.pulsetoday.co.uk/views/blogs/caredata-every-little-helps/20005487.blog	Dr Hadrian Moss	08/01/2014
27	GP source	Pulse Today	GPs held responsible for patient complaints over NHS data-sharing project, says ICO	http://www.pulsetoday.co.uk/news/gp-topics/it/gps-held-responsible-for-patient-complaints-over-nhs-data-sharing-project-says-ico/20005505.article	Alex Matthews-King	10/01/2014

For peer review only - http://bmjopen.bmj.com/site/about/guidelines.xhtml

28	Newspaper	The Daily Telegraph	Patients urged not to opt out of health database	Not available to the public	Sarah Knapton	13/01/2014
29	Newspaper	London Evening Standard	NHS data move opt-out 'damaging'	Not available to the public	Not stated	13/01/2014
30	Newspaper	The Telegraph	Health records of every NHS patient to be shared in vast database;<br>The health records of everyone in the NHS will be pooled in a vast database which can be accessed by researchers and pharmaceutical companies. But campaigners warn it could breach privacy.	https://www.telegraph.co.uk/news/10565160/Health-records-of-every-NHS-patient-to-be-shared-in-vast-database.html	Sarah Knapton	13/01/2014
31	Web publication	Sky News	NHS Data-Sharing Scheme 'Vital To Service'	https://news.sky.com/story/nhs-data-sharing-scheme-vital-to-service-10421691	Not stated	13/01/2014
32	GP source	Pulse Today	Scientists launch campaign to discourage patients from opting out of care.data	http://www.pulsetoday.co.uk/news/scientists-launch-campaign-to-discourage-patients-from-opting-out-of-caredata/20005523.article	Alex Matthews-King	13/01/2014
33	GP source	Pulse Today	Revealed: Independent experts overseeing care.data have approved 31 releases of identifiable patient data since April	http://www.pulsetoday.co.uk/news/gp-topics/it/revealed-independent-experts-overseeing-caredata-have-approved-31-releases-of-identifiable-patient-data-since-april/20005572.article	Alex Matthews-King	17/01/2014
34	Newspaper	The Guardian	Patient records to be sold from NHS database: Fears anonymous health data could be matched to insurers' medical files	Not available to the public	Randeep Rames	20/01/2014
35	Newspaper	The Daily Telegraph	Don't mislead us about our medical records; The NHS is asking us to hand over our private data - yet it cannot guarantee anonymity	https://www.telegraph.co.uk/news/nhs/10584402/Dont-mislead-us-about-our-NHS-medical-records.html	Philip Johnston	21/01/2014

36	GP source	Pulse Today	MP tables motion to halt care.data rollout as 2,400 patients call helpline	http://www.pulsetoday.co.uk/news/gp-topics/it/mp-tables-motion-to-halt-caredata-rollout-as-2400-patients-call-helpline/20005621.article	Alex Matthews-King	22/01/2014
37	Newspaper	The Independent	40 per cent of GPs plan to opt out of the NHS big data sweep, due to a lack of confidence in the project	https://www.independent.co.uk/life-style/health-and-families/health-news/40-per-cent-of-gps-plan-to-opt-out-of-the-nhs-big-data-sweep-due-to-a-lack-of-confidence-in-the-9083806.html	Charlie Cooper	24/01/2014
38	Newspaper	The Telegraph	Four in 10 GPs to opt out of NHS database; Family doctors raise concerns about new plans to share patients data	https://www.telegraph.co.uk/news/health/news/10595743/Four-in-10-GPs-to-opt-out-of-NHS-database.html	Laura Donnelly	24/01/2014
39	GP source	Pulse Today	Over 40% of GPs intend to opt themselves out of care.data scheme	http://www.pulsetoday.co.uk/news/gp-topics/it/over-40-of-gps-intend-to-opt-themselves-out-of-caredata-scheme/20005648.article	Nigel Praities	24/01/2014
40	Newspaper	i-Independent Print Ltd	GPs worried about confidentiality of patient data scheme;<br>HEALTH	Not available to the public	Charlie Cooper	25/01/2014
41	Media	BBC	Are your medical records in danger?	https://www.bbc.co.uk/news/health-25919399	Nick Triggle	28/01/2014
42	GP source	Pulse Today	Dr Amir Hannan: 'This goes to the very heart of the doctor-patient relationship'	http://www.pulsetoday.co.uk/news/gp-topics/it/dr-amir-hannan-this-goes-to-the-very-heart-of-the-doctor-patient-relationship/20005677.article	Dr Amir Hannan	28/01/2014
43	GP source	Pulse Today	Professor Roger Jones: 'I strongly support the care.data initiative'	http://www.pulsetoday.co.uk/news/gp-topics/it/professor-roger-jones-i-strongly-support-the-caredata-initiative/20005693.article	Professor Roger Jones	29/01/2014

44	GP source	Pulse Today	GP survey reveals extent of care.data ignorance among patients and staff	http://www.pulsetoday.co.uk/news/gp-topics/it/gp-survey-reveals-extent-of-caredata-ignorance-among-patients-and-staff/20005713.article	Not stated	31/01/2014
45	GP source	Pulse Today	Analysis: GPs bear brunt of care.data confusion	http://www.pulsetoday.co.uk/views/analysis/analysis-gps-bear-brunt-of-caredata-confusion/20005720.article	Not stated	31/01/2014
46	GP source	Pulse Today	No mandate for care.data grab	Not available to the public	Not stated	31/01/2014
47	Newspaper	The Guardian	We trust no one with our data - not even our doctors: The Snowden revelations could prove bad for our health, thwarting a vital NHS scheme to gather clinical information	https://www.theguardian.com/commentisfree/2014/jan/31/nhs-medical-data-trust-doctors-edward-snowden	Jonathan Freedland	01/02/2014
48	Newspaper	The Guardian	Reply: Letter: What price our medical records?	https://www.theguardian.com/society/2014/feb/02/nhs-medical-records-care-data	Dr Alex May	03/02/2014
49	GP source	Pulse Today	What's so interesting?	http://www.pulsetoday.co.uk/views/blogs/whats-so-interesting/20005728.blog	Dr Hadrian Moss	03/02/2014
50	GP source	Pulse Today	GP hit with contract notice over plan to opt all patients out of care.data	http://www.pulsetoday.co.uk/news/gp-topics/it/gp-hit-with-contract-notice-over-plan-to-opt-all-patients-out-of-caredata/20005749.article	Alex Matthews-King	04/02/2014

51	Newspaper	Daily Mail	HE OPTS HIS ENTIRE PRACTICE OUT OF SCHEME TO HARVEST MEDICAL DATA	Not available to the public	Andy Dolan And Sophie Borland	05/02/2014
52	Newspaper	Mail Online	NHS 'bullies' threaten to axe GP for keeping his patients' records private: He opts his entire practice out of scheme to harvest medical data	https://www.dailymail.co.uk/news/article-2551900/NHS-bullies-threaten-axe-GP-keeping-patients-records-private.html	Andy Dolan And Sophie Borland	05/02/2014
53	Newspaper	The Telegraph	GP fears losing job over pledge to keep patient data private;<br>A GP who pledged to keep his patients' confidential medical information private fears he could be ousted from his practice after NHS bosses said he could be found in breach of contract if he opts his patients out of the scheme	https://www.telegraph.co.uk/news/nhs/10618814/GP-fears-losing-job-over-pledge-to-keep-patient-data-private.html	Claire Carter	05/02/2014
54	Newspaper	Daily Mail	I won't give in to the NHS thought police who want to sell your private medical records	https://www.dailymail.co.uk/debate/article-2552717/I-wont-NHS-Thought-Police-want-sell-private-medical-records-GP-threatened-health-chiefs-hits-back.html	Dr Gordon Gancz	06/02/2014
55	GP source	Pulse Today	Saying no to care.data = saying no to care?	http://www.pulsetoday.co.uk/saying-no-to-caredata-saying-no-to-care/20005801.blog	Dr Tony Copperfield	07/02/2014
56	Newspaper	Daily Mail	INSURERS COULD USE NEW NHS DATABASE TO TRACK YOU DOWN WITHIN 2 HOURS'	https://www.dailymail.co.uk/news/article-2554437/Insurers-use-new-NHS-database-track-two-hours.html	Sophie Borland	08/02/2014
57	Newspaper	The Times	Share your data with the NHS. It's safe and beneficial;<br>Patient information will be secure in a confidential system and will be used only appropriately	https://www.thetimes.co.uk/article/share-your-data-with-the-nhs-its-safe-and-beneficial-cjmzw36s0jp	Dr Clare Gerada	08/02/2014

58	Newspaper	Mail Online	Big Brother' database will grab children's health records but parents are being kept in the dark	https://www.dailymail.co.uk/news/article-2554959/Big-Brother-database-grab-childrens-health-records-parents-kept-dark.html	Martin Beckford	09/02/2014
59	Newspaper	The Telegraph	The brave doctor taking on Big Brother;<br>We owe a debt to the family GP who refuses to give up his patients' medical records to the Health and Social Care Information Centre	https://www.telegraph.co.uk/news/nhs/10627492/The-brave-doctor-taking-on-Big-Brother.html	Max Pemberton	10/02/2014
60	Newspaper	The Telegraph	Crisis of confidence' in NHS database, warn GPs;<br>Stall NHS data-sharing amid 'crisis of confidence' GPs say	https://www.telegraph.co.uk/news/health/news/10634539/Crisis-of-confidence-in-NHS-database-warn-GPs.html	Laura Donnelly	12/02/2014
61	Media	BBC	GPs voice fears over giant patient records database	https://www.bbc.co.uk/news/health-26151458	Nick Triggle	12/02/2014
62	GP source	Pulse Today	RCGP demands new publicity campaign to address 'crisis in confidence' over care.data scheme	http://www.pulsetoday.co.uk/news/gp-topics/it/rcgp-demands-new-publicity-campaign-to-address-crisis-in-confidence-over-caredata-scheme/20005847.article	Alex Matthews-King	12/02/2014
63	Newspaper	Daily Mail	GPS WARN OF CRISIS IN PUBLIC CONFIDENCE OVER NHS DATABASE	https://www.dailymail.co.uk/news/article-2558135/GPs-warn-crisis-public-confidence-NHS-database-Royal-Collage-warns-health-service-failing-inform-patients-data-sharing.html	Sophie Borland	13/02/2014
64	Newspaper	The Times	GPs fear failure of patient data scheme	https://www.thetimes.co.uk/article/gps-fear-failure-of-patient-data-scheme-6csvc9wj3l2	Chris Smyth	13/02/2014

65	GP source	Pulse Today	Two-thirds of public 'don't recall receiving care.data information leaflet'	http://www.pulsetoday.co.uk/news/gp-topics/it/two-thirds-of-public-dont-recall-receiving-caredata-information-leaflet/20005874.article	Alex Matthews-King	14/02/2014
66	Newspaper	Mail Online	Two-thirds oppose plans for new NHS database that will see confidential medical records sold to private firms	https://www.dailymail.co.uk/news/article-2560335/Two-thirds-oppose-plans-new-NHS-database-confidential-medical-records-sold-private-firms.html	Not stated	15/02/2014
67	Newspaper	The Daily Telegraph	NHS admits database could pose privacy risk; NHS database could be disaster, say campaingers	https://www.telegraph.co.uk/news/nhs/10642740/NHS-admits-new-medical-records-database-could-pose-privacy-risk.html	Laura Donnelly	17/02/2014
68	Newspaper	The Independent	Your life in their hands: is the care.data NHS database a healthy step or a gross invasion of patient privacy?;<br>Doctors are worried that centralising our most personal records will break the bond of trust that underpins the NHS	https://www.independent.co.uk/life-style/health-and-families/health-news/your-life-in-their-hands-is-the-caredata-nhs-database-a-healthy-step-or-a-gross-invasion-of-patient-9042564.html	Charlie Cooper	17/02/2014
69	Newspaper	The Independent	Doctors raise fears over sharing NHS patient records;<br>BMA 'deeply concerned' with Government's public information campaign for care.data project	https://www.independent.co.uk/life-style/health-and-families/health-news/doctors-raise-fears-over-sharing-nhs-patient-records-9133807.html	Jane Kirby, Ella Pickover	17/02/2014
70	Newspaper	Mail Online	Now doctors' union has 'grave doubts' over plan to harvest patient data: British Medical Association warns public has been left in the dark over the scheme	https://www.dailymail.co.uk/news/article-2561662/Now-doctors-union-grave-doubts-plan-harvest-patient-data-British-Medical-Association-warns-public-left-dark-scheme.html	Sophie Borland	17/02/2014
71	Newspaper	London Evening Standard	Concern at medical records sharing	Not available to the public	Not stated	17/02/2014

72	GP source	Pulse Today	GPC calls for urgent talks over public awareness of care.data scheme	http://www.pulsetoday.co.uk/news/gp-topics/it/gpc-calls-for-urgent-talks-over-public-awareness-of-caredata-scheme/20005884.article	Alex Matthews-King	17/02/2014
73	GP source	BMA	Call to boost public awareness of data sharing	https://www.bma.org.uk/news/2014/february/call-to-boost-public-awareness-of-data-sharing	Not stated	17/02/2014
74	Newspaper	The Daily Telegraph	Delay NHS database until public is dear, say experts	Not available to the public	Laura Donnelly	18/02/2014
75	Newspaper	Daily Mail	NOW DOCTORS' UNION HAS GRAVE DOUBTS' OVER PLAN TO HARVEST PATIENT DATA	https://www.dailymail.co.uk/news/article-2561662/Now-doctors-union-grave-doubts-plan-harvest-patient-data-British-Medical-Association-warns-public-left-dark-scheme.html	Sophie Borland	18/02/2014
76	Newspaper	The Guardian	NHS in England delays sharing of medical records	https://www.theguardian.com/society/2014/feb/18/nhs-delays-sharing-medical-records-care-data	Peter Walker	18/02/2014
77	Newspaper	The Independent	Victory for privacy as NHS database is delayed;<br>Collection of confidential medical records put on hold after backlash. Critics feared personal information would be vulnerable to hackers	https://www.independent.co.uk/life-style/health-and-families/health-news/victory-for-privacy-as-nhs-database-is-delayed-9137136.html	Charlie Cooper	18/02/2014
78	Newspaper	The Independent	Doctors raise fears over sharing NHS patient medical records;<br>BMA 'deeply concerned' with Government's public information campaign for care.data project	https://www.independent.co.uk/life-style/health-and-families/health-news/doctors-raise-fears-over-sharing-nhs-patient-records-9133807.html	Jane Kirby, Ella Pickover	18/02/2014
79	Newspaper	Mail Online	Controversial plan to share medical records across<br>NHS is put on hold for six months	Not available to public	Sam Webb	18/02/2014

80	Newspaper	London Evening Standard	Medical records share plan delayed	https://www.standard.co.uk/panewsfeeds/medical-records-share-plan-delayed-9136528.html	Not stated	18/02/2014
81	Newspaper	London Evening Standard	Records plan clarification urged	Not available to the public	Not stated	18/02/2014
82	Newspaper	The Telegraph	Patients should be warned before NHS shares medical records, doctors say;<br>Growing pressure on ministers to go back to drawing board on NHS medical records	https://www.telegraph.co.uk/news/health/news/10646151/Patients-should-be-warned-before-NHS-shares-medical-records-doctors-say.html	Laura Donnelly	18/02/2014
83	Newspaper	The Times	Health chiefs delay data-sharing plan in big climbdown	https://www.thetimes.co.uk/article/nhs-chiefs-in-climbdown-over-sharing-patient-data-66396lf825q	Chris Smyth	18/02/2014
84	Web publication	Sky News	NHS Records Database Postponed Amid Concerns	https://news.sky.com/story/nhs-records-database-postponed-amid-concerns-10416834	Not stated	18/02/2014
85	Media	BBC	Giant NHS database rollout delayed	https://www.bbc.co.uk/news/health-26239532	Nick Triggle	18/02/2014
86	GP source	Pulse Today	NHS England delays care.data scheme to 'build understanding' of benefits	http://www.pulsetoday.co.uk/news/gp-topics/it/nhs-england-delays-caredata-scheme-to-build-understanding-of-benefits/20005896.article	Alex Matthews-King	18/02/2014
87	Newspaper	The Daily Telegraph	NHS database put on hold as officials accept privacy fears	Not available to the public	Laura Donnelly	19/02/2014

88	Newspaper	Daily Mail	HOW CONCERNS GREW	Not available to the public	Sophie Borland	19/02/2014
89	Newspaper	The Guardian	NHS health record database delayed after public outcry: New system put on hold for at least six months: Critics say NHS England has failed to put case for it	Not available to the public	Peter Walker, James Meikle and Randeep Ramesh	19/02/2014
90	Newspaper	The Independent	Victory for privacy as NHS database delayed; * Collection of confidential medical records put on hold after backlash * Critics feared personal information would be vulnerable to hackers Data-sharing delay gives NHS 'time to get it right'	https://www.independent.co.uk/life-style/health-and-families/health-news/victory-for-privacy-as-nhs-database-is-delayed-9137136.html	Charlie Cooper	19/02/2014
91	Newspaper	Mail Online	NHS delays plan to harvest your details: Victory for the Mail as database is shelved for six months	https://www.dailymail.co.uk/news/article-2562296/Controversial-plan-share-medical-records-NHS-hold-six-months.html	Sophie Borland	19/02/2014
92	Newspaper	The Telegraph	NHS database 'vital' to improve cancer research; Medical records database 'vital' for improving the country's poor cancer survival record, NHS chief says	https://www.telegraph.co.uk/news/health/news/10649786/NHS-database-vital-to-improve-cancer-research.html	Laura Donnelly	19/02/2014
93	Newspaper	The Times	NHS chiefs in climbdown over sharing patient data	https://www.thetimes.co.uk/article/nhs-chiefs-in-climbdown-over-sharing-patient-data-66396lf825q	Chris Smyth	19/02/2014
94	GP source	Pulse Today	Rebel GP told contract notice over care.data opt-outs was a 'misunderstanding'	http://www.pulsetoday.co.uk/news/gp-topics/it/rebel-gp-told-contract-notice-over-caredata-opt-outs-was-a-misunderstanding/20005908.article	Alex Matthews-King	19/02/2014

95	GP source	BMA	Doctors' concerns prompt data sharing plan delay	https://www.bma.org.uk/news/2014/february/doctors-concerns-prompt-data-sharing-plan-delay	Not stated	19/02/2014
96	Newspaper	The Daily Telegraph	Database a fundamental part of the fight against cancer, says NHS chief;<br>Director defends scheme as crucial to future of the health service	Not available to the public	Laura Donnelly	20/02/2014
97	Newspaper	The Times	NHS must quell fears over data or lives will be lost, says charity	https://www.thetimes.co.uk/article/nhs-must-quell-fears-over-data-or-lives-will-be-lost-says-charity-z5rj2m7qwhc	Michael Savage and Chris Smyth	20/02/2014
98	Media	BBC	Critics of giant NHS database 'are scaremongering'	https://www.bbc.co.uk/news/health-26277866	Nick Triggle and Adam Brimelow	21/02/2014
99	GP source	BMA	GPC Newsletter	https://www.bma.org.uk/connecting-doctors/the_practice/m/mediagallery/24	Not stated	21/02/2014
100	Newspaper	The Daily Telegraph	Data don't help cancer;<br>Letters to the Editor	Not available to public	Dr Neil Bhatia	22/02/2014
101	Newspaper	The Telegraph	NHS medical records database could help prevent disease, senior doctors say;<br>Lord Darzi and Lord Crisp have joined a group of health professionals to defend the NHS medical records database, days after it was put on hold following concerns about data sharing	https://www.telegraph.co.uk/news/health/news/10657580/NHS-medical-records-database-could-help-prevent-disease-senior-doctors-say.html	Claire Carter	24/02/2014
102	Newspaper	The Times	Healthcare data;<br>If data linkage in the UK had not been undertaken in the past we would know less about the causes of disease and be ignorant of inequities in access to care	Not available to the public	Not stated	24/02/2014

103	Newspaper	The Times	Medical advances need sharing of data, say doctors	https://www.thetimes.co.uk/article/medical-advances-need-sharing-of-data-say-doctors-s6flk6l6dn2	Oliver Moody	24/02/2014
104	Media	BBC	NHS data-sharing project at risk, say MPs	https://www.bbc.co.uk/news/health-26347026	Nick Triggle	25/02/2014
105	Media	BBC	NHS data-sharing will have 'enormous benefits', say peers	http://www.bbc.co.uk/democracylive/house-of-lords-26343037	Not stated	25/02/2014
106	GP source	Pulse Today	Debate: Should GPs back care.data?	http://www.pulsetoday.co.uk/news/gp-topics/it/debate-should-gps-back-caredata/20005959.article	Not stated	25/02/2014
107	Newspaper	The Independent	Atos to manage NHS care.data project despite ongoing 'mess' over disability benefit assessments;<br>Despite the Disability Minister Mike Penning admitting the reassessments were in 'chaos' Atos will roll out the controversial care.data project	https://www.independent.co.uk/news/atos-to-work-on-nhs-care-data-project-despite-ongoing-mess-over-disability-benefit-assessments-9153885.html	Felicity Morse	26/02/2014
108	Newspaper	The Times	GPs force rethink on data-sharing plan;	https://www.thetimes.co.uk/article/gps-force-rethink-on-data-sharing-plan-hlkzfj96qzs	Michael Savage	26/02/2014
109	Newspaper	The Times	The pitfalls of the NHS central database plan; Letters to the Editor	https://www.thetimes.co.uk/article/the-pitfalls-of-the-nhs-central-database-plan-qts55x7pdjq	Not stated	26/02/2014
110	Newspaper	The Times	Opt-out easier in GP data-sharing rethink	Not available to the public	Michael Savage	26/02/2014

111	GP source	Pulse Today	Three in four GPs believe care.data should be 'opt in'	http://www.pulsetoday.co.uk/news/gp-topics/it/three-in-four-gps-believe-caredata-should-be-opt-in/20005954.article	Alex Matthews-King	26/02/2014
112	GP source	BMA	Patient trust threatened by data-sharing concerns	https://www.bma.org.uk/news/2014/february/patient-trust-threatened-by-data-sharing-concerns	Not stated	26/02/2014
113	Newspaper	The Daily Telegraph	GPs back 'opt-in' NHS database	Not available to the public	Not stated	27/02/2014
114	Newspaper	The Independent	Atos to work on NHS care data project despite ongoing 'mess' over disability benefit assessments;<br>Despite the Disability Minister Mike Penning admitting the reassessments were in 'chaos' Atos will roll out the controversial care.data project	https://www.independent.co.uk/news/atos-to-work-on-nhs-care-data-project-despite-ongoing-mess-over-disability-benefit-assessments-9153885.html	Felicity Morse	27/02/2014
115	Newspaper	Mail Online	Cashing in on patient records to be banned: But you'll still have to opt out to keep private details off database	https://www.dailymail.co.uk/news/article-2570567/Cashing-patient-records-banned-But-youll-opt-private-details-database.html	James Chapman and Andy Dolan	28/02/2014
116	GP source	Pulse Today	Update: Care.data put on hold	http://www.pulsetoday.co.uk/views/analysis/update-caredata-put-on-hold/20005997.article	Nigel Praities	28/02/2014
117	Newspaper	The Daily Telegraph	NHS data for sale: will your premiums rise?; Insurance Nicole Blackmore examines the Government's plans for a new database and how it could be exploited by insurers	Not available to the public	Nicole Blackmore	01/03/2014
118	Newspaper	Daily Mail	CASHING IN ON PATIENT RECORDS TO BE BANNED	https://www.dailymail.co.uk/news/article-2570567/Cashing-patient-records-banned-But-youll-opt-private-details-database.html	James Chapman And Andy Dolan	01/03/2014

119	Newspaper	London Evening Standard	NHS data will not be sold: Hunt	https://www.standard.co.uk/panewsfeeds/nhs-data-will-not-be-sold-hunt-9161694.html	Not stated	01/03/2014
120	Newspaper	The Times	NHS patient data to be made confidential	https://www.thetimes.co.uk/article/nhs-patient-data-to-be-made-confidential-gdxmctdnh56	Tom Whipple	01/03/2014
121	GP source	Pulse Today	Legislation will prevent patient data being sold outside the health service, says Hunt	http://www.pulsetoday.co.uk/news/gp-topics/it/legislation-will-prevent-patient-data-being-sold-outside-the-health-service-says-hunt/20006014.article	Alex Matthews-King	03/03/2014
122	Newspaper	The Daily Telegraph	Hospital records used by private marketing firms;<br>NHS data helps firms 'tailor media campaigns'	https://www.telegraph.co.uk/news/nhs/10674639/NHS-hospital-records-used-by-private-marketing-firms.html	Laura Donnelly	04/03/2014
123	Newspaper	Mail Online	BY THE WAY . . . GP RECORDS DON'T TELL THE WHOLE STORY	Not available to the public	Dr Martin Scurr	04/03/2014
124	Newspaper	The Guardian	Reply: Letter: Taking better care of health data	https://www.theguardian.com/society/2014/mar/04/taking-better-care-health-data	Various<br>Prof Harvey Goldstein University of Bristol, Prof Ruth Gilbert University College London, Dr Katie Harron UCL, Dr Gareth Hagger-Johnson UCL, Dr Mario Cortina UCL ,	05/03/2014

					Dr Nirupa Dattani City University	
125	Newspaper	The Guardian	Reply: Letter: Taking better care of health data	https://www.theguardian.com/society/2014/mar/04/taking-better-care-health-data	Professor Robert Boyd	05/03/2014
126	Newspaper	The Telegraph	NHS database: will it push up your insurance premiums?;<br>Experts fear insurers will buy medical information about customers and use it to price policies	https://www.telegraph.co.uk/finance/personalfinance/insurance/10667245/NHS-database-will-it-push-up-your-insurance-premiums.html	Nicole Blackmore	05/03/2014
127	GP source	Pulse Today	Falling off the care.data fence	http://www.pulsetoday.co.uk/views/blogs/falling-off-the-caredata-fence/20006012.blog	Not stated	05/03/2014
128	GP source	BMA	GPC Newsletter	Not available to the public<br><br>file:///Users/yaldakazempour/Downloads/11%20GPC%20Newsletter%2006%2003%2014%20(3).pdf	Not stated	06/03/2014
129	Newspaper	The Times	Health data centre pledges more honesty	https://www.thetimes.co.uk/article/health-data-centre-pledges-more-honesty-m7qpfbtx8qf	Chris Smyth	07/03/2014
130	GP source	BMA	BMA remains concerned over special measures safeguards	https://www.bma.org.uk/news/2014/march/bma-remains-concerned-over-special-measures-safeguards	Not stated	07/03/2014
131	Media	BBC	Government outlines data-sharing safeguards	http://www.bbc.co.uk/democracylive/house-of-commons-26518046	Not stated	10/03/2014

132	Media	BBC	MPs agree to data-sharing safeguards	http://www.bbc.co.uk/democracylive/house-of-commons-26532173	Not stated	11/03/2014
133	Newspaper	The Times	Sharing medical data would turn UK into 'best clinical laboratory in world'	https://www.thetimes.co.uk/article/sharing-medical-data-would-turn-uk-into-best-clinical-laboratory-in-world-sg639w8m5cj	Chris Smyth	21/03/2014
134	GP source	Pulse Today	Patients would 'embrace' care.data if they understood it, jet lag cure, crackdown on slum landlords and exploding cells to fight cancer	http://www.pulsetoday.co.uk/patients-would-embrace-caredata-if-they-understood-it-jet-lag-cure-crackdown-on-slum-landlords-and-exploding-cells-to-fight-cancer/20006171.article	Christian Duffin	21/03/2014
135	GP source	BMA	Care.data confidentiality concerns cannot be ignored, say doctors	https://www.bma.org.uk/news/2014/march/caredata-confidentiality-concerns-cannot-be-ignored-say-doctors	Dr Lucy-Jane Davis	21/03/2014
136	GP source	BMA	GPC Newsletter	Not available to the public	Not stated	21/03/2014
137	Newspaper	The Independent	Medical chief defends NHS data-sharing; HEALTH	Not available to the public	Paul Bignell	22/03/2014
138	Newspaper	Daily Mail	MORE DELAYS FOR NHS DATA HARVESTING PROGRAMME	https://www.dailymail.co.uk/news/article-2608134/More-delays-controversial-NHS-data-harvesting-programme-Government-adviser-says-scheme-mishandled.html	Not stated	19/04/2014

139	GP source	BMA	GPC Newsletter	https://www.bma.org.uk/connecting-doctors/the_practice/m/mediagallery/48		22/04/2014
140	Newspaper	The Daily Telegraph	Patient data in peril;<br>Letters to the Editor	Not available to the public	Professor John R Ashton	07/05/2014
141	GP source	Pulse Today	Practice opts all patients out of care.data despite delay to roll-out	http://www.pulsetoday.co.uk/news/gp-topics/it/practice-opts-all-patients-out-of-caredata-despite-delay-to-roll-out/20006682.article	Not stated	12/05/2014
142	Newspaper	The Telegraph	Britons 'trust banks more than government' to protect their data;<br>The government has much work to do reassuring the public of its data security measures, according to Unisys	https://www.telegraph.co.uk/technology/internet-security/10846656/Britons-trust-banks-more-than-government-to-protect-their-data.html	Sophie Curtis	21/05/2014
143	GP source	Pulse Today	GPs vote in favour of an opt-in system for care.data	http://www.pulsetoday.co.uk/news/gp-topics/it/gps-vote-in-favour-of-an-opt-in-system-for-caredata/20006796.article	Alex Matthews-King	23/05/2014
144	GP source	BMA	Patient data extraction requires explicit consent, say GPs	https://www.bma.org.uk/news/2014/may/patient-data-extraction-requires-explicit-consent-say-gps	Not stated	27/05/2014
145	Newspaper	The Times	GPs force rethink on data-sharing plan	https://www.thetimes.co.uk/article/gps-force-rethink-on-data-sharing-plan-hlkzfj96qzs	Michael Savage	17/06/2014
146	Newspaper	Daily Mail	AND OFFICIALS DON'T EVEN KNOW WHERE DETAILS OF 1.3M PATIENTS ENDED UP	Not available to the public	Sophie Borland	18/06/2014

147	GP source	Pulse Today	Parts of care.data should be 'opt-in' only, says NHS England director	http://www.pulsetoday.co.uk/news/gp-topics/it/parts-of-caredata-should-be-opt-in-only-says-nhs-england-director/20007039.article	Sofia Lind	20/06/2014
148	Newspaper	Mail Online	CONSENT CONCERNS ON MEDICAL RECORDS	Not available to the public	Not stated	25/06/2014
149	GP source	Pulse Today	Over half the public still unaware of care.data	http://www.pulsetoday.co.uk/news/gp-topics/it/over-half-the-public-still-unaware-of-caredata/20007087.article	Alex Matthews-King	25/06/2014
150	GP source	BMA	Patients' medical data sacrosanct, declares BMA	https://www.bma.org.uk/news/2014/june/patients-medical-data-sacrosanct-declares--bma	Not stated	25/06/2014
151	GP source	Pulse Today	GPs to send patient letters explaining care.data 'opt-out'	http://www.pulsetoday.co.uk/news/gp-topics/it/gps-to-send-patient-letters-explaining-caredata-opt-out/20007191.article	Alex Matthews-King	03/07/2014
152	GP source	BMA	Call to involve patients in how data is shared	https://www.bma.org.uk/news/2014/july/call-to-involve-patients-in-how-data-is-shared	Not stated	03/07/2014
153	GP source	Pulse Today	Make care.data opt in or we will opt patients out, say GPs	http://www.pulsetoday.co.uk/news/gp-topics/it/make-caredata-opt-in-or-we-will-opt-patients-out-say-gps/20007472.article	Alex Matthews-King	05/08/2014
154	GP source	Pulse Today	GPs' care.data responsibilities amount to 'good customer service', says information commissioner	http://www.pulsetoday.co.uk/news/gp-topics/it/gps-caredata-responsibilities-amount-to-good-customer-service-says-information-commissioner/20007450.article	Alex Matthews-King	05/08/2014

155	GP source	Pulse Today	Police should have greater access to medical records, says chief constable	http://www.pulsetoday.co.uk/news/gp-topics/it/police-should-have-greater-access-to-medical-records-says-chief-constable/20007529.article	Alex Matthews-King	11/08/2014
156	GP source	Pulse Today	NHS England looks at expanding care.data extractions to include 'sensitive' patient information	http://www.pulsetoday.co.uk/news/gp-topics/it/nhs-england-looks-at-expanding-caredata-extractions-to-include-sensitive-patient-information/20007611.article	Alex Matthews-King	19/08/2014
157	GP source	Pulse Today	Selling patient data poses risks to GPs and patients alike	http://www.pulsetoday.co.uk/views/blogs/inside-information/selling-patient-data-poses-risks-to-gps-and-patients-alike/20007916.blog	Dr Hadrian Moss	17/09/2014
158	GP source	Pulse Today	GPs could opt all patients out of care.data opt under data protection laws, information office says	http://www.pulsetoday.co.uk/news/gp-topics/it/gps-could-opt-all-patients-out-of-caredata-opt-under-data-protection-laws-information-office-says/20008114.article	Alex Matthews-King	06/10/2014
159	Newspaper	Mail Online	Storm as NHS gives go-ahead to patient database despite concerns: Pilot scheme will involve 1.7million people unless an individual specifically ops out	https://www.dailymail.co.uk/news/article-2784269/Storm-NHS-gives-ahead-patient-database-despite-concerns-Pilot-scheme-involve-1-7million-people-unless-individual-specifically-ops-out.html	Sophie Borland	07/10/2014
160	GP source	Pulse Today	GPs to contact patients individually to explain care.data opt-out	http://www.pulsetoday.co.uk/news/gp-topics/it/gps-to-contact-patients-individually-to-explain-caredata-opt-out/20008134.article	Alex Matthews-King	07/10/2014

161	Newspaper	Mail Online	NHS data is snooped on six times every day: Staff caught looking at records of friends, family and even love rivals	https://www.dailymail.co.uk/news/article-2833960/NHS-data-snooped-six-times-day-Staff-caught-looking-records-friends-family-love-rivals.html?ITO=1490&ns_mchannel=rss&ns_campaign=1490	Sophie Borland	14/11/2014
162	GP source	Pulse Today	Care.data extractions on hold until NHS England satisfies GP concerns	http://www.pulsetoday.co.uk/news/gp-topics/it/caredata-extractions-on-hold-until-nhs-england-satisfies-gp-concerns/20008789.article	Alex Matthews-King	18/12/2014

peer review only

BMJ Open

Media content analysis of general practitioners' reactions to care.data expressed in the media: what lessons can be learned for future NHS data sharing initiatives?

Journal:	*BMJ Open*
Manuscript ID	bmjopen-2020-038006.R1
Article Type:	Original research
Date Submitted by the Author:	10-Jul-2020

Complete List of Authors:	Ford, Elizabeth; Brighton & Sussex Medical School,<br>Kazempour, Yalda; Brighton & Sussex Medical School, Primary Care and Public Health<br>Cooper, Maxwell; Brighton & Sussex Medical School<br>Katikireddi, Srinivasa; University of Glasgow School of Life Sciences, MRC/CSO Social & Public Health Sciences Unit<br>Boyd, Andy; University of Bristol, School of Social and Community Medicine

**Primary Subject Heading**:	General practice / Family practice
Secondary Subject Heading:	Communication, Epidemiology, Ethics, Health informatics, Health policy
Keywords:	Health informatics < BIOTECHNOLOGY & BIOINFORMATICS, EPIDEMIOLOGY, MEDICAL ETHICS, PRIMARY CARE, PUBLIC HEALTH

SCHOLARONE™
Manuscripts

I, the Submitting Author has the right to grant and does grant on behalf of all authors of the Work (as defined in the below author licence), an exclusive licence and/or a non-exclusive licence for contributions from authors who are: i) UK Crown employees; ii) where BMJ has agreed a CC-BY licence shall apply, and/or iii) in accordance with the terms applicable for US Federal Government officers or employees acting as part of their official duties; on a worldwide, perpetual, irrevocable, royalty-free basis to BMJ Publishing Group Ltd ("BMJ") its licensees and where the relevant Journal is co-owned by BMJ to the co-owners of the Journal, to publish the Work in this journal and any other BMJ products and to exploit all rights, as set out in our licence.

The Submitting Author accepts and understands that any supply made under these terms is made by BMJ to the Submitting Author unless you are acting as an employee on behalf of your employer or a postgraduate student of an affiliated institution which is paying any applicable article publishing charge ("APC") for Open Access articles. Where the Submitting Author wishes to make the Work available on an Open Access basis (and intends to pay the relevant APC), the terms of reuse of such Open Access shall be governed by a Creative Commons licence – details of these licences and which Creative Commons licence will apply to this Work are set out in our licence referred to above.

Other than as permitted in any relevant BMJ Author's Self Archiving Policies, I confirm this Work has not been accepted for publication elsewhere, is not being considered for publication elsewhere and does not duplicate material already published. I confirm all authors consent to publication of this Work and authorise the granting of this licence.

For peer review only

Media content analysis of general practitioners' reactions to care.data expressed in the media: what lessons can be learned for future NHS data sharing initiatives?

Elizabeth Ford[1]*, Yalda Kazempour[1], Max Cooper[1], Srinivasa Vittal Katikireddi[2], Andy Boyd[3]

1: Department of Primary Care and Public Health, Brighton and Sussex Medical School, Brighton UK.

2: MRC/CSO Social & Public Health Sciences Unit, University of Glasgow

3: Population Health Sciences, Bristol Medical School, University of Bristol, Bristol, UK.

*Corresponding Author:

Dr Elizabeth Ford
Senior Lecturer in Primary Care Research
Department of Primary Care and Public Health
Brighton and Sussex Medical School
Room 322 Watson Building
Village Way,
Falmer
BRIGHTON
BN1 9PH
+44 (0) 1273 641974
e.m.ford@bsms.ac.uk

For peer review only - http://bmjopen.bmj.com/site/about/guidelines.xhtml

Abstract

Objectives: Care.data was a 2013 UK government initiative to extract patient data from general practices in England to form a centralised whole-population database for service planning and health research. After a public outcry, the scheme was postponed and cancelled. Public views of care.data have previously been analysed; this study aimed to understand contemporary general practitioners' (GPs) views of the scheme, which may have been influential in its downfall.

Design: Systematic search of media articles, followed by media content analysis.

Setting: UK-based mainstream and GP-facing media in 2013 and 2014.

Participants: Articles were eligible if they focused on care.data, and GPs were quoted, authored the article, or if articles were written for a majority GP audience.

Interventions: N/A

Primary and Secondary outcome measures: Themes which explained GPs reactions to care.data and which could explain support for or opposition to the scheme.

Results: 162 media articles met inclusion criteria and were drawn from newspapers, news websites and GP facing websites. GPs recognised care.data's potential value for research and improving care, but had grave concerns about the scheme's implementation. These centred the lack of safeguards and purpose around the scheme which meant patients were not able to make informed decisions about opt-out. GPs perceived they were poorly resourced to meet competing demands to both share patients' data and protect confidentiality. They distrusted the government's likely uses of the data and perceived a risk of patient re-identification if the data were sold onto commercial entities.

Conclusions: Findings show specific concerns which GPs had about care.data which led to the withdrawal of support. Future NHS patient data-sharing schemes should engage with GPs and other clinicians as key stakeholders from the earliest moments of planning, so that their views and needs are incorporated into the design of such schemes.

Strengths and Limitations of this Study:

- We systematically and comprehensively searched mainstream and GP facing media published during the unfolding care.data events, to access the voice of doctors as events unfolded, thus minimising recall bias in accounts of events.

For peer review only

- By following a systematic approach, we minimised bias in the selection of articles for the study. However, other sources of GP opinions (such as social media discussions) escaped our analysis.

- Doctors contributing to mainstream news articles may not be representative of GPs in general, but instead can be viewed as key contributors to the prominent circulating discourses at the time.

- GPs views expressed at the time of the debate may not represent their current views on the care.data scheme, its subsequent failure, or on future schemes with similar aims.

Funding Statement:

SVK acknowledges funding from a NRS Senior Clinical Fellowship (SCAF/15/02), the Medical Research Council (MRC) (MC_UU_12017/13 & MC_UU_12017/15) and the Scottish Government Chief Scientist Office (SPHSU13 & SPHSU15).

AB acknowledges funding from the Avon Longitudinal Study of Parents and Children: MRC and Wellcome (Grant ref: 102215/2/13/2) and the CLOSER longitudinal research consortium: Economic and Social Research Council (ES/K000357/).

Competing Interest:

All authors declare no competing interests.

For peer review only - http://bmjopen.bmj.com/site/about/guidelines.xhtml

Introduction

Care.data

In 2012, laws were passed within the United Kingdom Health and Social Care Act (HSCA) to require all general practitioners (GPs; family doctors) in England to upload their electronic patient records (EPRs) to a central database which could then be linked with additional NHS datasets and used for research, audit, and planning health services. This programme of data collation and re-use was called care.data and was due to be initiated in Spring 2014 [1]. It expanded upon previous data collections which were limited to hospital records or were opt-in at the GP practice level [2-5]. For care.data and previous schemes, provisions were made so that individual patients who did not wish their data to be used for quality improvement and research purposes could opt-out [1].

The care.data scheme was advertised to patients in the form of a leaflet "*Better information means better care*" [6] which was purportedly delivered to all households in England by Royal Mail in January 2014, although few people reported receiving it [7]. There was no opt-out form on the leaflet and no clear instructions on how to register an opt-out from the scheme [8]. A survey of 1400 members of public showed that only 23% remembered receiving the leaflets and 45% did not understand the care.data scheme [9]. Furthermore, a survey of 600 GPs showed that 80% of them also felt that they did not have adequate knowledge of the scheme [9].

In early 2014 the programme faced critical reception from patients and other stakeholders, which was widely reported in the media. This adverse media coverage, and the rise of campaign groups [e.g. 10], prompted a series of damaging disclosures by the NHS relating to past sharing of centralised health records for purposes beyond those relating to improving the public good [11]. The high media profile and widespread criticism of care.data led to a review of the scheme (the Partridge review [12]), and the UK government postponed the care.data programme in February 2014, finally cancelling it in 2016 [13]. A full timeline is shown in Figure 1.

Figure Legend: Figure 1: Summary of key events regarding unfolding of care.data throughout 2013-2014 (based on Presser et al., 2015 [11]).

<Figure 1 about here>

Following a lengthy consultation [14], a new national strategy for an opt-out model for re-use of NHS patient data was rolled out in May 2018 as the "National Data Opt-Out" [15]. At the current time, future universal data sharing schemes for primary care records under this new strategy are not clear. Given the high likelihood of similar schemes being launched in the near future, it is imperative that

For peer review only - http://bmjopen.bmj.com/site/about/guidelines.xhtml

as many lessons as possible are learned from care.data to promote transparency of such schemes and inclusivity of all stakeholders [16].

Stakeholder reviews on why care.data went wrong have focused on patient and public views

A body of literature has built up which has looked at themes in the public's objections to care.data and why it became so unpopular. These studies have included analyses of citizens' comments on the care.data website and the Guardian newspaper comments section [17], and public views posted on Twitter [8, 18]. Commentators have drawn lessons from public reactions to the care.data scheme to suggest recommendations for future schemes [11], to suggest a theoretical framework for explaining public concern about the scheme [19], and to guide interpretation of new data protection legislation [20].

Why focus on General Practitioners?

Any patient data-sharing scheme requires the support of clinicians as central stakeholders in the project, and as the creators and custodians of patient data. In the United Kingdom, GPs tend to be the legal *owners* of the medical information recorded in their electronic patient records, and in turn are responsible for the way in which these data are processed and used. These responsibilities are enshrined in UK Data Protection Act (the Data Protection Act 1998 at the time of the care.data programme) and the common law duty of confidentiality, and are regulated by the Information Commissioner's Office (ICO).

While the British Medical Association (BMA) was initially in favour of the care.data scheme [21], doctors later wrote publicly about their concerns about the scheme, both before [22] and after it was postponed [23, 24]. It is not clear if opposition to the scheme from GPs was one of the main drivers in the postponement and final failure of the scheme, or if GPs' reservations followed the patient, public and media outcry. To understand more about GPs' views on care.data, and what factors underpinned their later opposition to the scheme, we conducted a media content analysis of contemporaneous GP reactions to the scheme expressed in the media. The aims of this study were to systematically and comprehensively identify, and qualitatively analyse, newspaper and news website coverage of the care.data debate within the UK, focussing specifically on articles which quoted GPs or other doctors, or were authored by GPs or other doctors, or were written for a solely GP audience. We aimed to describe themes and learning points in doctors' views of the scheme and to look for turning points in opinion during the unfolding events.

For peer review only - http://bmjopen.bmj.com/site/about/guidelines.xhtml

Methods

Selection of sources

A systematic search strategy, following principles outlined in PRISMA reporting [25], was used to identify online media about care.data, which quoted, was written by, or was directed towards UK GPs and other medical doctors during the care.data era. Comments posted by readers in response to articles were not used for analysis.

Mainstream newspapers were searched using the database Nexis. This is a comprehensive newspaper database, updated daily, providing full text access to all UK national newspapers, plus regional newspapers, international news providers and a number of trade journals and magazines. Newspapers were included if they had a circulation in print of 100,000 per day or more. The BBC news website was also searched. Through consultation with GPs (n=4) we identified and searched four GP-facing news websites: Pulse Today, GP online, Royal College of General Practitioner's (RCGP) website and BMA website. The search was constrained to 01/01/2013 to 31/12/2014.

The search terms used in the Nexis database were: "care.data" AND ("doctor" OR "dr" OR "general practitioner" OR "GP" OR "clinician" OR "physician" OR "specialist" OR "professor").

The search term used in BBC news and on the GP facing news sites was: care.data.

Inclusion and Exclusion Criteria

All articles were screened in full by author YK to identify if they met eligibility criteria (Table 1). For any ambiguity in whether articles met inclusion criteria, authors EF and YK worked together to reach a consensus on inclusion. See Supplementary File 1 for full screening results.

Table 1: Inclusion and Exclusion Criteria for Articles

Inclusion Criteria	Exclusion Criteria
Article focussed mainly (>50%) on the care.data project	Less than 50% of the content related to care.data;
AND article quoted a GP or doctor expressing an opinion of care.data;	OR they did not include any quotes or discussion about GPs' or doctors' views;
OR was authored by a GP or any doctor	OR the article was a short lead-in to a main story elsewhere in the same edition (which also appears in the sample);
OR was written for a majority GP audience	OR it was a summary of, or link to, formal documentation and surveys;
OR discussed the opinions, attitudes or views of doctors about care.data	OR it was not a news article in its own right.

For peer review only - http://bmjopen.bmj.com/site/about/guidelines.xhtml

Data Analysis

We approached this analysis with a realist ontology and an objectivist epistemology. A thematic analysis was conducted according to the principles of Braun and Clarke [26] in an iterative process.

The content of all articles was imported to NVivo v12 software. NVivo v12 software facilitated reading articles and coding themes as they emerged. A thematic analysis was conducted according to the principles of Braun and Clarke [26] in an iterative process. The first step was for author YK to familiarise herself with the data collected by reading the articles multiple times and noting down initial ideas. Next step was to determine key themes and code features of the data that were relevant to the research questions. Every article obtained from our search was read and coded line by line to generate themes. For every quote authored by GP it was examined to see if it commented to do with our study and it was coded. Two authors (YK and EF) discussed initial themes, and consolidated emerging and disparate themes into a smaller number of nested themes. The articles were then read again and all quotes linked to a final list of themes. The articles related to each code were then grouped together under the same theme. Given the large volume of data, themes are represented using indicative quotes that were the most illustrative for that theme, with attention paid to discordant data.

Results

Search Results

The search returned 923 articles from the Nexis newspaper database (newspapers and web-based publications), BMA website, Pulse Today and BBC, and after screening against eligibility criteria, 162 were found to meet the inclusion criteria. No relevant articles were returned from the search of 'GPonline' or from the RCGP website. The spread of articles is shown in Table 2.

Table 2: Search Results

Database	No. articles returned by search	No. articles included
Nexis newspaper print articles	451	83
Nexis web-based articles	164	2
BMA	48	16
BBC news	24	8
Pulse Today	236	53
Total	923	162

For peer review only - http://bmjopen.bmj.com/site/about/guidelines.xhtml

Article Characteristics

Characteristics of the articles included for the analysis are summarised in Table 3 and full information given in Supplementary File 2.

Table 3 Sources of the articles

Source	N Articles	N different authors	N doctors quotes
The Guardian (including the Observer)	8	8	16
The Times	14	6	13
The Telegraph (including Daily Telegraph)	22	8	29
Daily Mail (including Mail Online)	22	8	22
The Independent	12	5	14
London Evening Standard	5	Unknown	9
Sky news	2	Unknown	2
Pulse Today	53	11	73
BMA	16	Unknown	12
BBC	8	2	8

Thematic Analysis

Thematic analysis revealed five themes regarding GPs' views on care.data. These were:

1. Support for the scheme: care.data would give value for research and may improve care.
2. Concerns about patients lacking informed choice through proposed opt out system.
3. Concerns about legal responsibilities regarding patient data.
4. Concerns about key safeguards – GPs were unable to reassure patients that they would not be re-identified and their data would not be sold onwards.
5. Concerns about trust between patients and doctors.

Quotes presented below can be cross-referenced with Supplementary File 2.

1. Support for the scheme: Care.data would give value for research and may improve care.

Early in the timeline, many GPs were positive about the potential benefits of care.data. The scheme was supported by the RCGP with Dr Clare Gerada, former president of the College, expressing support and committing to work with the BMA and NHS England on the care.data project (5, BMA, May 2013). Dr David Davies, a GP and the Health and Social Care Information Centre's (HSCIC) executive medical director, believed use of linked data would help improve services and allow

For peer review only - http://bmjopen.bmj.com/site/about/guidelines.xhtml

targeted interventions, by determining patients at higher risks of a particular disease (1, Pulse Today, Mar 2013). Dr Davies explained:

‘We’ll be linking data, doing risk profiles on the data then feeding it back to GPs to adapt their care. That’ll be OK because we’re feeding data back to the people who gave it to us for it to be used in direct patient care’ (1, Pulse Today, Mar 2013).

Professor Liam Smeeth, a practising GP and professor in London highlighted the necessity of using patients’ data to run the NHS :

‘These are data that are absolutely necessary to keep the NHS up with the times, to develop new inventions, or to be safe and effective, so we know what’s going on in the health service. …There are people worried about confidentiality but I am hoping there aren’t people sitting around saying we don’t need to do this because we don’t need this data’ (32, Pulse Today, Jan 2014).

Dr Francesca Lasman, a GP in Cambridgeshire believed the care.data scheme would lead to comprehensive and meaningful analysis leading to insights on managing comorbidities in general practice. She said:

‘I think it is vital to develop an understanding of how best to manage complex co-morbidities which exist in general practice. Drug companies and studies focus on one problem but real data from people with multiple conditions and on many medications gives at least a chance of some meaningful analysis and a start for the best approach to tackling prevention and treatments’ (39, Pulse Today, Jan 2014).

A number of medical charities expressed support for the care.data scheme in line with NHS England’s public awareness campaign. Doctors working for these charities warned the public that if too many people opted out of the scheme, it would impact on science which could lead to increased numbers of deaths in the population (32, Pulse Today, Jan 2014). Professor Peter Weissberg of the British Heart Foundation explained how lack of data could lead to delayed discoveries and more deaths:

‘We can carry on making [discoveries] without [it], but we will make them extraordinarily slowly and people will die in the interim. Not only will they die as a consequence of habits and behaviours they have, which we haven’t understood properly, [but] they will die as a consequence of well-meaning attempts by doctors… [who are] treating patients on a daily basis on what we call the evidence base’ (32, Pulse Today, Jan 2014).

For peer review only - http://bmjopen.bmj.com/site/about/guidelines.xhtml

This generalised support for the scheme was not without qualification, however. The BMA greatly emphasised the importance of gaining public trust and confidence in the scheme. Dr Chaand Nagpaul, the chair of the BMA said:

> *'The BMA continues to support the principle of using anonymised data to plan and improve the quality of NHS care for patients. However, this must be done with the support and consent of the public, therefore patients must be made aware of what is happening to their personal information, what the proposals mean and what their rights are if they do not wish their data to be extracted' (72, Pulse Today, Jan 2014).*

2. Concerns about patients lacking informed choice through the proposed opt out system

Doctors were concerned about the care.data opt-out mechanism, and that patients were not well informed enough to make a choice. In August 2013, GPs were given the responsibility of informing their patients of their medical data extraction for care.data during the 8 weeks before the extraction started. However, GPs were not provided extra funding to carry out this task, leading to frustration (154, Pulse Today, Aug 2014). Dr Grant Ingrams, a practising GP in Warwickshire and deputy chair of the General Practitioners Committee's (GPC's) IT subcommittee, told Pulse Today:

> *'…I don't think any GP is going to pay hundreds or thousands out of their own pocket to run a campaign' (154, Pulse Today, Aug 2014).*

A survey of 427 GPs carried out by Pulse Today and reported on 26th February 2014, revealed that only 19% agreed with the opt-out system with 75% supporting a change to an opt-in system. Ninety percent of GPs stated that if an opt-out system were used, NHS England should provide an opt-out form with the "Better Information Means Better Care" leaflets. The GPs expressed concerns that having to opt out could be challenging for particular groups within society, for example migrants (111, Pulse Today, Feb 2014).

RCGP honorary secretary, Professor Nigel Mathers was outspoken about the public's lack of awareness of the scheme and of their right to opt out. He criticised the NHS awareness campaign by stating:

> *"Crucially, where a scheme is based on an opt-out approach, such as in the case of care.data, we believe that it is vital that the NHS is able to show that it is beyond reproach in having done everything practically possible to ensure that patients and the public know about their right to opt out prior to it going ahead" (82, The Telegraph, Feb 2014).*

For peer review only - http://bmjopen.bmj.com/site/about/guidelines.xhtml

Prof Mathers also criticised the leaflet scheme, and announced that since it had not worked, all other possible means of communication such as radio, national TV and online adverts should be used. He recommended that personalised letters should be sent to all households in England. Prof Mathers said:

> *'At present, we are concerned that levels of awareness concerning care.data are very low, and believe that there is a strong case for substantial additional activity over and above that already in place to tackle this' (79, Mail Online, Feb 2014).*

Following the postponement of the scheme, GPs were surveyed on their opinions. Some GPs believed patients' data should only be extracted with their explicit consent and an opt-in system was the only right way it could be done (143, Pulse Today, May 2014). Some GPs went further and decided to opt out all their patients unless they specifically requested to opt in (141, Pulse Today, May 2014). An Oxfordshire GP who had been practising medicine for 40 years had decided to opt out his entire practice due to lack of information about the scheme. Dr Gancz felt *"…people are being bulldozed into giving consent by default"* (53, The Telegraph, Feb 2014).

A number of GPs who had been in favour of the care.data project initially, later stated that they did not approve of the way the government blamed the lack of public support on GPs. A GP from West Sussex, Dr Jeremy Luke, said:

> *'… [The government] has, as usual, tried to blame GPs for their own failure to engage with and listen to the views of patients' (153, Pulse Today, Aug 2014).*

In contrast, some GPs supported an opt-out system and believed an opt-in system would be a retrograde step in research. Dorset GP L-J Evans explained that an opt-in system would rely on every patient consenting for their medical data to be used, which did not work in her opinion. She explained that a disadvantage of the opt-in system would be skewed results and less robust research (150, BMA, Jun 2014).

3. Concerns about legal responsibilities regarding patient data

GPs were required to share their patients' data for care.data by the 2012 HSCA which put them in a legally challenging situation, as this sharing might have breached the common law duty of confidentiality. Doctors' duty of confidence regarding the information they hold about a patient can only be lifted where the patient has an expectation of a secondary usage [27], in this case, to be shared to a centralised NHS data warehouse. GPs perceived that, legally, they could therefore only share data if they were sure that patients were aware of, and fully understood, the care.data

For peer review only - http://bmjopen.bmj.com/site/about/guidelines.xhtml

scheme and their rights to opt out (111, Pulse Today, Feb 2014). One response to this concern was that some GPs opted out their entire registered list of patients, even though this led them to be in breach of contract with the 2012 HSCA. A number of doctors who opted out their entire practice believed that in allowing patients' data to be extracted without patients being fully informed, the doctors would not be doing their duty to protect patients' data and would be in breach of the Data Protection Act (19, Pulse Today, Nov 2013).

The GPC deputy chair warned GPs that opting out entire practices would be 'breaking the law', as GPs had a 'statutory duty' according to the Health and Social Care Act to share their patients' data (21, Pulse Today, Nov 2013). However, GPs believed that if patients complained about not being aware of their data being shared, GPs would be held accountable (as they were data controllers) and not the government (33, Pulse Today, Jan 2014). Dr Grant Ingrams, who was a former chair of the GPC's IT subcommittee and a GP in Warwickshire, explained how he thought GPs were 'confounded' between these conflicting Acts :

> *'…Practices have a lawful obligation under the Health and Social Care Act to send the data to the HSCIC. But an obligation under the Data Protection Act to protect patients' data. It's leaving practices confounded between two rights. If practices aren't sued by one, they'll be sued by another' (6, Pulse Today, Aug 2013).*

Later on, a survey indicated that 8% of GPs would opt out all their patients, despite being aware they would face a legal challenge and the possibility of their contract being terminated (111, Pulse Today, Feb 2014).

4. Concerns about key safeguards – GPs were unable to reassure patients that they would not be re-identified and their data would not be sold onwards.

From the launch of care.data, there had been issues about a lack of transparency and lack of assurance about the confidentiality of data held by HSCIC (139, BMA, Apr 2014; 150, BMA, Jun 2014). One of doctors' main concerns was the possibility of patients being re-identified from their pseudonymised records, this was expressed before the project was even launched (9, Pulse Today, Sep 2013). Data extracted would contain information about the patients' prescriptions, investigation results and some diagnostic codes. However, more sensitive codes such as IVF, abuse, HIV, termination of pregnancy, convictions or STIs would not be extracted from surgeries. Patient identifiers such as name and date of birth would not be extracted but patients' unique NHS

For peer review only - http://bmjopen.bmj.com/site/about/guidelines.xhtml

identification number would be. GPs identified that there was a small risk of identification for patients with rare diseases (2, Pulse Today, Apr 2013).

NHS England stated that the data extracted would be crucial to integrate care and reduce treatment and care inequalities. Despite guarantees made by the government, doctors were not reassured (2, Pulse Today, Apr 2013). A GP working in Warwickshire, Dr Paul Thornton stated his concern very early in the conception of the scheme:

> *…'lots of people will want [the data, including] other Government bodies, the Department for Work and Pensions, financial institutions, pharmaceutical companies' (2, Pulse Today, Apr 2013).*

A survey carried out by Pulse Today revealed that approximately 40% of GPs wished to opt out of the system themselves, as they believed they could potentially be re-identified. An anonymous respondent stated they 'cannot see any clinical justification for the identifiable data extraction' (23, Pulse Today, Dec 2013).

Dr Marie-Louise Tidmarsh, a GP in Derbyshire, worried about being identified by her neighbours and as a result decided to opt out of the system. She said:

> *'I think patients have been misled about the "confidential" nature of the data extractions, and it is not clear to whom the data may be sold' (39, Pulse Today, Jan 2014).*

Moreover, worries about the risk of a substantial database like care.data being hacked was expressed by some doctors (54, Daily Mail, Feb 2014). This risk of hacking or re-identification was perceived to increase with the fact that unknown, and possibly profit-motivated, end-users would be provided with the data. GPs were concerned about likelihood of the data being sold to private companies (51, Daily Mail, Feb 2014).

Professor Sir Brian Jarman, a "*world specialist on hospital data*", claimed that insurance companies could identify patients in less than two hours. He explained that tracking down individuals and cross-referencing information could be fairly easy as many of these companies are already in possession of patients' names and home addresses. Sir Brian said:

> *'I've spoken to analysts who say they can match individual people within a couple of hours. Many organisations, such as insurance companies, hold details of people that include not only name but also postcode, date of birth and gender, which would make it possible to identify named individuals in postcodes and thus have access to their confidential medical*

For peer review only

information in the care.data database. There is simply too much data and the risks that something leaks are too great' (70, Mail Online, Feb 2014).

A GP in Kent, Dr Ian Williams, indicated he did not believe proposed measures would mask his identity in the data:

'I wish to opt out as I am concerned about identifiable data being moved around and passed on to third parties. I would have no objection if the data held on my records was "pseudonymised" before being extracted from the practice records' (39, Pulse Today, Jan 2014).

5. Concerns about trust between patients and doctors

GPs believed that historically, patients have trusted doctors to look after their personal data and make sure of their safety (71, London Evening Standard, Feb 2014). The doctors' relationship with their patients had always been based on trust and patients did not expect any information they share during consultations to end up in the government's database without their knowledge (25, The Independent, Jan 2014).

The fear of losing their patients' trust was one of GPs' main concerns about care.data. The BMA representatives explained that if patients were not assured of the security of their medical data, it would lead to mistrust and lack of communication by patients. This would mean doctors may not have all the information they need about the patient in the future, and patients would not share all the symptoms that are required to reach a correct diagnosis (112, BMA, Feb 2014; 100, The Daily Telegraph, Feb 2014). The Chair of the Family Doctor Association, Dr Peter Swinyard, explained:

"We have a very basic principle that whatever our patients tell us is confidential. We are completely hamstrung if patients feel they can't tell us something that - in many cases - they wouldn't tell anyone else. It is this threat to total confidentiality at consultations that gets up most GPs' noses" (37, The Independent, Jan 2014).

Professor Sue White of Birmingham University stated this problem could affect particularly vulnerable groups within society, for example women who are experiencing domestic violence issues. She believed that these women are already reluctant to open up to their GPs about their difficulties and if they doubted their data was completely confidential it might make them avoid visiting their GPs (22, The Observer, Nov 2013).

Nested within this theme was the additional distrust between doctors and the government. Because GPs did not trust the government to use patients' data safely, they could not endorse the scheme

For peer review only - http://bmjopen.bmj.com/site/about/guidelines.xhtml

and this compounded their fear of losing their patients' trust. Dr Philip Bolitho-Jones, a GP partner in Hertfordshire, stated he did not trust the politicians to decide what happened to his or his patients' medical data .

> *'We don't trust the politicians; they've got no idea what they're doing. They've already leaked before the system was even set up they were leaking data left right and centre. I wouldn't trust them with my notes; I wouldn't trust them with any of our patients' notes' (141, Pulse Today, May 2014).*

Discussion

Our results showed that GPs supported the care.data scheme in theory at the outset, and agreed that data extraction and re-use was necessary or beneficial for good research, for improving care and for saving lives. However, as time went on, they increasingly opposed certain aspects of implementation of the programme and articulated a number of key concerns. GPs cared about their patients being properly informed about the scheme. They felt the government had not informed patients adequately and could not be trusted to keep patients' data safe. They perceived that the onus was being put on them to make sure their patients were well informed, but no financial or time resources were given to them to achieve this. They disliked being scapegoated for the project's failures in communication. They also perceived that the lack of communication and guidance left them conflicted by competing obligations to share data whilst maintaining their duty of confidentiality. GPs were fearful that they could end up being held accountable under one or other of these duties.

GPs articulated a conflict over the consent mechanism in the care.data scheme. They felt that an opt-out system would be challenging for some vulnerable groups (e.g. migrants). Other literature has identified that this challenge may extend to other marginalised groups such as trans-people [28]. However, GPs understood that an opt-in system would introduce bias, and potentially reduce the quality of research. They also expressed distrust in the system proposed for protecting patient identities within the scheme, this distrust was particularly focussed on the government's history of mishandling sensitive records, and the possibility of them being sold to private companies such as insurance companies. A substantial proportion of GPs indicated they did not want their own data to be extracted, because they believed patients could be quite quickly re-identified by private companies with a profit motive. Finally, GPs feared that if patients felt their data were not

For peer review only - http://bmjopen.bmj.com/site/about/guidelines.xhtml

confidential, patients would not tell them important medical issues, and this would impede GPs' ability to provide good clinical care, and risk harming the most vulnerable patient groups.

Many of the themes expressed by GPs at the time of the care.data scheme tally with those expressed by other stakeholders, about this scheme in particular, and about patient data sharing in general. Citizens writing comments on the care.data and Guardian websites, and publishing micro-blogs on Twitter, complained of a lack of transparency and poor communication about the scheme, a lack of respect for confidentiality, misgivings about the opt-out mechanism, and fears of erosion of trust in GPs and the NHS [8, 17]. They were also concerned about commercial uses of data. Citizens could see the potential benefits of the scheme and felt that if the societal benefits and "common good" that could be derived from the scheme were made a central tenet of the scheme, [17], and meaningful public and patient involvement could be achieved [8], the scheme would have been more likely to have been accepted. Reviews of patient opinions on a range of data-sharing schemes [29, 30] have also identified similar themes underlying lay thinking on the topic. The majority of respondents to surveys and qualitative studies indicate high but qualified support of sharing patient data for research and other secondary purposes. Participants in these studies articulated fears that their privacy may be compromised and expressed distrust of particular organisations, particular those with a profit motive, which may have access to the data.

Our study additionally exposed some GP-specific issues around the implementation of care.data. GPs felt under-resourced to meet their competing duties under the HSCA 2012 and the DPA 1998. To meet their duties under these two acts, they needed to make sure patients were fully informed, but were unable to do this without any additional resource coming from the government. They also feared that data sharing schemes would detrimentally affect their relationships with their patients, leading to an erosion in trust, and leading to them being less able to care for their patients effectively.

Strengths and limitations of this study

Our approach of analysing contemporary GPs comments on care.data in published newspaper and online news articles made sure we accessed the voice of doctors during events of the unfolding crisis, and not with the hindsight of knowledge of how the situation has evolved since. The search strategy used was comprehensive and designed to include all the mainstream articles written by, quoting or written for doctors about care.data. By following a systematic approach, we minimised bias in the selection of articles for the study. However, it is possible that other sources of news, read by or authored by GPs, were not captured here, or that different opinions were being widely shared through informal GP networks and escaped our analysis. GPs may also have been quoted out of

For peer review only - http://bmjopen.bmj.com/site/about/guidelines.xhtml

context in the media to suit the purpose of the article. Additionally, those doctors contributing to mainstream news articles may not be representative of GPs in general, but instead can be viewed as key contributors to the prominent circulating discourses at the time. We acknowledge that the discourses of the quoted GPs, which were expressed in 2013-2014, may not represent their current views on the care.data scheme, its subsequent failure, or on future schemes with similar aims.

The thematic analysis was conducted by one researcher and checked carefully by a second, which may make the results more subjective and less reproducible than if a team approach had been taken. However, the themes found in our study map closely onto key themes found in other work in this field [8, 11, 17, 19], suggesting the results reported are at least consistent with stakeholder views from other sources.

Conclusions and recommendations

The study suggests that, should a similar programme be introduced in future, the support of GPs should be obtained before implementation in order to improve the success of the programme. GPs' concerns regarding confidentiality, patient information, informed consent, patient trust, need for resources, and conflicting legal obligations should be addressed during the planning phase of any data-sharing scheme, and reflected in the implementation of the programme. It appears, from subsequent national initiatives such as the development of the national data opt-out [15], that some of these lessons have already been learned. During the development of the national data opt-out, the opinions of key stakeholders and medical bodies, including doctors, were considered during the planning phase. There was effective communication with the RCGP and the Royal Colleges of Nursing and Midwifery to ensure the NHS workforce was ready across the country to start the scheme. We recommend going even further, and inviting key primary care stakeholders such as GP representatives, to sit within the teams that design and implement such schemes. Beyond consultation, we recommend a co-design approach which includes stakeholder representatives as equal team members from the outset. Examples of such good practice should be studied further, and can be combined with the results of our study, to ensure a social licence for patient data sharing is achieved with patient and clinicians, before the start of any future scheme.

Ethics approval and consent to participate

This study used publically available data from mainstream media. These data are already in the public domain. No ethical approvals were sought.

For peer review only - http://bmjopen.bmj.com/site/about/guidelines.xhtml

Consent for publication

This study used publically available data from mainstream media. These data are already in the public domain. No consent was sought.

Patient and Public Involvement

No patient involved.

Availability of data and materials'

This study used publically available data from main stream media. These data are already in the public domain.

Authors' contributions

EF, AB and SVK conceived of the presented idea. YK performed the searches, screening, data extraction and analysis with supervision from EF. MC aided interpretation of the results. EF and YK drafted the manuscript. All authors discussed results, contributed to the manuscript and approved the final version.

References

1. Hoeksma J: **The NHS's care.data scheme: what are the risks to privacy?** *BMJ : British Medical Journal* 2014, **348**:g1547.
2. **Hospital Episode Statistics (HES)** [https://digital.nhs.uk/data-and-information/data-tools-and-services/data-services/hospital-episode-statistics]; 2019.
3. Herrett E, Gallagher AM, Bhaskaran K, Forbes H, Mathur R, van Staa T, Smeeth L: **Data resource profile: clinical practice research datalink (CPRD)**. *International Journal of Epidemiology* 2015, **44**(3):827-836.
4. Lewis JD, Schinnar R, Bilker WB, Wang X, Strom BL: **Validation studies of the health improvement network (THIN) database for pharmacoepidemiology research**. *Pharmacoepidemiology and Drug Safety* 2007, **16**(4):393-401.
5. Hippisley-Cox J, Stables D, Pringle M: **QRESEARCH: a new general practice database for research**. *Journal of innovation in health informatics* 2004, **12**(1):49-50.
6. **Better information means better care** [https://www.england.nhs.uk/wp-content/uploads/2014/01/cd-leaflet-01-14.pdf]; 2014.
7. **Care.data campaign leaflet slammed** [https://www.digitalhealth.net/2014/01/care-data-campaign-leaflet-slammed/]; 2014.
8. Hays R, Daker-White G: **The care. data consensus? A qualitative analysis of opinions expressed on Twitter**. *BMC Public Health* 2015, **15**(1):838.
9. **MPS surveys of GPs and public reveal lack of information around care data** [https://www.medicalprotection.org/uk/articles/mps-surveys-of-gps-and-public-reveal-lack-of-information-around-care-data]; 2014
10. Ascherio A, Zhang SM, Hernan MA, Olek MJ, Coplan PM, Brodovicz K, Walker AM: **Hepattis B vaccinations and the risk of multiple sclerosis**. *The New England Journal of Medicine* 2001, **344**(5):327-332.
11. Presser L, Hruskova M, Rowbottom H, Kancir J: **Care. data and access to UK health records: patient privacy and public trust**. *Technology Science* 2015, **2015081103**:1-35.

For peer review only - http://bmjopen.bmj.com/site/about/guidelines.xhtml

12. Partridge N: **Data Release Review**. In.: Health and Social Care Information Centre; 2014.

13. **NHS England to close care.data programme following Caldicott Review** [http://www.nationalhealthexecutive.com/Health-Care-News/nhs-england-to-close-caredata-programme-following-caldicott-review]; 2016

14. **Review of Data Security, Consent and Opt-Outs** [https://assets.publishing.service.gov.uk/government/uploads/system/uploads/attachment_data/file/535024/data-security-review.PDF]; 2016

15. **National data opt-out** [https://digital.nhs.uk/services/national-data-opt-out-programme]; 2018

16. Ford E, Boyd A, Bowles JKF, Havard A, Aldridge RW, Curcin V, Greiver M, Harron K, Katikireddi V, Rodgers SE *et al*: **Our data, our society, our health: A vision for inclusive and transparent health data science in the United Kingdom and beyond**. *Learning Health Systems* 2019, **3**(3):e10191.

17. Sterckx S, Rakic V, Cockbain J, Borry P: **"You hoped we would sleep walk into accepting the collection of our data": controversies surrounding the UK care. data scheme and their wider relevance for biomedical research**. *Medicine, Health Care and Philosophy* 2016, **19**(2):177-190.

18. Amor BR, Vuik SI, Callahan R, Darzi A, Yaliraki SN, Barahona M: **Community detection and role identification in directed networks: understanding the twitter network of the care. data debate**. In: *Dynamic Networks and Cyber-Security.* World Scientific; 2016: 111-136.

19. Carter P, Laurie GT, Dixon-Woods M: **The social licence for research: why care. data ran into trouble**. *Journal of Medical Ethics* 2015, **41**(5):404-409.

20. Sorbie A: **Sharing confidential health data for research purposes in the UK: where are 'publics' in the public interest?** *Evidence & Policy: A Journal of Research, Debate and Practice* 2019.

21. **Care.data joint statement** [https://www.nelsonmedicalpractice.co.uk/wp-content/uploads/2015/03/caredatajointstatement1.pdf]; 2015.

22. **Care.data confidentiality concerns cannot be ignored, say doctors** [https://www.bma.org.uk/news/2014/march/caredata-confidentiality-concerns-cannot-be-ignored-say-doctors]; 2014.

23. **Data-sharing programme must include 'the profession from the outset'** [https://www.bma.org.uk/news/2016/july/data-sharing-programme-must-include-the-profession-from-the-outset]; 2016.

24. **Make care.data opt in or we will opt patients out, say GPs** [http://www.pulsetoday.co.uk/news/gp-topics/it/make-caredata-opt-in-or-we-will-opt-patients-out-say-gps/20007472.article]; 2014.

25. Moher D, Liberati A, Tetzlaff J, Altman DG: **Preferred reporting items for systematic reviews and meta-analyses: the PRISMA statement**. *Annals of internal medicine* 2009, **151**(4):264-269.

26. Braun V, Clarke V: **Using thematic analysis in psychology**. *Qualitative Research in Psychology* 2006, **3**(2):77-101.

27. **MRC Ethics Series. Using Information about People in Health Research** [https://mrc.ukri.org/documents/pdf/personal-information-in-medical-research/]; 2003.

28. Thomson RM, Katikireddi SV: **Improving the health of trans people: the need for good data**. *The Lancet Public Health* 2019, **4**(8):e369-e370.

29. Stockdale J, Cassell J, Ford E: **"Giving something back": A systematic review and ethical enquiry into public views on the use of patient data for research in the United Kingdom and the Republic of Ireland**. *Wellcome Open Res* 2019, **3**:6-6.

30. Aitken M, Jorre JdS, Pagliari C, Jepson R, Cunningham-Burley S: **Public responses to the sharing and linkage of health data for research purposes: a systematic review and thematic synthesis of qualitative studies**. *BMC Medical Ethics* 2016, **17**(1):73.

For peer review only

For peer review only - http://bmjopen.bmj.com/site/about/guidelines.xhtml

For peer review only

For peer review only - http://bmjopen.bmj.com/site/about/guidelines.xhtml

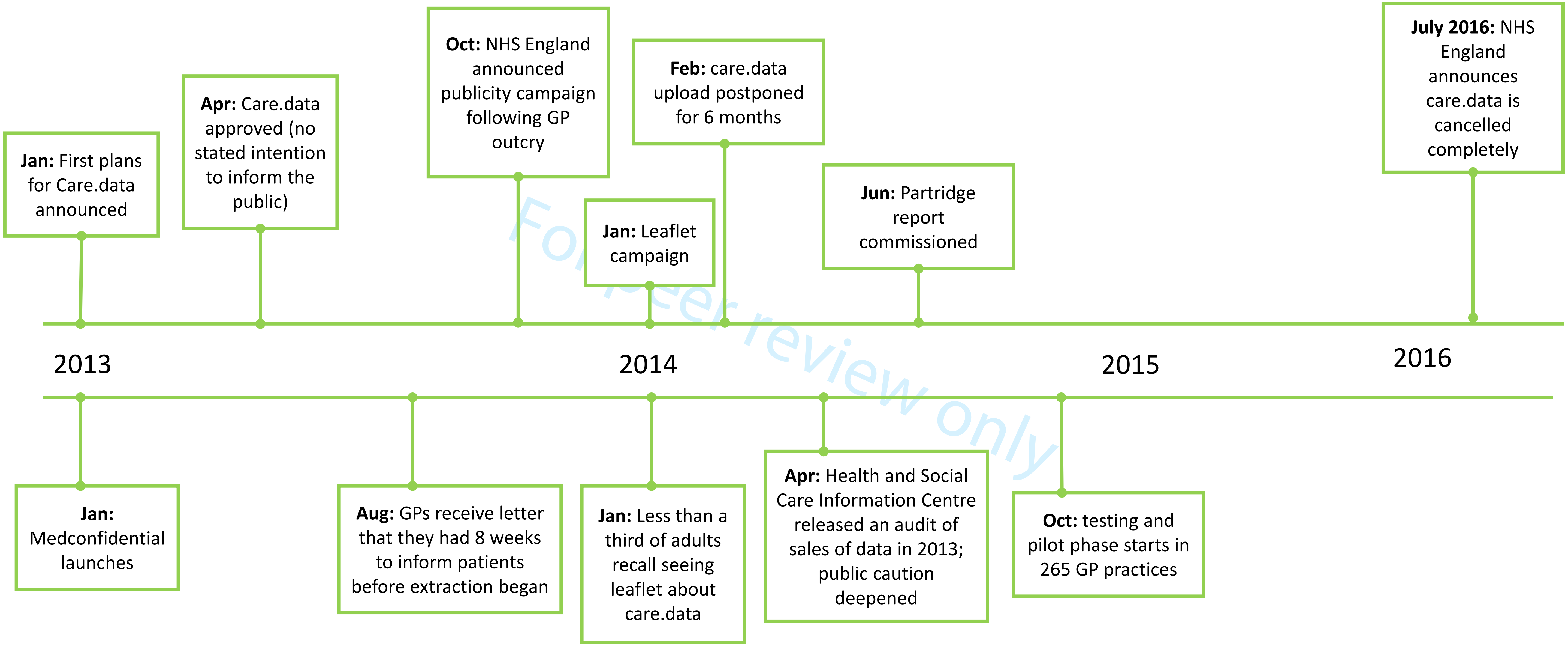
Jan: First plans for Care.data announced
Apr: Care.data approved (no stated intention to inform the public)
Oct: NHS England announced publicity campaign following GP outcry
Jan: Leaflet campaign
Feb: care.data upload postponed for 6 months
Jun: Partridge report commissioned
July 2016: NHS England announces care.data is cancelled completely
2013
2014
2015
2016
Jan: Medconfidential launches
Aug: GPs receive letter that they had 8 weeks to inform patients before extraction began
Jan: Less than a third of adults recall seeing leaflet about care.data
Apr: Health and Social Care Information Centre released an audit of sales of data in 2013; public caution deepened
Oct: testing and pilot phase starts in 265 GP practices

Newspaper articles extracted from Nexis newspaper database:

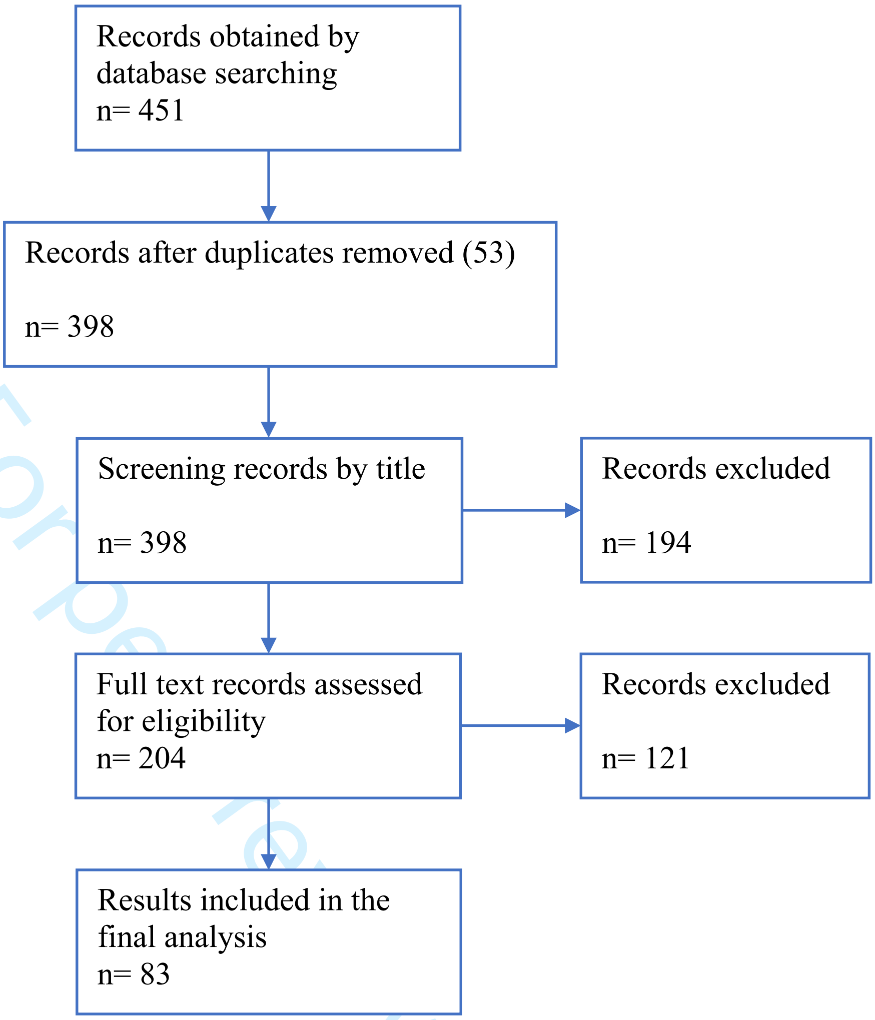

Web-based publications extracted from Nexis newspaper database:

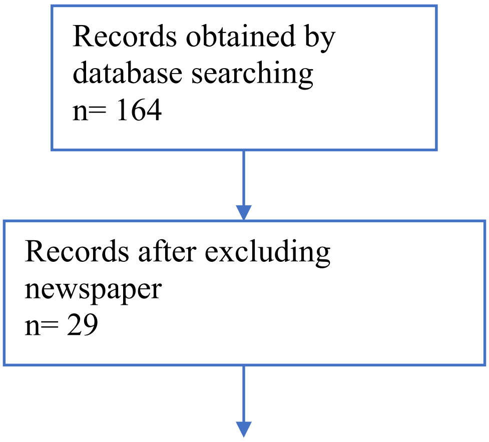

For peer review only

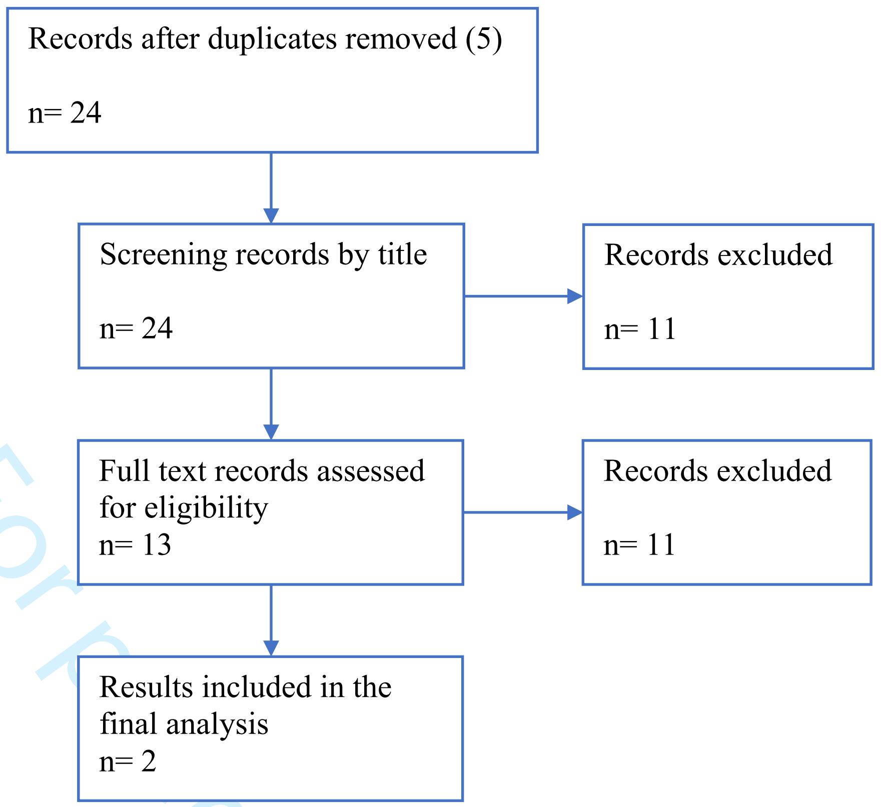

<u>BBC news articles:</u>

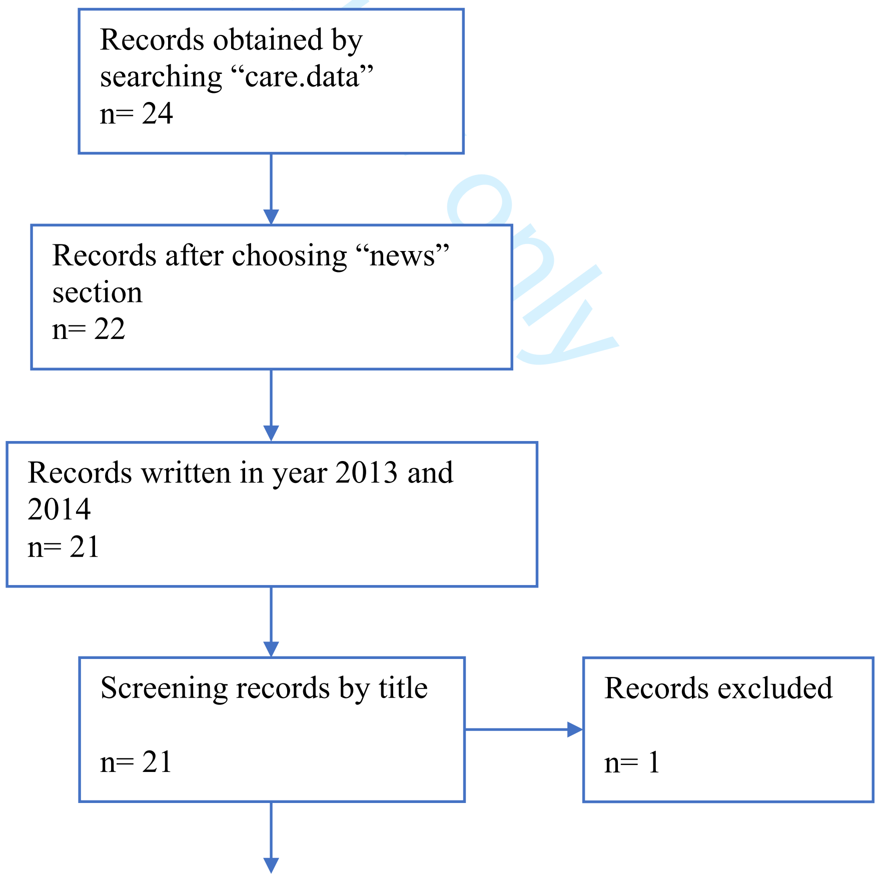

For peer review only

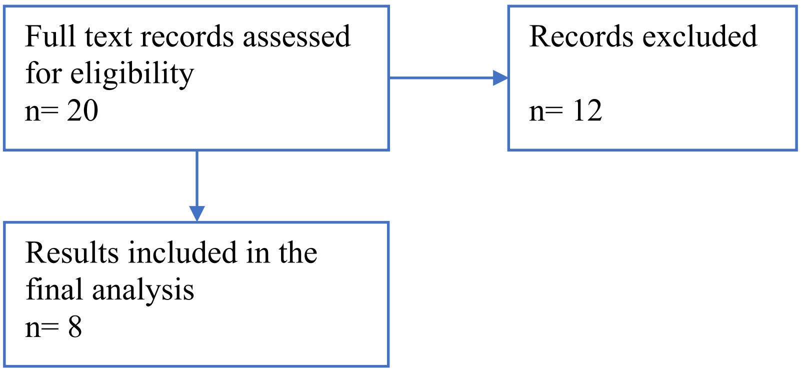

Pulse articles:

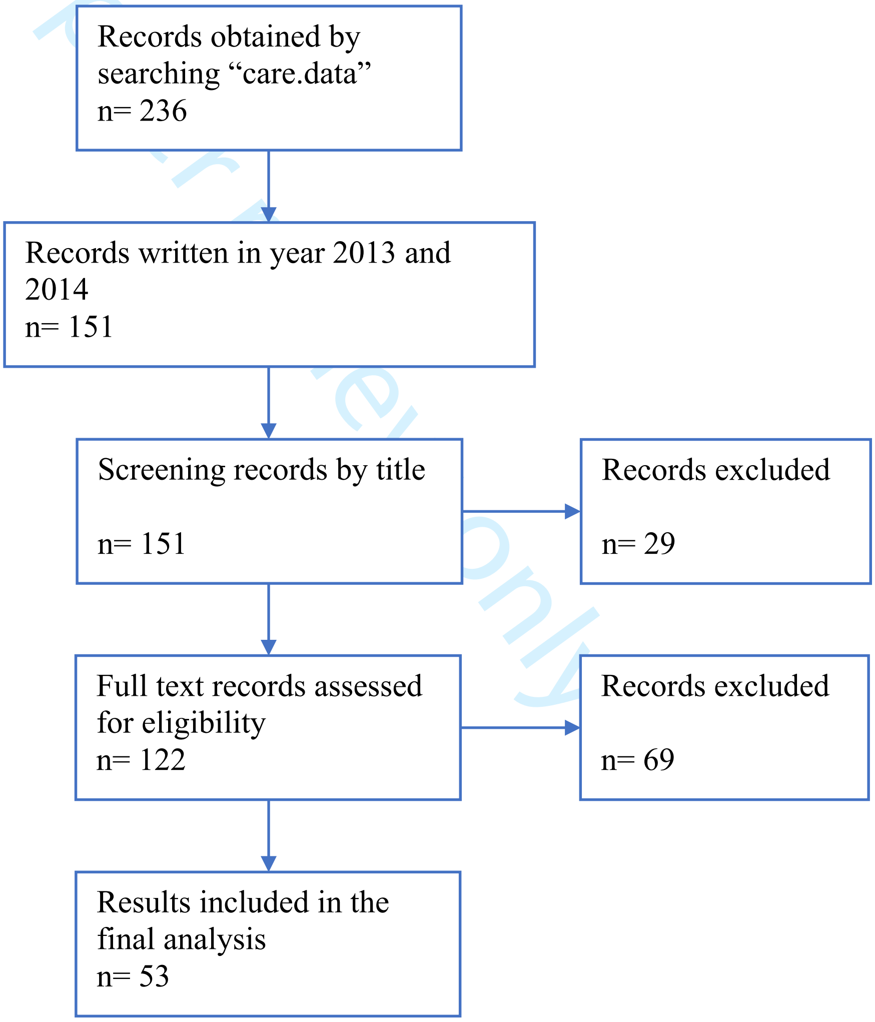

For peer review only

BMA articles:

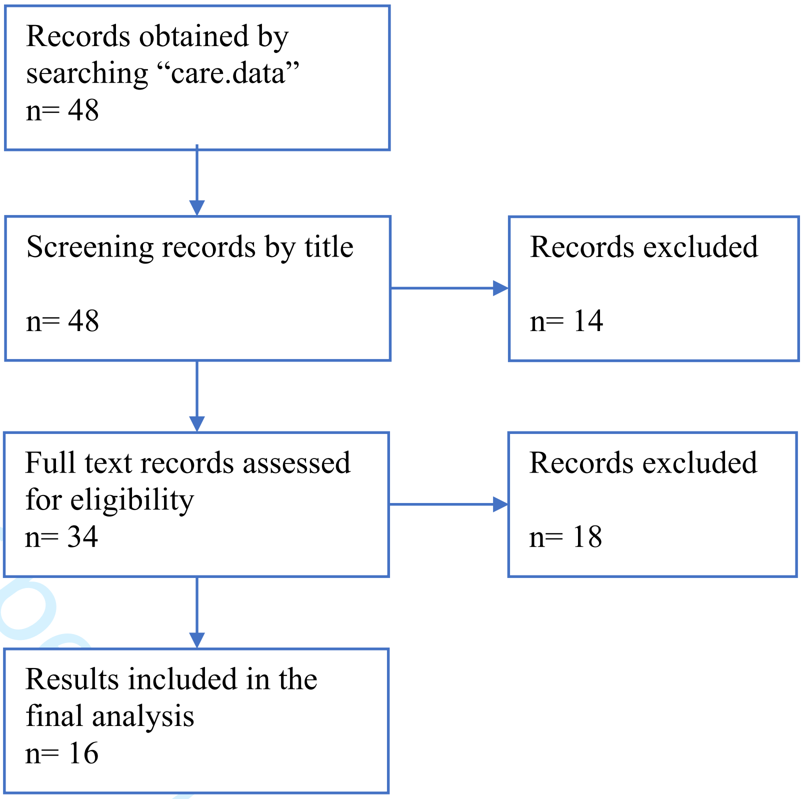

For peer review only

Appendix 1: All articles used in analysis

Article number	Source type	Source name	Article title	URL	Author's name	Date
1	GP source	Pulse Today	NHS to link up data from GP records and secondary care	http://www.pulsetoday.co.uk/news/gp-topics/it/nhs-to-link-up-data-from-gp-records-and-secondary-care/20002260.article	Madlen Davies	15/03/2013
2	GP source	Pulse Today	Risk patients will be identified by anonymised GP record data, NHS England admits	http://www.pulsetoday.co.uk/news/gp-topics/it/risk-patients-will-be-identified-by-anonymised-gp-record-data-nhs-england-admits/20002593.article	Madlen Davies	12/04/2013
3	GP source	Pulse Today	Patients to be given 'veto' over their data being shared from GP records	http://www.pulsetoday.co.uk/news/gp-topics/it/patients-to-be-given-veto-over-their-data-being-shared-from-gp-records/20002754.article	Madlen Davies	26/04/2013
4	Newspaper	The Telegraph	Database of patient information takes first steps;<br>Confidential information from medical records held by family GPs will begin to be entered into a central database from next month.	https://www.telegraph.co.uk/news/health/news/10087957/Database-of-patient-information-takes-first-steps.html	Laura Donnelly	29/05/2013
5	GP source	BMA	Information sharing guidance offered to GPs	https://www.bma.org.uk/news/2013/may/information-sharing-guidance-offered-to-gps	Not stated	29/05/2013
6	GP source	Pulse Today	GPs face prosecution unless they inform patients their data could be used outside the NHS	http://www.pulsetoday.co.uk/news/gp-topics/it/gps-face-prosecution-unless-they-inform-patients-their-data-could-be-used-outside-the-nhs/20003860.article	Madlen Davies	05/08/2013

7	GP source	Pulse Today	Private companies set for access to patient data for just £1	http://www.pulsetoday.co.uk/news/gp-topics/it/private-companies-set-for-access-to-patient-data-for-just-1/20003879.article	Madlen Davies	09/08/2013
8	GP source	BMA	New rules for doctors ensuring data confidentiality	https://www.bma.org.uk/news/2013/september/new-rules-for-doctors-ensuring-data-confidentiality	Not stated	13/09/2013
9	GP source	Pulse Today	GP leaders consider boycott of NHS England's data extraction programme	http://www.pulsetoday.co.uk/home/finance-and-practice-life-news/gp-leaders-consider-boycott-of-nhs-englands-data-extraction-programme/20004354.article	Madlen Davies	19/09/2013
10	Newspaper	Mail Online	GPs threaten to boycott 'Big Brother' NHS database which would force them to send confidential patient records to private firms	https://www.dailymail.co.uk/news/article-2428211/GPs-threaten-boycott-Big-Brother-NHS-database.html	Stephen Adams	22/09/2013
11	GP source	Pulse Today	GPs unable to object to patient data being shared, warns GPC	http://www.pulsetoday.co.uk/news/gp-topics/it/gps-unable-to-object-to-patient-data-being-shared-warns-gpc/20004458.article	Madlen Davies	26/09/2013
12	GP source	Pulse Today	NHS data extraction programme poses 'enormous threat' to privacy	http://www.pulsetoday.co.uk/news/gp-topics/it/nhs-data-extraction-programme-poses-enormous-threat-to-privacy/20004575.article	Madlen Davies	01/10/2013
13	GP source	Pulse Today	Eight weeks to inform patients their data is going to be harvested, GPs warned	http://www.pulsetoday.co.uk/news/gp-topics/it/eight-weeks-to-inform-patients-their-data-is-going-to-be-harvested-gps-warned/20004562.article	Madlen Davies	04/10/2013

14	Newspaper	The Independent	Care.data project to collate all NHS patients' records	https://www.independent.co.uk/life-style/health-and-families/health-news/caredata-project-to-collate-all-nhs-patients-records-8882379.html	Charlie Cooper	16/10/2013
15	GP source	Pulse Today	NHS England bows to confidentiality concerns and launches £2m national publicity campaign on care.data	http://www.pulsetoday.co.uk/nhs-england-bows-to-confidentiality-concerns-and-launches-2m-national-publicity-campaign-on-caredata/20004748.article	Sofia Lind	16/10/2013
16	GP source	BMA	GPs back patient-data awareness campaign	https://www.bma.org.uk/news/2013/october/gps-back-patient-data-awareness-campaign	Not stated	16/10/2013
17	Newspaper	Mail Online	NHS climbdown over 'big brother' database after the Mail on Sunday highlighted serious concerns over the plan	https://www.dailymail.co.uk/news/article-2468175/NHS-climbdown-big-brother-database-Mail-Sunday-highlighted-concerns-plan.html	Stephen Adams	19/10/2013
18	GP source	Pulse Today	Big data = big deal	http://www.pulsetoday.co.uk/views/blogs/big-data-big-deal/20004796.blog	Dr Hadrian Moss	23/10/2013
19	GP source	Pulse Today	Why I'm opting patients out of the care.data scheme	http://www.pulsetoday.co.uk/your-practice/comment/why-im-opting-patients-out-of-the-caredata-scheme/20005022.article	By one anonymous doctor	12/11/2013
20	GP source	Pulse Today	GP takes 'unlawful' decision to opt patients out of care.data programme	http://www.pulsetoday.co.uk/news/gp-topics/it/gp-takes-unlawful-decision-to-opt-patients-out-of-caredata-programme/20005018.article	Alex Matthews-King	12/11/2013

21	GP source	Pulse Today	Second GP decides to opt all patients out of records extraction as care.data rebellion grows	http://www.pulsetoday.co.uk/news/gp-topics/it/second-gp-decides-to-opt-all-patients-out-of-records-extraction-as-caredata-rebellion-grows/20005097.article	One anonymous doctor	19/11/2013
22	Newspaper	The Observer	Fears grow over open access to patient records: Confidential information could be sold to the private sector	https://www.theguardian.com/society/2013/nov/24/fears-grow-open-access-patient-records	Paul Dinsdale	24/11/2013
23	GP source	Pulse Today	Biggest risk to care.data scheme is potential loss of GP 'confidence' in benefits of scheme	http://www.pulsetoday.co.uk/news/gp-topics/it/biggest-risk-to-caredata-scheme-is-potential-loss-of-gp-confidence-in-benefits-of-scheme/20005223.article	Not stated	13/12/2013
24	GP source	BMA	Awareness of patient data sharing drive begins	https://www.bma.org.uk/news/2014/january/awareness-of-patient-data-sharing-drive-begins	Not stated	06/01/2014
25	Newspaper	The Independent	Your life in their hands: is the care.data NHS database a healthy step or a gross invasion of patient privacy?; Doctors are worried that centralising our most personal records will break the bond of trust that underpins the NHS	https://www.independent.co.uk/life-style/health-and-families/health-news/your-life-in-their-hands-is-the-caredata-nhs-database-a-healthy-step-or-a-gross-invasion-of-patient-9042564.html	Charlie Cooper	06/01/2014
26	GP source	Pulse Today	Care.data - every little helps	http://www.pulsetoday.co.uk/views/blogs/caredata-every-little-helps/20005487.blog	Dr Hadrian Moss	08/01/2014
27	GP source	Pulse Today	GPs held responsible for patient complaints over NHS data-sharing project, says ICO	http://www.pulsetoday.co.uk/news/gp-topics/it/gps-held-responsible-for-patient-complaints-over-nhs-data-sharing-project-says-ico/20005505.article	Alex Matthews-King	10/01/2014

28	Newspaper	The Daily Telegraph	Patients urged not to opt out of health database	Not available to the public	Sarah Knapton	13/01/2014
29	Newspaper	London Evening Standard	NHS data move opt-out 'damaging'	Not available to the public	Not stated	13/01/2014
30	Newspaper	The Telegraph	Health records of every NHS patient to be shared in vast database;<br>The health records of everyone in the NHS will be pooled in a vast database which can be accessed by researchers and pharmaceutical companies. But campaigners warn it could breach privacy.	https://www.telegraph.co.uk/news/10565160/Health-records-of-every-NHS-patient-to-be-shared-in-vast-database.html	Sarah Knapton	13/01/2014
31	Web publication	Sky News	NHS Data-Sharing Scheme 'Vital To Service'	https://news.sky.com/story/nhs-data-sharing-scheme-vital-to-service-10421691	Not stated	13/01/2014
32	GP source	Pulse Today	Scientists launch campaign to discourage patients from opting out of care.data	http://www.pulsetoday.co.uk/news/scientists-launch-campaign-to-discourage-patients-from-opting-out-of-caredata/20005523.article	Alex Matthews-King	13/01/2014
33	GP source	Pulse Today	Revealed: Independent experts overseeing care.data have approved 31 releases of identifiable patient data since April	http://www.pulsetoday.co.uk/news/gp-topics/it/revealed-independent-experts-overseeing-caredata-have-approved-31-releases-of-identifiable-patient-data-since-april/20005572.article	Alex Matthews-King	17/01/2014
34	Newspaper	The Guardian	Patient records to be sold from NHS database: Fears anonymous health data could be matched to insurers' medical files	Not available to the public	Randeep Rames	20/01/2014
35	Newspaper	The Daily Telegraph	Don't mislead us about our medical records; The NHS is asking us to hand over our private data - yet it cannot guarantee anonymity	https://www.telegraph.co.uk/news/nhs/10584402/Dont-mislead-us-about-our-NHS-medical-records.html	Philip Johnston	21/01/2014

36	GP source	Pulse Today	MP tables motion to halt care.data rollout as 2,400 patients call helpline	http://www.pulsetoday.co.uk/news/gp-topics/it/mp-tables-motion-to-halt-caredata-rollout-as-2400-patients-call-helpline/20005621.article	Alex Matthews-King	22/01/2014
37	Newspaper	The Independent	40 per cent of GPs plan to opt out of the NHS big data sweep, due to a lack of confidence in the project	https://www.independent.co.uk/life-style/health-and-families/health-news/40-per-cent-of-gps-plan-to-opt-out-of-the-nhs-big-data-sweep-due-to-a-lack-of-confidence-in-the-9083806.html	Charlie Cooper	24/01/2014
38	Newspaper	The Telegraph	Four in 10 GPs to opt out of NHS database; Family doctors raise concerns about new plans to share patients data	https://www.telegraph.co.uk/news/health/news/10595743/Four-in-10-GPs-to-opt-out-of-NHS-database.html	Laura Donnelly	24/01/2014
39	GP source	Pulse Today	Over 40% of GPs intend to opt themselves out of care.data scheme	http://www.pulsetoday.co.uk/news/gp-topics/it/over-40-of-gps-intend-to-opt-themselves-out-of-caredata-scheme/20005648.article	Nigel Praities	24/01/2014
40	Newspaper	i-Independent Print Ltd	GPs worried about confidentiality of patient data scheme;<br>HEALTH	Not available to the public	Charlie Cooper	25/01/2014
41	Media	BBC	Are your medical records in danger?	https://www.bbc.co.uk/news/health-25919399	Nick Triggle	28/01/2014
42	GP source	Pulse Today	Dr Amir Hannan: 'This goes to the very heart of the doctor-patient relationship'	http://www.pulsetoday.co.uk/news/gp-topics/it/dr-amir-hannan-this-goes-to-the-very-heart-of-the-doctor-patient-relationship/20005677.article	Dr Amir Hannan	28/01/2014
43	GP source	Pulse Today	Professor Roger Jones: 'I strongly support the care.data initiative'	http://www.pulsetoday.co.uk/news/gp-topics/it/professor-roger-jones-i-strongly-support-the-caredata-initiative/20005693.article	Professor Roger Jones	29/01/2014

44	GP source	Pulse Today	GP survey reveals extent of care.data ignorance among patients and staff	http://www.pulsetoday.co.uk/news/gp-topics/it/gp-survey-reveals-extent-of-caredata-ignorance-among-patients-and-staff/20005713.article	Not stated	31/01/2014
45	GP source	Pulse Today	Analysis: GPs bear brunt of care.data confusion	http://www.pulsetoday.co.uk/views/analysis/analysis-gps-bear-brunt-of-caredata-confusion/20005720.article	Not stated	31/01/2014
46	GP source	Pulse Today	No mandate for care.data grab	Not available to the public	Not stated	31/01/2014
47	Newspaper	The Guardian	We trust no one with our data - not even our doctors: The Snowden revelations could prove bad for our health, thwarting a vital NHS scheme to gather clinical information	https://www.theguardian.com/commentisfree/2014/jan/31/nhs-medical-data-trust-doctors-edward-snowden	Jonathan Freedland	01/02/2014
48	Newspaper	The Guardian	Reply: Letter: What price our medical records?	https://www.theguardian.com/society/2014/feb/02/nhs-medical-records-care-data	Dr Alex May	03/02/2014
49	GP source	Pulse Today	What's so interesting?	http://www.pulsetoday.co.uk/views/blogs/whats-so-interesting/20005728.blog	Dr Hadrian Moss	03/02/2014
50	GP source	Pulse Today	GP hit with contract notice over plan to opt all patients out of care.data	http://www.pulsetoday.co.uk/news/gp-topics/it/gp-hit-with-contract-notice-over-plan-to-opt-all-patients-out-of-caredata/20005749.article	Alex Matthews-King	04/02/2014

51	Newspaper	Daily Mail	HE OPTS HIS ENTIRE PRACTICE OUT OF SCHEME TO HARVEST MEDICAL DATA	Not available to the public	Andy Dolan And Sophie Borland	05/02/2014
52	Newspaper	Mail Online	NHS 'bullies' threaten to axe GP for keeping his patients' records private: He opts his entire practice out of scheme to harvest medical data	https://www.dailymail.co.uk/news/article-2551900/NHS-bullies-threaten-axe-GP-keeping-patients-records-private.html	Andy Dolan And Sophie Borland	05/02/2014
53	Newspaper	The Telegraph	GP fears losing job over pledge to keep patient data private;<br>A GP who pledged to keep his patients' confidential medical information private fears he could be ousted from his practice after NHS bosses said he could be found in breach of contract if he opts his patients out of the scheme	https://www.telegraph.co.uk/news/nhs/10618814/GP-fears-losing-job-over-pledge-to-keep-patient-data-private.html	Claire Carter	05/02/2014
54	Newspaper	Daily Mail	I won't give in to the NHS thought police who want to sell your private medical records	https://www.dailymail.co.uk/debate/article-2552717/I-wont-NHS-Thought-Police-want-sell-private-medical-records-GP-threatened-health-chiefs-hits-back.html	Dr Gordon Gancz	06/02/2014
55	GP source	Pulse Today	Saying no to care.data = saying no to care?	http://www.pulsetoday.co.uk/saying-no-to-caredata-saying-no-to-care/20005801.blog	Dr Tony Copperfield	07/02/2014
56	Newspaper	Daily Mail	INSURERS COULD USE NEW NHS DATABASE TO TRACK YOU DOWN WITHIN 2 HOURS'	https://www.dailymail.co.uk/news/article-2554437/Insurers-use-new-NHS-database-track-two-hours.html	Sophie Borland	08/02/2014
57	Newspaper	The Times	Share your data with the NHS. It's safe and beneficial;<br>Patient information will be secure in a confidential system and will be used only appropriately	https://www.thetimes.co.uk/article/share-your-data-with-the-nhs-its-safe-and-beneficial-cjmzw36s0jp	Dr Clare Gerada	08/02/2014

58	Newspaper	Mail Online	Big Brother' database will grab children's health records but parents are being kept in the dark	https://www.dailymail.co.uk/news/article-2554959/Big-Brother-database-grab-childrens-health-records-parents-kept-dark.html	Martin Beckford	09/02/2014
59	Newspaper	The Telegraph	The brave doctor taking on Big Brother;<br>We owe a debt to the family GP who refuses to give up his patients' medical records to the Health and Social Care Information Centre	https://www.telegraph.co.uk/news/nhs/10627492/The-brave-doctor-taking-on-Big-Brother.html	Max Pemberton	10/02/2014
60	Newspaper	The Telegraph	Crisis of confidence' in NHS database, warn GPs;<br>Stall NHS data-sharing amid 'crisis of confidence' GPs say	https://www.telegraph.co.uk/news/health/news/10634539/Crisis-of-confidence-in-NHS-database-warn-GPs.html	Laura Donnelly	12/02/2014
61	Media	BBC	GPs voice fears over giant patient records database	https://www.bbc.co.uk/news/health-26151458	Nick Triggle	12/02/2014
62	GP source	Pulse Today	RCGP demands new publicity campaign to address 'crisis in confidence' over care.data scheme	http://www.pulsetoday.co.uk/news/gp-topics/it/rcgp-demands-new-publicity-campaign-to-address-crisis-in-confidence-over-caredata-scheme/20005847.article	Alex Matthews-King	12/02/2014
63	Newspaper	Daily Mail	GPS WARN OF CRISIS IN PUBLIC CONFIDENCE OVER NHS DATABASE	https://www.dailymail.co.uk/news/article-2558135/GPs-warn-crisis-public-confidence-NHS-database-Royal-Collage-warns-health-service-failing-inform-patients-data-sharing.html	Sophie Borland	13/02/2014
64	Newspaper	The Times	GPs fear failure of patient data scheme	https://www.thetimes.co.uk/article/gps-fear-failure-of-patient-data-scheme-6csvc9wj3l2	Chris Smyth	13/02/2014

65	GP source	Pulse Today	Two-thirds of public 'don't recall receiving care.data information leaflet'	http://www.pulsetoday.co.uk/news/gp-topics/it/two-thirds-of-public-dont-recall-receiving-caredata-information-leaflet/20005874.article	Alex Matthews-King	14/02/2014
66	Newspaper	Mail Online	Two-thirds oppose plans for new NHS database that will see confidential medical records sold to private firms	https://www.dailymail.co.uk/news/article-2560335/Two-thirds-oppose-plans-new-NHS-database-confidential-medical-records-sold-private-firms.html	Not stated	15/02/2014
67	Newspaper	The Daily Telegraph	NHS admits database could pose privacy risk; NHS database could be disaster, say campaingers	https://www.telegraph.co.uk/news/nhs/10642740/NHS-admits-new-medical-records-database-could-pose-privacy-risk.html	Laura Donnelly	17/02/2014
68	Newspaper	The Independent	Your life in their hands: is the care.data NHS database a healthy step or a gross invasion of patient privacy?;<br>Doctors are worried that centralising our most personal records will break the bond of trust that underpins the NHS	https://www.independent.co.uk/life-style/health-and-families/health-news/your-life-in-their-hands-is-the-caredata-nhs-database-a-healthy-step-or-a-gross-invasion-of-patient-9042564.html	Charlie Cooper	17/02/2014
69	Newspaper	The Independent	Doctors raise fears over sharing NHS patient records;<br>BMA 'deeply concerned' with Government's public information campaign for care.data project	https://www.independent.co.uk/life-style/health-and-families/health-news/doctors-raise-fears-over-sharing-nhs-patient-records-9133807.html	Jane Kirby, Ella Pickover	17/02/2014
70	Newspaper	Mail Online	Now doctors' union has 'grave doubts' over plan to harvest patient data: British Medical Association warns public has been left in the dark over the scheme	https://www.dailymail.co.uk/news/article-2561662/Now-doctors-union-grave-doubts-plan-harvest-patient-data-British-Medical-Association-warns-public-left-dark-scheme.html	Sophie Borland	17/02/2014
71	Newspaper	London Evening Standard	Concern at medical records sharing	Not available to the public	Not stated	17/02/2014

72	GP source	Pulse Today	GPC calls for urgent talks over public awareness of care.data scheme	http://www.pulsetoday.co.uk/news/gp-topics/it/gpc-calls-for-urgent-talks-over-public-awareness-of-caredata-scheme/20005884.article	Alex Matthews-King	17/02/2014
73	GP source	BMA	Call to boost public awareness of data sharing	https://www.bma.org.uk/news/2014/february/call-to-boost-public-awareness-of-data-sharing	Not stated	17/02/2014
74	Newspaper	The Daily Telegraph	Delay NHS database until public is dear, say experts	Not available to the public	Laura Donnelly	18/02/2014
75	Newspaper	Daily Mail	NOW DOCTORS' UNION HAS GRAVE DOUBTS' OVER PLAN TO HARVEST PATIENT DATA	https://www.dailymail.co.uk/news/article-2561662/Now-doctors-union-grave-doubts-plan-harvest-patient-data-British-Medical-Association-warns-public-left-dark-scheme.html	Sophie Borland	18/02/2014
76	Newspaper	The Guardian	NHS in England delays sharing of medical records	https://www.theguardian.com/society/2014/feb/18/nhs-delays-sharing-medical-records-care-data	Peter Walker	18/02/2014
77	Newspaper	The Independent	Victory for privacy as NHS database is delayed;<br>Collection of confidential medical records put on hold after backlash. Critics feared personal information would be vulnerable to hackers	https://www.independent.co.uk/life-style/health-and-families/health-news/victory-for-privacy-as-nhs-database-is-delayed-9137136.html	Charlie Cooper	18/02/2014
78	Newspaper	The Independent	Doctors raise fears over sharing NHS patient medical records;<br>BMA 'deeply concerned' with Government's public information campaign for care.data project	https://www.independent.co.uk/life-style/health-and-families/health-news/doctors-raise-fears-over-sharing-nhs-patient-records-9133807.html	Jane Kirby, Ella Pickover	18/02/2014
79	Newspaper	Mail Online	Controversial plan to share medical records across<br>NHS is put on hold for six months	Not available to public	Sam Webb	18/02/2014

80	Newspaper	London Evening Standard	Medical records share plan delayed	https://www.standard.co.uk/panewsfeeds/medical-records-share-plan-delayed-9136528.html	Not stated	18/02/2014
81	Newspaper	London Evening Standard	Records plan clarification urged	Not available to the public	Not stated	18/02/2014
82	Newspaper	The Telegraph	Patients should be warned before NHS shares medical records, doctors say; Growing pressure on ministers to go back to drawing board on NHS medical records	https://www.telegraph.co.uk/news/health/news/10646151/Patients-should-be-warned-before-NHS-shares-medical-records-doctors-say.html	Laura Donnelly	18/02/2014
83	Newspaper	The Times	Health chiefs delay data-sharing plan in big climbdown	https://www.thetimes.co.uk/article/nhs-chiefs-in-climbdown-over-sharing-patient-data-66396lf825q	Chris Smyth	18/02/2014
84	Web publication	Sky News	NHS Records Database Postponed Amid Concerns	https://news.sky.com/story/nhs-records-database-postponed-amid-concerns-10416834	Not stated	18/02/2014
85	Media	BBC	Giant NHS database rollout delayed	https://www.bbc.co.uk/news/health-26239532	Nick Triggle	18/02/2014
86	GP source	Pulse Today	NHS England delays care.data scheme to 'build understanding' of benefits	http://www.pulsetoday.co.uk/news/gp-topics/it/nhs-england-delays-caredata-scheme-to-build-understanding-of-benefits/20005896.article	Alex Matthews-King	18/02/2014
87	Newspaper	The Daily Telegraph	NHS database put on hold as officials accept privacy fears	Not available to the public	Laura Donnelly	19/02/2014

88	Newspaper	Daily Mail	HOW CONCERNS GREW	Not available to the public	Sophie Borland	19/02/2014
89	Newspaper	The Guardian	NHS health record database delayed after public outcry: New system put on hold for at least six months: Critics say NHS England has failed to put case for it	Not available to the public	Peter Walker, James Meikle and Randeep Ramesh	19/02/2014
90	Newspaper	The Independent	Victory for privacy as NHS database delayed; * Collection of confidential medical records put on hold after backlash * Critics feared personal information would be vulnerable to hackers Data-sharing delay gives NHS 'time to get it right'	https://www.independent.co.uk/life-style/health-and-families/health-news/victory-for-privacy-as-nhs-database-is-delayed-9137136.html	Charlie Cooper	19/02/2014
91	Newspaper	Mail Online	NHS delays plan to harvest your details: Victory for the Mail as database is shelved for six months	https://www.dailymail.co.uk/news/article-2562296/Controversial-plan-share-medical-records-NHS-hold-six-months.html	Sophie Borland	19/02/2014
92	Newspaper	The Telegraph	NHS database 'vital' to improve cancer research; Medical records database 'vital' for improving the country's poor cancer survival record, NHS chief says	https://www.telegraph.co.uk/news/health/news/10649786/NHS-database-vital-to-improve-cancer-research.html	Laura Donnelly	19/02/2014
93	Newspaper	The Times	NHS chiefs in climbdown over sharing patient data	https://www.thetimes.co.uk/article/nhs-chiefs-in-climbdown-over-sharing-patient-data-66396lf825q	Chris Smyth	19/02/2014
94	GP source	Pulse Today	Rebel GP told contract notice over care.data opt-outs was a 'misunderstanding'	http://www.pulsetoday.co.uk/news/gp-topics/it/rebel-gp-told-contract-notice-over-caredata-opt-outs-was-a-misunderstanding/20005908.article	Alex Matthews-King	19/02/2014

95	GP source	BMA	Doctors' concerns prompt data sharing plan delay	https://www.bma.org.uk/news/2014/february/doctors-concerns-prompt-data-sharing-plan-delay	Not stated	19/02/2014
96	Newspaper	The Daily Telegraph	Database a fundamental part of the fight against cancer, says NHS chief;<br>Director defends scheme as crucial to future of the health service	Not available to the public	Laura Donnelly	20/02/2014
97	Newspaper	The Times	NHS must quell fears over data or lives will be lost, says charity	https://www.thetimes.co.uk/article/nhs-must-quell-fears-over-data-or-lives-will-be-lost-says-charity-z5rj2m7qwhc	Michael Savage and Chris Smyth	20/02/2014
98	Media	BBC	Critics of giant NHS database 'are scaremongering'	https://www.bbc.co.uk/news/health-26277866	Nick Triggle and Adam Brimelow	21/02/2014
99	GP source	BMA	GPC Newsletter	https://www.bma.org.uk/connecting-doctors/the_practice/m/mediagallery/24	Not stated	21/02/2014
100	Newspaper	The Daily Telegraph	Data don't help cancer;<br>Letters to the Editor	Not available to public	Dr Neil Bhatia	22/02/2014
101	Newspaper	The Telegraph	NHS medical records database could help prevent disease, senior doctors say;<br>Lord Darzi and Lord Crisp have joined a group of health professionals to defend the NHS medical records database, days after it was put on hold following concerns about data sharing	https://www.telegraph.co.uk/news/health/news/10657580/NHS-medical-records-database-could-help-prevent-disease-senior-doctors-say.html	Claire Carter	24/02/2014
102	Newspaper	The Times	Healthcare data;<br>If data linkage in the UK had not been undertaken in the past we would know less about the causes of disease and be ignorant of inequities in access to care	Not available to the public	Not stated	24/02/2014

103	Newspaper	The Times	Medical advances need sharing of data, say doctors	https://www.thetimes.co.uk/article/medical-advances-need-sharing-of-data-say-doctors-s6flk6l6dn2	Oliver Moody	24/02/2014
104	Media	BBC	NHS data-sharing project at risk, say MPs	https://www.bbc.co.uk/news/health-26347026	Nick Triggle	25/02/2014
105	Media	BBC	NHS data-sharing will have 'enormous benefits', say peers	http://www.bbc.co.uk/democracylive/house-of-lords-26343037	Not stated	25/02/2014
106	GP source	Pulse Today	Debate: Should GPs back care.data?	http://www.pulsetoday.co.uk/news/gp-topics/it/debate-should-gps-back-caredata/20005959.article	Not stated	25/02/2014
107	Newspaper	The Independent	Atos to manage NHS care.data project despite ongoing 'mess' over disability benefit assessments; Despite the Disability Minister Mike Penning admitting the reassessments were in 'chaos' Atos will roll out the controversial care.data project	https://www.independent.co.uk/news/atos-to-work-on-nhs-care-data-project-despite-ongoing-mess-over-disability-benefit-assessments-9153885.html	Felicity Morse	26/02/2014
108	Newspaper	The Times	GPs force rethink on data-sharing plan;	https://www.thetimes.co.uk/article/gps-force-rethink-on-data-sharing-plan-hlkzfj96qzs	Michael Savage	26/02/2014
109	Newspaper	The Times	The pitfalls of the NHS central database plan; Letters to the Editor	https://www.thetimes.co.uk/article/the-pitfalls-of-the-nhs-central-database-plan-qts55x7pdjq	Not stated	26/02/2014
110	Newspaper	The Times	Opt-out easier in GP data-sharing rethink	Not available to the public	Michael Savage	26/02/2014

111	GP source	Pulse Today	Three in four GPs believe care.data should be 'opt in'	http://www.pulsetoday.co.uk/news/gp-topics/it/three-in-four-gps-believe-caredata-should-be-opt-in/20005954.article	Alex Matthews-King	26/02/2014
112	GP source	BMA	Patient trust threatened by data-sharing concerns	https://www.bma.org.uk/news/2014/february/patient-trust-threatened-by-data-sharing-concerns	Not stated	26/02/2014
113	Newspaper	The Daily Telegraph	GPs back 'opt-in' NHS database	Not available to the public	Not stated	27/02/2014
114	Newspaper	The Independent	Atos to work on NHS care data project despite ongoing 'mess' over disability benefit assessments;<br>Despite the Disability Minister Mike Penning admitting the reassessments were in 'chaos' Atos will roll out the controversial care.data project	https://www.independent.co.uk/news/atos-to-work-on-nhs-care-data-project-despite-ongoing-mess-over-disability-benefit-assessments-9153885.html	Felicity Morse	27/02/2014
115	Newspaper	Mail Online	Cashing in on patient records to be banned: But you'll still have to opt out to keep private details off database	https://www.dailymail.co.uk/news/article-2570567/Cashing-patient-records-banned-But-youll-opt-private-details-database.html	James Chapman and Andy Dolan	28/02/2014
116	GP source	Pulse Today	Update: Care.data put on hold	http://www.pulsetoday.co.uk/views/analysis/update-caredata-put-on-hold/20005997.article	Nigel Praities	28/02/2014
117	Newspaper	The Daily Telegraph	NHS data for sale: will your premiums rise?; Insurance Nicole Blackmore examines the Government's plans for a new database and how it could be exploited by insurers	Not available to the public	Nicole Blackmore	01/03/2014
118	Newspaper	Daily Mail	CASHING IN ON PATIENT RECORDS TO BE BANNED	https://www.dailymail.co.uk/news/article-2570567/Cashing-patient-records-banned-But-youll-opt-private-details-database.html	James Chapman And Andy Dolan	01/03/2014

119	Newspaper	London Evening Standard	NHS data will not be sold: Hunt	https://www.standard.co.uk/panewsfeeds/nhs-data-will-not-be-sold-hunt-9161694.html	Not stated	01/03/2014
120	Newspaper	The Times	NHS patient data to be made confidential	https://www.thetimes.co.uk/article/nhs-patient-data-to-be-made-confidential-gdxmctdnh56	Tom Whipple	01/03/2014
121	GP source	Pulse Today	Legislation will prevent patient data being sold outside the health service, says Hunt	http://www.pulsetoday.co.uk/news/gp-topics/it/legislation-will-prevent-patient-data-being-sold-outside-the-health-service-says-hunt/20006014.article	Alex Matthews-King	03/03/2014
122	Newspaper	The Daily Telegraph	Hospital records used by private marketing firms;<br>NHS data helps firms 'tailor media campaigns'	https://www.telegraph.co.uk/news/nhs/10674639/NHS-hospital-records-used-by-private-marketing-firms.html	Laura Donnelly	04/03/2014
123	Newspaper	Mail Online	BY THE WAY . . . GP RECORDS DON'T TELL THE WHOLE STORY	Not available to the public	Dr Martin Scurr	04/03/2014
124	Newspaper	The Guardian	Reply: Letter: Taking better care of health data	https://www.theguardian.com/society/2014/mar/04/taking-better-care-health-data	Various<br>Prof Harvey Goldstein University of Bristol, Prof Ruth Gilbert University College London, Dr Katie Harron UCL, Dr Gareth Hagger-Johnson UCL, Dr Mario Cortina UCL ,	05/03/2014

					Dr Nirupa Dattani City University	
125	Newspaper	The Guardian	Reply: Letter: Taking better care of health data	https://www.theguardian.com/society/2014/mar/04/taking-better-care-health-data	Professor Robert Boyd	05/03/2014
126	Newspaper	The Telegraph	NHS database: will it push up your insurance premiums?; Experts fear insurers will buy medical information about customers and use it to price policies	https://www.telegraph.co.uk/finance/personalfinance/insurance/10667245/NHS-database-will-it-push-up-your-insurance-premiums.html	Nicole Blackmore	05/03/2014
127	GP source	Pulse Today	Falling off the care.data fence	http://www.pulsetoday.co.uk/views/blogs/falling-off-the-caredata-fence/20006012.blog	Not stated	05/03/2014
128	GP source	BMA	GPC Newsletter	Not available to the public<br><br>file:///Users/yaldakazempour/Downloads/11%20GPC%20Newsletter%2006%2003%2014%20(3).pdf	Not stated	06/03/2014
129	Newspaper	The Times	Health data centre pledges more honesty	https://www.thetimes.co.uk/article/health-data-centre-pledges-more-honesty-m7qpfbtx8qf	Chris Smyth	07/03/2014
130	GP source	BMA	BMA remains concerned over special measures safeguards	https://www.bma.org.uk/news/2014/march/bma-remains-concerned-over-special-measures-safeguards	Not stated	07/03/2014
131	Media	BBC	Government outlines data-sharing safeguards	http://www.bbc.co.uk/democracylive/house-of-commons-26518046	Not stated	10/03/2014

132	Media	BBC	MPs agree to data-sharing safeguards	http://www.bbc.co.uk/democracylive/house-of-commons-26532173	Not stated	11/03/2014
133	Newspaper	The Times	Sharing medical data would turn UK into 'best clinical laboratory in world'	https://www.thetimes.co.uk/article/sharing-medical-data-would-turn-uk-into-best-clinical-laboratory-in-world-sg639w8m5cj	Chris Smyth	21/03/2014
134	GP source	Pulse Today	Patients would 'embrace' care.data if they understood it, jet lag cure, crackdown on slum landlords and exploding cells to fight cancer	http://www.pulsetoday.co.uk/patients-would-embrace-caredata-if-they-understood-it-jet-lag-cure-crackdown-on-slum-landlords-and-exploding-cells-to-fight-cancer/20006171.article	Christian Duffin	21/03/2014
135	GP source	BMA	Care.data confidentiality concerns cannot be ignored, say doctors	https://www.bma.org.uk/news/2014/march/caredata-confidentiality-concerns-cannot-be-ignored-say-doctors	Dr Lucy-Jane Davis	21/03/2014
136	GP source	BMA	GPC Newsletter	Not available to the public	Not stated	21/03/2014
137	Newspaper	The Independent	Medical chief defends NHS data-sharing; HEALTH	Not available to the public	Paul Bignell	22/03/2014
138	Newspaper	Daily Mail	MORE DELAYS FOR NHS DATA HARVESTING PROGRAMME	https://www.dailymail.co.uk/news/article-2608134/More-delays-controversial-NHS-data-harvesting-programme-Government-adviser-says-scheme-mishandled.html	Not stated	19/04/2014

139	GP source	BMA	GPC Newsletter	https://www.bma.org.uk/connecting-doctors/the_practice/m/mediagallery/48		22/04/2014
140	Newspaper	The Daily Telegraph	Patient data in peril;<br>Letters to the Editor	Not available to the public	Professor John R Ashton	07/05/2014
141	GP source	Pulse Today	Practice opts all patients out of care.data despite delay to roll-out	http://www.pulsetoday.co.uk/news/gp-topics/it/practice-opts-all-patients-out-of-caredata-despite-delay-to-roll-out/20006682.article	Not stated	12/05/2014
142	Newspaper	The Telegraph	Britons 'trust banks more than government' to protect their data;<br>The government has much work to do reassuring the public of its data security measures, according to Unisys	https://www.telegraph.co.uk/technology/internet-security/10846656/Britons-trust-banks-more-than-government-to-protect-their-data.html	Sophie Curtis	21/05/2014
143	GP source	Pulse Today	GPs vote in favour of an opt-in system for care.data	http://www.pulsetoday.co.uk/news/gp-topics/it/gps-vote-in-favour-of-an-opt-in-system-for-caredata/20006796.article	Alex Matthews-King	23/05/2014
144	GP source	BMA	Patient data extraction requires explicit consent, say GPs	https://www.bma.org.uk/news/2014/may/patient-data-extraction-requires-explicit-consent-say-gps	Not stated	27/05/2014
145	Newspaper	The Times	GPs force rethink on data-sharing plan	https://www.thetimes.co.uk/article/gps-force-rethink-on-data-sharing-plan-hlkzfj96qzs	Michael Savage	17/06/2014
146	Newspaper	Daily Mail	AND OFFICIALS DON'T EVEN KNOW WHERE DETAILS OF 1.3M PATIENTS ENDED UP	Not available to the public	Sophie Borland	18/06/2014

147	GP source	Pulse Today	Parts of care.data should be 'opt-in' only, says NHS England director	http://www.pulsetoday.co.uk/news/gp-topics/it/parts-of-caredata-should-be-opt-in-only-says-nhs-england-director/20007039.article	Sofia Lind	20/06/2014
148	Newspaper	Mail Online	CONSENT CONCERNS ON MEDICAL RECORDS	Not available to the public	Not stated	25/06/2014
149	GP source	Pulse Today	Over half the public still unaware of care.data	http://www.pulsetoday.co.uk/news/gp-topics/it/over-half-the-public-still-unaware-of-caredata/20007087.article	Alex Matthews-King	25/06/2014
150	GP source	BMA	Patients’ medical data sacrosanct, declares BMA	https://www.bma.org.uk/news/2014/june/patients-medical-data-sacrosanct-declares--bma	Not stated	25/06/2014
151	GP source	Pulse Today	GPs to send patient letters explaining care.data 'opt-out'	http://www.pulsetoday.co.uk/news/gp-topics/it/gps-to-send-patient-letters-explaining-caredata-opt-out/20007191.article	Alex Matthews-King	03/07/2014
152	GP source	BMA	Call to involve patients in how data is shared	https://www.bma.org.uk/news/2014/july/call-to-involve-patients-in-how-data-is-shared	Not stated	03/07/2014
153	GP source	Pulse Today	Make care.data opt in or we will opt patients out, say GPs	http://www.pulsetoday.co.uk/news/gp-topics/it/make-caredata-opt-in-or-we-will-opt-patients-out-say-gps/20007472.article	Alex Matthews-King	05/08/2014
154	GP source	Pulse Today	GPs' care.data responsibilities amount to 'good customer service', says information commissioner	http://www.pulsetoday.co.uk/news/gp-topics/it/gps-caredata-responsibilities-amount-to-good-customer-service-says-information-commissioner/20007450.article	Alex Matthews-King	05/08/2014

155	GP source	Pulse Today	Police should have greater access to medical records, says chief constable	http://www.pulsetoday.co.uk/news/gp-topics/it/police-should-have-greater-access-to-medical-records-says-chief-constable/20007529.article	Alex Matthews-King	11/08/2014
156	GP source	Pulse Today	NHS England looks at expanding care.data extractions to include 'sensitive' patient information	http://www.pulsetoday.co.uk/news/gp-topics/it/nhs-england-looks-at-expanding-caredata-extractions-to-include-sensitive-patient-information/20007611.article	Alex Matthews-King	19/08/2014
157	GP source	Pulse Today	Selling patient data poses risks to GPs and patients alike	http://www.pulsetoday.co.uk/views/blogs/inside-information/selling-patient-data-poses-risks-to-gps-and-patients-alike/20007916.blog	Dr Hadrian Moss	17/09/2014
158	GP source	Pulse Today	GPs could opt all patients out of care.data opt under data protection laws, information office says	http://www.pulsetoday.co.uk/news/gp-topics/it/gps-could-opt-all-patients-out-of-caredata-opt-under-data-protection-laws-information-office-says/20008114.article	Alex Matthews-King	06/10/2014
159	Newspaper	Mail Online	Storm as NHS gives go-ahead to patient database despite concerns: Pilot scheme will involve 1.7million people unless an individual specifically ops out	https://www.dailymail.co.uk/news/article-2784269/Storm-NHS-gives-ahead-patient-database-despite-concerns-Pilot-scheme-involve-1-7million-people-unless-individual-specifically-ops-out.html	Sophie Borland	07/10/2014
160	GP source	Pulse Today	GPs to contact patients individually to explain care.data opt-out	http://www.pulsetoday.co.uk/news/gp-topics/it/gps-to-contact-patients-individually-to-explain-caredata-opt-out/20008134.article	Alex Matthews-King	07/10/2014

161	Newspaper	Mail Online	NHS data is snooped on six times every day: Staff caught looking at records of friends, family and even love rivals	https://www.dailymail.co.uk/news/article-2833960/NHS-data-snooped-six-times-day-Staff-caught-looking-records-friends-family-love-rivals.html?ITO=1490&ns_mchannel=rss&ns_campaign=1490	Sophie Borland	14/11/2014
162	GP source	Pulse Today	Care.data extractions on hold until NHS England satisfies GP concerns	http://www.pulsetoday.co.uk/news/gp-topics/it/caredata-extractions-on-hold-until-nhs-england-satisfies-gp-concerns/20008789.article	Alex Matthews-King	18/12/2014

peer review only

Enhancing transparency in reporting the synthesis of qualitative research: the ENTREQ statement

No	Item	Description	Page
1	Aim	State the research question the synthesis addresses	5
2	Synthesis Methodology	Identify the synthesis methodology or theoretical framework which underpins the synthesis, and describe the rationale for choice of methodology (e.g. meta-ethnography, thematic synthesis, critical interpretive synthesis, grounded theory synthesis, realist synthesis, meta-aggregation, meta-study, framework synthesis).	7
3	Approach to searching	Indicate whether the search was pre-planned (*comprehensive search strategies to seek all available studies)* or iterative (*to seek all available concepts until they theoretical saturation is achieved)*.	6
4	Inclusion criteria	Specify the inclusion/exclusion criteria *(e.g. in terms of population, language, year limits, type of publication, study type).*	6
5	Data sources	Describe the information sources used (e.g. *electronic databases (MEDLINE, EMBASE, CINAHL, psycINFO, Econlit), grey literature databases (digital thesis, policy reports), relevant organisational websites, experts, information specialists, generic web searches (Google Scholar) hand searching, reference lists)* and when the searches conducted; provide the rationale for using the data sources.	6
6	Electronic search strategy	Describe the literature search *(e.g. provide electronic search strategies with population terms, clinical or health topic terms, experiential or social phenomena related terms, filters for qualitative research, and search limits).*	6
7	Study screening methods	Describe the process of study screening and sifting *(e.g. title, abstract and full text review, number of independent reviewers who screened studies).*	6
8	Study characteristics	Present the characteristics of the included studies *(e.g. year of publication, country, population, number of participants, data collection, methodology, analysis, research questions).*	Table 2
9	Study selection results	Identify the number of studies screened and provide reasons for study exclusion *(e.g. for comprehensive searching, provide numbers of studies screened and reasons for exclusion indicated in a figure/flowchart; for iterative searching describe reasons for study exclusion and inclusion based on modifications, the research question and/or contribution to theory development).*	7-8
10	Rationale for appraisal	Describe the rationale and approach used to appraise the included studies or selected findings *(e.g. assessment of conduct (validity and robustness), assessment of reporting (transparency), assessment of content and utility of the findings).*	N/A – no quality appraisal
11	Appraisal items	State the tools, frameworks and criteria used to appraise the studies or selected findings *(e.g. Existing tools: CASP, QARI, COREQ, Mays and Pope* [25]*; reviewer developed tools; describe the domains assessed: research team, study design, data analysis and interpretations, reporting).*	N/A

For peer review only - http://bmjopen.bmj.com/site/about/guidelines.xhtml

12	Appraisal process	Indicate whether the appraisal was conducted independently by more than one reviewer and if consensus was required.	N/A
13	Appraisal results	Present results of the quality assessment and indicate which articles, if any, were weighted/excluded based on the assessment and give the rationale.	N/A
14	Data extraction	Indicate which sections of the primary studies were analysed and how were the data extracted from the primary studies? *(e.g. all text under the headings "results /conclusions" were extracted electronically and entered into a computer software).*	7
15	Software	State the computer software used, if any.	7
16	Number of reviewers	Identify who was involved in coding and analysis.	7
17	Coding	Describe the process for coding of data (e.g. line by line coding to search for concepts).	7
18	Study comparison	Describe how were comparisons made within and across studies (e.g. subsequent studies were coded into pre-existing concepts, and new concepts were created when deemed necessary).	7
19	Derivation of themes	Explain whether the process of deriving the themes or constructs was inductive or deductive.	7
20	Quotations	Provide quotations from the primary studies to illustrate themes/constructs, and identify whether the quotations were participant quotations or the author's interpretation.	9-15
21	Synthesis output.	Present rich, compelling and useful results that go beyond a summary of the primary studies (e.g. *new interpretation, models of evidence, conceptual models, analytical framework, development of a new theory or construct).*	9-15

For peer review only
